# Local structure preserving sparse coding for infrared target recognition

**DOI:** 10.1371/journal.pone.0173613

**Published:** 2017-03-21

**Authors:** Jing Han, Jiang Yue, Yi Zhang, Lianfa Bai

**Affiliations:** Jiangsu Key Laboratory of Spectral Imaging and Intelligent Sense, Nanjing University of Science and Technology, Nanjing, Jiangsu, China; Soochow University, CHINA

## Abstract

Sparse coding performs well in image classification. However, robust target recognition requires a lot of comprehensive template images and the sparse learning process is complex. We incorporate sparsity into a template matching concept to construct a local sparse structure matching (LSSM) model for general infrared target recognition. A local structure preserving sparse coding (LSPSc) formulation is proposed to simultaneously preserve the local sparse and structural information of objects. By adding a spatial local structure constraint into the classical sparse coding algorithm, LSPSc can improve the stability of sparse representation for targets and inhibit background interference in infrared images. Furthermore, a kernel LSPSc (K-LSPSc) formulation is proposed, which extends LSPSc to the kernel space to weaken the influence of the linear structure constraint in nonlinear natural data. Because of the anti-interference and fault-tolerant capabilities, both LSPSc- and K-LSPSc-based LSSM can implement target identification based on a simple template set, which just needs several images containing enough local sparse structures to learn a sufficient sparse structure dictionary of a target class. Specifically, this LSSM approach has stable performance in the target detection with scene, shape and occlusions variations. High performance is demonstrated on several datasets, indicating robust infrared target recognition in diverse environments and imaging conditions.

## Introduction

Automatic recognition of targets in infrared images is a challenging problem because of some inherent characteristics of the infrared image itself. Infrared images have the vignetting effect and smooth texture, contain different level of noise and pixel mixing. Infrared targets have inconsistent brightness, which is related to target orientation, surface material, etc. [1]. Besides, non-rigid targets have diverse postures, shapes, and sizes, such as humans and animals [2]. Combined with the imaging angles, scene clutters, background occlusions, and other factors [2–4], these could all be the constraints of infrared target recognition. Therefore, achieving robust target recognition with anti-interference ability (noise, fuzzification, occlusion, target shape, and scene changes) from an infrared image is still a challenging work.

Sparse representation classifier (SRC) is well applied to image classification and target recognition [5–7], which is insensitive to noise and missing data [5]. Based on SRC, JEDL obtains a simple and efficient sparse codes auto-extractor and a linear multi-class classifier from one objective function by minimizing the sparse reconstruction, discriminative sparse-code, code approximation and classification errors simultaneously [8]. But the training based methods need large samples for non-rigid targets. Indeed, there studied a series of unsupervised feature selection methods. CGSSL framework exploits the cluster analysis and structural analysis with sparsity simultaneously [9]; NSCR algorithm exploits nonnegative cluster analysis and redundancy control with row sparsity simultaneously [10]; RSSL algorithm exploits the intrinsic geometric structure of data, and the local and global structural consistencies over labels simultaneously [11]. They utilize the cluster analysis to realize a series of unsupervised feature selection methods, and belong to the re-constraint of the cluster process essentially, by adding sparsity and correlation constrains to the cluster transformation matrix. The ability of feature representation depends to some extent on the cluster effect, and there may be over-fitting of false clusters, so that the actual separability of the low dimensional features is affected.

For the practical application of infrared target detection, we study the method based on small sample or limited sample matching, which requires the sparse coding (Sc) to represent the intrinsic features of infrared targets accurately and stably. IR image has blurred texture details, pixel mixing and is affected by noise. It is necessary to study a new measure for stability analysis of the IR targets. Actually, in conditions of complex noise, blurred details and diverse imaging environments, the local essential structures of pure signals are invariable, both in sharp transitions and texture areas. We can exploit the stable prior of local spatial structure to suppress the noise and fuzzification problem, which can enhance the robustness of sparse representation in IR image. On the other hand, the local spatial structure can reflect the texture details, it can be combined with sparsity analysis to perform a more discriminative feature selection of natural IR images.

Similarly, JELSR integrates the merits of both manifold learning and sparse regression, and proposes an efficient method for unsupervised feature selection [12]. SDPE and SPPE preserving pairwise similarities between data points in addition to preserving the sparse characteristics [13]. In essence, these algorithms add the constraints of sparse mapping and classification in the objective function, or add different constraints of transformation matrix, like sparsity, on the basis of linear manifold dimension reduction methods. However, the sparseness here is mainly used to recover the noise reduction data of the original sample, which makes the essential features of dimensionality reduction more accurate.

In fact, sparsity cannot reflect the locality. As suggested in [14,15], locality is more essential than sparsity, as locality must lead to sparsity but not necessarily vice versa. However, existing local sparse methods mainly deal with the sparsity of local features in image [14] or constrain the sparsity on local dictionary bases [15]. The latter is the structured sparse, which executes the sparse constraint on group dictionary bases to solve the global similarity preserving problem smoothly [16–19]. Local dictionary constraints can enhance the stability and separability of sparse representations, but they also focus on the global feature selection. Similarly, Gao proposed a laplacian sparse coding (LSc) [20]. By adding a similarity preserving term to the formulation of classical sparse coding, LSc can preserve the global similarity of features in the sparse coding process. Peng establishes a discriminative regression approach (DR) by explicitly incorporating the discriminative information into regression in the instance space and the coefficient space jointly for high-dimensional and large-scale data [21]. We include the constraint of spatial local consistency in sparse representation process, and design an effective algorithm, to solve the corresponding optimization problem, and construct a framework to apply in the infrared object detection.

**Fig 1:**
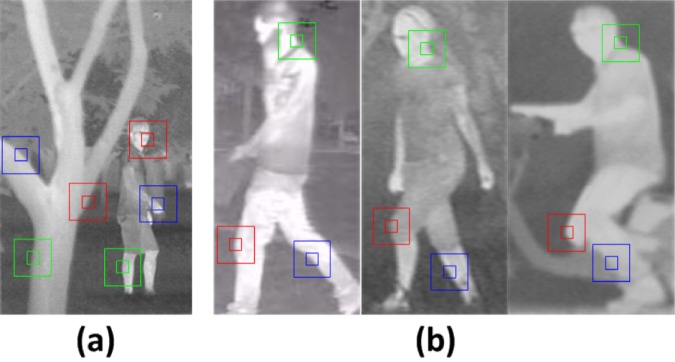
Analysis of the spatial local sparsity and structure in human targets.

For target detection analyses, we consider to learn the local areas of original samples. Though the whole structure of non-rigid samples has great differences, the characteristics in their local region are consistent. The sparse solution can provide the consistency between the test signal and training samples [22]. We divide the integral sample into small patches, the small subsamples have strong sparsity and big subsamples contain more structure information. As shown in Fig 1(A), part of patches in the trunk have similar features to the human body (small windows with same color in Fig 1(A)), which may cause the consistent sparse representations. There are quite different structures between bigger subregions in the trunk and the human body (big windows with same color in Fig 1(A)). But the various structures in big regions may cause weak sparsity. Therefore, we propose a local structure preserving sparse coding (LSPSc) algorithm to combine the sparsity and structure effectively, by adding a constraint of spatial local manifold among samples into Sc. LSPSc considers both the sparsity of small patches and the structure in big subregions, which ensures both the sparsity and locality (local area constituted by the patch and its neighborhood). Different from the above algorithms, LSPSc embeds manifold structure constraints into robust sparse representation, and the solution process of corresponding optimization problem is different.

The kernel trick can capture the nonlinear relationship from features, which may reduce the quantization error and boost the sparse coding performance. It has been pointed out that sparsity of coefficient provides no real help for the accuracy in small sample classification [23,24]. To solve this problem, Gao successfully combined kernel techniques with sparse representation [25,26] and incorporated kernel sparse coding (KSc) into a spatial pyramid matching (SPM) algorithm, which is successfully applied in image classification. Kernel sparse representation-based classification (KSRC) is then proposed [27–29]. KSRC maps the nonlinearly separable features into a high-dimensional kernel space and performs in the new space, in which complex inherent structural differences are more easily grouped together and linearly separable. SRC in kernel space can be formulated in terms of the inner products [30,31]. Cheng aims at the high-dimensional data to construct a minimax framework for multiclass classification, which can account for nonlinearity in the input space by using kernel techniques [32]. Motivated by these, we further extend LSPSc to kernel LSPSc (K-LSPSc) to adapt general nonlinear data, and design the solution procedure of optimization problem in kernel space.

Based on LSPSc and K-LSPSc, we construct a general IR target recognition method, local sparse structure matching (LSSM). Combining the discriminative feature, local matching and statistics idea, LSSM can use several template images to achieve the robust detection of IR targets. When there are occlusions, shape and scene changes, part of the local sparse structures of the target remain unchanged (Fig 1(B)), only the target area having consistent basic elements and similar structures with the template can obtain a low objective. Consequently, LSSM only requires the template set to have enough local structure and basic information of an object class, which is named as “simple template set” later. It has weak dependence on the comprehensive template images. Compared with the clustering and classification issues in [5,8,20,21], LSSM focuses on the object detection framework more, and different from the entire template matching in [33–38], LSSM is a local analysis method.

The paper is organized as follows: Section 2 details the formulation and implementation of LSPSc and K-LSPSc, presents the LSSM and the analysis of LSPSc/K-LSPSc in general infrared target recognition. In Section 3, we demonstrate the performance of LSSM with some experimental results on both visible and infrared image datasets. Finally, we conclude the paper in Section 4.

## Methods and models

### Sparse coding

Traditional sparse coding considers the maximum a posteriori estimate of the basis and coefficients, assuming a uniform prior on the basis, by solving the solution to the following optimization problem [39]:

$$\underset{B,S}{\min}\sum_{i}{\left\|x_{i}-Bs_{i}\right\|}^{2}+\lambda \sum_{i}{\left\|s_{i}\right\|}_{1},\;s.t.\;{\left\|b_{i}\right\|}^{2}\leq c,\;i=1,2,\ldots ,M$$ (1)

Denote **X** = [**x**_1_,**x**_2_,…,**x**_*N*_] ∈ *R*^*P*×*N*^ as all the samples, **B** = [**b**_1_,**b**_2_,…,**b**_*M*_] ∈ *R*^*P*×*M*^ as the dictionary matrix, and **S** = [**s**_1_,**s**_2_,…,**s**_*N*_] ∈ *R*^*M*×*N*^ as the coefficient matrix.

The L1 penalty is used to formulate the sparse coding, instead of solving the NP-hard problem of minimized L0 regularization. The first term in Eq 1 is the reconstruction error. The second term is used to control the sparsity of the sparse codes **S** and the reconstruction error through the proportion parameter *λ*. There is a norm constraint for basis to some constant *c*, which retains the variation of coefficients for each basis at the same level. The dictionary basis **B** and the sparse codes **S** should be optimized simultaneously [20,40].

### Local structure preserving sparse coding

**Fig 2:**
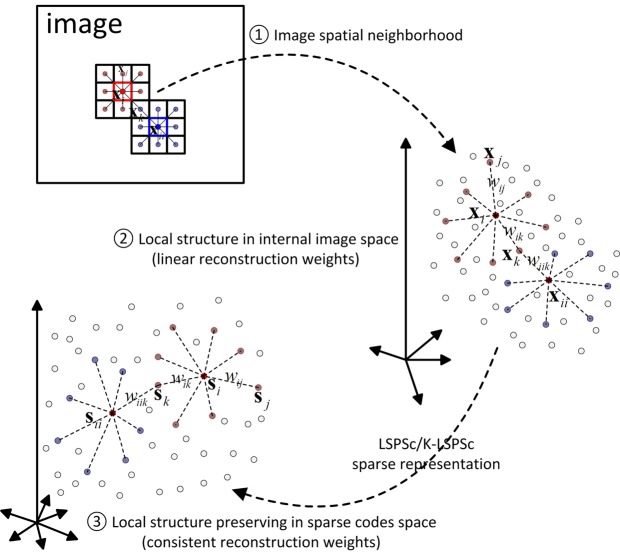
The schematic diagram of LSPSc.

The dominated sparse pursuit results in the loss of the spatial local manifold in sparse codes of the patches. To preserve such spatial locality, it is necessary to incorporate an effective prior (denoted as a regularization term) into the reconstruction process [41–43]. To maintain the structural relationship of local neighborhood in the internal image space with the corresponding sparse codes space, we explore the local manifold among patches as a prior, and introduce the neighborhood-preserved embeddings to the objective of sparse coding. As shown in Fig 2.

In the existing manifold learning methods, local linear embedding assumes that each data point and its neighbors conform to or close to a locally linear manifold [44–47], and recovers global nonlinear structure from locally linear fits [48,49]. By exploiting the local symmetries of linear reconstructions, its optimizations do not involve local minima. Inspired by these, we characterize the local geometry of the patches in image by linear coefficients that reconstruct each patch from its neighbors.

Denote the local manifold matrix corresponding to these patches as **W** ∈ **R**^*N*×*N*^, whose entry *w*_*ij*_ measures the reconstruction weights from a surrounding patch to its center patch. The weights are evaluated by minimizing the local structure reconstruction error. With the regularization prior, we can formulate the LSPSc as:

$$\underset{B,S}{\min}\sum_{i}{\left\|x_{i}-Bs_{i}\right\|}^{2}+\lambda \sum_{i}{\left\|s_{i}\right\|}_{1}+\beta \sum_{i}{\left\|s_{i}-\sum_{j}w_{ij}s_{j}\right\|}^{2}$$ (2)

subject to ‖**b**_*i*_‖^2^ ≤ *c*, *i* = 1,2,…,*M*, *c* is the constraint constant and is set to 1 in this paper. Here is **W** = [**w**_1_,**w**_2_,…,**w**_*N*_]^*T*^ and **w**_*i*_ = [*w*_*i*1_,*w*_*i*2_,…,*w*_*iN*_], which subject to the constraints $\sum_{x_{j}\in \Omega_{i}}w_{ij}=1$ and *w*_*ij*_ = 0 for any **x**_*j*_ ∉ **Ω**_*i*_. For each patch **x**_*i*_, the set **Ω**_*i*_ is its spatial *K* nearest neighborhood. Defining the constraint weights matrix of the local manifold **M** = (**I**−**W**)^*T*^(**I**−**W**), we can rewrite Eq 2 into matrix form

$$\underset{B,S}{\min}{\left\|X-\mathrm{BS}\right\|}^{2}+\lambda \sum_{i}{\left\|s_{i}\right\|}_{1}+\beta tr(\mathrm{SM}S^{T})$$ (3)

### Kernel local structure preserving sparse coding

Motivated by the nonlinear generalization performance of kernel methods, we extend LSPSc to a kernel version (K-LSPSc). An implicit mapping function *ϕ*(⋅) maps the input samples and basis to the high dimensional kernel space. K-LSPSc seeks the sparse codes for the mapped sample under the mapped basis.

$$\begin{array}{l} X\to X_{\phi }=[\phi (x_{1}),\phi (x_{2}),\ldots ,\phi (x_{N})]\\ B\to B_{\phi }=[\phi (b_{1}),\phi (b_{2}),\ldots ,\phi (b_{M})] \end{array}$$ (4)

The local manifold reconstruction weights in LSPSc should also be achieved in kernel space. In other words, we need to obtain the geometry relationships of neighboring patches in kernel space by computing the local manifold reconstruction weights among samples in **X**_*ϕ*_. The spatial neighborhood is supposed to map unchanged [50].

$$W\to W_{\phi }$$ (5)

With the local structure prior, we essentially pursue the sparse coefficients $\hat{S}$ of LSPSc in kernel space. The formulation of K-LSPSc is expressed as in Eq 6, subject to ‖*ϕ*(**b**_*i*_)‖^2^ ≤ *c*, *i* = 1,2,…,*M*. We also constraint $\sum_{x_{j}\in \Omega_{i}}w_{\phi ij}=1$ in kernel space.

$$\underset{B_{\phi },\hat{S}}{\min}{\left\|X_{\phi }-B_{\phi }\hat{S}\right\|}^{2}+\lambda \sum_{i}{\left\|{\hat{s}}_{i}\right\|}_{1}+\beta \sum_{i}{\left\|{\hat{s}}_{i}-\sum_{j}w_{\phi ij}{\hat{s}}_{j}\right\|}^{2}$$ (6)

#### Relationship between LSPSc and K-LSPSc

In kernel space, samples in different categories are linearly separable, which enhances the discriminative ability of the sparse coding and boosts target recognition performance. On the other hand, LSPSc assumes that the local structure of the patches in natural image satisfies a linear relationship, but there are more nonlinear structures in practical image data. K-LSPSc supposes local patches in image to meet the linear reconstruction in high dimensional kernel space. Thus, when the data in kernel space are nonlinearly mapped to a low dimensional space, the data structure is complex, which is according with the distribution of actual data. Therefore, by the kernel skill the local reconstruction error may be reduced, and we can get the structure sparse quantization for the signals more accurate and discriminative.

### The implementation of LSPSc and K-LSPSc

The optimization problems in Eq 2 and Eq 6 can be solved in two core aspects: the calculation of structure matrix (**W** and **W**_*ϕ*_) and optimization scheme of sparse coefficient and dictionary (**S**, **B** and $\hat{S}$, **B**_*ϕ*_).

#### *Calculation of* W *and* W_*ϕ*_

The local manifold reconstruction weights **W** from the neighbors {**x**_*j*_ | *j* = 1,2,…,*K*} to a center patch **x**_*i*_ are computed by minimizing the reconstruction error, which is measured by the cost function:

$$\underset{W}{\min}\sum_{i=1}^{N}{\left\|x_{i}-\sum_{j=1}^{K}w_{ij}x_{j}\right\|}^{2},\;s.t.\;\sum_{j}w_{ij}=1$$ (7)

Here is **W** = [**w**_1_,**w**_2_,…,**w**_*N*_] and **w**_*i*_ = [*w*_*i*1_,*w*_*i*2_,…,*w*_*iK*_]^*T*^. Minimizing the cost function subject to the constraint **e**^*T*^**w**_*i*_ = 1 is a constrained least squares problem [47–49].

Setting $f(w_{i})={\left\|x_{i}-\sum_{j=1}^{K}w_{ij}x_{j}\right\|}^{2}$ and **G**_*i*_ = [**x**_*i*_−**x**_*i*1_,…,**x**_*i*_−**x**_*iK*_], there is $f(w_{i})={\left\|G_{i}w_{i}\right\|}^{2}=w_{i}^{T}G_{i}^{T}G_{i}w_{i}$. So the optimization problem in Eq 7 can be converted as $\min\;w_{i}^{T}G_{i}^{T}G_{i}w_{i}$ subject to **e**^*T*^**w**_*i*_ = 1. Consider the Lagrangian $l(w_{i})=w_{i}^{T}G_{i}^{T}G_{i}w_{i}+\eta (w_{i}^{T}e-1)$. The optimal weights are gained by requiring the first derivative of $l(w_{i})$ versus **w**_*i*_ to be zero: $\partial l(w_{i})/\partial w_{i}=G_{i}^{T}G_{i}w_{i}+\eta e=0$. Then solve Eq 8 and rescale the weights to get the desired solution **W**. Here is $G_{i}^{T}G_{i}={\left[(x_{i}-x_{it1})(x_{i}-x_{it2})\right]}_{t1,t2=1,\ldots ,K}$.

$$G_{i}^{T}G_{i}w_{i}=e$$ (8)

In kernel space, each sample **x**_*i*_ is transformed to *ϕ*(**x**_*i*_). The reconstruction error can then be minimized as follows:

$$\underset{W_{\phi }}{\min}\sum_{i=1}^{N}{\left\|\phi (x_{i})-\sum_{j=1}^{K}w_{\phi ij}\phi (x_{j})\right\|}^{2},\;s.t.\;\sum_{j}w_{\phi ij}=1$$ (9)

Setting **w**_*ϕi*_ = [*w*_*ϕi*1_,*w*_*ϕi*2_,…,*w*_*ϕiK*_]^*T*^, **G**_*ϕi*_ = [*ϕ*(**x**_*i*_)−*ϕ*(**x**_*i*1_),…,*ϕ*(**x**_*i*_)−*ϕ*(**x**_*iK*_)]. In accordance with the above principle, the desired solution in kernel space **W**_*ϕ*_ can be obtained by solving $G_{\phi i}^{T}G_{\phi i}w_{\phi i}=e$ and rescaling the weights. In kernel trick, the kernel function is defined as the inner product of two mapping functions *k*(*x*,*y*) = ⟨*ϕ*(*x*),*ϕ*(*y*)⟩, which should be positive definite. So there is $G_{\phi i}^{T}G_{\phi i}={\left[1\text{-}k(x_{i},x_{i_{t1}})-k(x_{i},x_{i_{t2}})+k(x_{i_{t1}},x_{i_{t2}})\right]}_{t1,t2=1,\ldots ,K}$.

#### Implementation of LSPSc and K-LSPSc

DR solves the optimization based on proximal point algorithm and accelerated proximal gradient line search method, which has high computational cost. We employ the efficient strategy of feature-sign search algorithm in [39] to solve the optimization objective of Eq 3 by alternately optimizing **B** and **S** while holding the other fixed. K-LSPSc is the same as LSPSc except for the kernel mapping. Thus, we can use the same method to optimize the objective of K-LSPSc.

*First*, *learn the coefficients* **S** *and* $\hat{S}$. In LSPSc, **B** is fixed and the optimization problem of Eq 2 is equivalent to a regularized least squares problem. We optimize each **s**_*i*_ individually with all the remaining sparse codes **s**_*j*_(*j* ≠ *i*) fixed in Eq 10. By replacing the sparse reconstruction error term in [39] with *J*(**s**_*i*_), the efficient sparse coding algorithm can be easily extended to optimize LSPSc coefficients in Eq 10.

$$\begin{array}{c} \underset{s_{i}}{\min}J(s_{i})+\lambda {\left\|s_{i}\right\|}_{1}\\ J(s_{i})={\left\|x_{i}-Bs_{i}\right\|}^{2}+\beta {\left\|s_{i}-\sum_{j}w_{ij}s_{j}\right\|}^{2} \end{array}$$ (10)

Due to the constraint $\sum_{j}w_{ij}=1$, there is $s_{i}{}^{T}s_{i}=\sum_{j}w_{ij}s_{i}{}^{T}s_{i}$, so *J*(**s**_*i*_) can be transformed into Eq 11.

$$J(s_{i})=s_{i}{}^{T}B^{T}Bs_{i}-x_{i}{}^{T}Bs_{i}-s_{i}{}^{T}B^{T}x_{i}+\beta [s_{i}{}^{T}(SL_{i})+{\left(SL_{i}\right)}^{T}s_{i}-s_{i}{}^{T}L_{ii}s_{i}]+f(s_{j})$$ (11)

Here, $f(s_{j})=x_{i}{}^{T}x_{i}+\beta {\left(\sum_{j}w_{ij}s_{j}\right)}^{T}(\sum_{j}w_{ij}s_{j})$ and **L** = **I**−**W**, where **I** is an identity matrix. **L**_*i*_ is the *i*^*th*^ column of matrix **L**. Actually, **L**_*ii*_ = 1 due to *w*_*ii*_ = 0. The details of solving Eq 10 are listed in Algorithm 1, in which the first and second derivative of *J*(**s**_*i*_) versus **s**_*i*_ can be expressed as Eq 12.

$$\begin{array}{l} \frac{\partial J(s_{i})}{\partial s_{i}}=-2B^{T}x_{i}+2B^{T}Bs_{i}+\beta (2S_{-i}L_{i,-i}-2L_{ii}s_{i})\\ \frac{\partial^{2}J(s_{i})}{\partial s_{i}{}^{2}}=2B^{T}B-2\beta L_{ii}I \end{array}$$ (12)

Here, **S**_−*i*_ is the submatrix after removing the *i*^*th*^ column from **S**, and **L**_*i*,−*i*_ is the subvector after removing the *i*^*th*^ entry from **L**_*i*_. Some recent works show that the coefficients **S** initialized with some heuristics can achieve faster convergence [20]. Thus, **S** is initialized with the results of general sparse coding in Algorithm 1.

In K-LSPSc, Eq 10 should be implemented in kernel space, which can be expressed as follows: $S\to \hat{S}$, $J(s_{i})\to J_{\phi }({\hat{s}}_{i})$.

$$J_{\phi }({\hat{s}}_{i})={\left\|\phi (x_{i})-B_{\phi }{\hat{s}}_{i}\right\|}^{2}+\beta {\left\|{\hat{s}}_{i}-\sum_{j}w_{\phi ij}{\hat{s}}_{j}\right\|}^{2}.$$ (13)

There is also the constraint $\sum_{j}w_{\phi ij}=1$ in kernel space, so $J_{\phi }({\hat{s}}_{i})$ can be further transformed to Eq 14.

$$J_{\phi }({\hat{s}}_{i})={\hat{s}}_{i}{}^{T}B_{\phi }^{T}B_{\phi }{\hat{s}}_{i}-\phi {\left(x_{i}\right)}^{T}B_{\phi }{\hat{s}}_{i}-{\hat{s}}_{i}{}^{T}B_{\phi }^{T}\phi (x_{i})+\beta [({\hat{s}}_{i}{}^{T}(\hat{S}L_{\phi i})+{\left(\hat{S}L_{\phi i}\right)}^{T}{\hat{s}}_{i}-{\hat{s}}_{i}{}^{T}L_{\phi ii}{\hat{s}}_{i})]+f({\hat{s}}_{j}).$$ (14)

Here, $f({\hat{s}}_{j})=\phi {\left(x_{i}\right)}^{T}\phi (x_{i})+\beta {\left(\sum_{j}w_{\phi ij}{\hat{s}}_{j}\right)}^{T}(\sum_{j}w_{\phi ij}{\hat{s}}_{j})$ and **L**_*ϕ*_ = **I**−**W**_*ϕ*_. With the kernel trick, we have $B_{\phi }^{T}B_{\phi }={\left\{\phi {\left(b_{m}\right)}^{T}\phi (b_{n})\right\}}_{m,n=1,2,\ldots ,M}$, *ϕ*(**b**_*m*_)^*T*^*ϕ*(**b**_*n*_) = ⟨**b**_*m*_,**b**_*n*_⟩ = *k*(**b**_*m*_,**b**_*n*_) and $B_{\phi }^{T}\phi (x_{i})={\left[\phi {\left(b_{1}\right)}^{T}\phi (x_{i}),\ldots ,\phi {\left(b_{m}\right)}^{T}\phi (x_{i}),\ldots ,\phi {\left(b_{M}\right)}^{T}\phi (x_{i})\right]}_{m=1,2,\ldots ,M}$, *ϕ*(**b**_*m*_)^*T*^*ϕ*(**x**_*i*_) = ⟨**b**_*m*_,**x**_*i*_⟩ = *k*(**b**_*m*_,**x**_*i*_). All these subitems are calculable, so the efficient sparse coding algorithm can also be employed to get KLSPSc coefficients $\hat{S}$.

*Second*, *learn the dictionary basis* **B** *and* **B**_*ϕ*_. In LSPSc, **S** is fixed and the optimization problem of Eq 3 can be rewritten as Eq 15, which is a least squares problem with quadratic constraints. Following [39], by solving a Lagrange dual through conjugate gradient method, we optimize the basis **B** as Eq 16.

$$\underset{B}{\min}{\left\|X-\mathrm{BS}\right\|}^{2},\;s.t.\;{\left\|b_{i}\right\|}^{2}\leq c,\;i=1,2,\ldots ,M$$ (15)

$$B^{T}={\left(SS^{T}+\Lambda \right)}^{-1}{\left(XS^{T}\right)}^{T}$$ (16)

Here, **Λ** is a diagonal matrix of the dual variable, which is set as an identity matrix in this paper.

In K-LSPSc, the optimization problem of Eq 6 can be solved with the same method, and the basis **B**_*ϕ*_ is optimized as:

$$B_{\phi }^{T}={\left(\hat{S}{\hat{S}}^{T}+\Lambda \right)}^{-1}{\left(\phi (X){\hat{S}}^{T}\right)}^{T}$$ (17)

It can be seen that the updated basis in kernel space **B**_*ϕ*_ cannot be obtained numerically for the implicit mapping function in Eq 17. Actually, in the *n*^*th*^ $\hat{S}$ updating iteration by the efficient sparse coding algorithm, we can use the result of **B**_*ϕ*_ in the (*n*−1)^*th*^ updating iteration to compute the part in Eq 14: $B_{\phi }^{T}\phi (x_{i})={\left[{\left(\hat{S}{\hat{S}}^{T}+\Lambda \right)}^{-1}{\left(\phi (X){\hat{S}}^{T}\right)}^{T}\right]}_{last}\phi (x_{i})={\left[{\left(\hat{S}{\hat{S}}^{T}+\Lambda \right)}^{-1}\hat{S}\right]}_{last}\phi {\left(X\right)}^{T}\phi (x_{i})={\left[{\left(\hat{S}{\hat{S}}^{T}+\Lambda \right)}^{-1}\hat{S}\right]}_{last}[k(x_{1},x_{i}),\ldots ,k(x_{i},x_{i}),\ldots ,k(x_{N},x_{i})]$ and $B_{\phi }^{T}B_{\phi }={\left[{\left(\hat{S}{\hat{S}}^{T}+\Lambda \right)}^{-1}{\left(\phi (X){\hat{S}}^{T}\right)}^{T}\right]}_{last}{\left[{\left(\hat{S}{\hat{S}}^{T}+\Lambda \right)}^{-1}{\left(\phi (X){\hat{S}}^{T}\right)}^{T}\right]}_{last}^{T}={\left[{\left(\hat{S}{\hat{S}}^{T}+\Lambda \right)}^{-1}\hat{S}\right]}_{last}\phi {\left(X\right)}^{T}\phi (X){\left[{\left(\hat{S}{\hat{S}}^{T}+\Lambda \right)}^{-1}\hat{S}\right]}_{last}^{T}$.

Different with the linear kernel used in DR, we choose the RBF kernel $k(x,y)=\exp(-{\left\|x-y\right\|}^{2}/\sigma_{k}^{2})$, which can estimate the nonlinear similarity between two signals. The Gaussian variance *σ*_*k*_ is a constant and is set as 1.

**Algorithm 1** Pseudocode for Feature-Sign Search Algorithm in Solving Eq 10

    1: **Input**: **x**_*i*_ the *i*^*th*^ sample in **X**; the dictionary **B**; initial sparse coding **S** and *λ*, *β* of LSPSc.

        **Output**: The LSPSc sparse codes **s**_*i*_ of sample **x**_*i*_.

    2: **Initialize**: **S** is initialized as the classical sparse coding, sign set **θ** = *sign*(**s**_*i*_), active set **Ω** = *find*(**s**_*i*_ ≠ 0).

    3: Calculate the local manifold reconstruction weights **w**_*i*_ by Eq 8 to get **L**_*i*_.

    4: From zero coefficients of **s**_*i*_, select $rm=\arg\;\underset{r}{\max}\left|\Delta_{s_{i}}^{r}\right|$, $\Delta_{s_{i}}=\partial J(s_{i})/\partial s_{i}$. Denote **v**^*r*^ as the *r*^*th*^ entries of vector **v**.

            If $\Delta_{s_{i}}{}^{rm}>\lambda$, set **θ**^*rm*^ = −1, active set **Ω** = {*rm*}∪**Ω**.

            If $\Delta_{s_{i}}{}^{rm}<-\lambda$, set **θ**^*rm*^ = 1, active set **Ω** = {*rm*}∪**Ω**.

    5: **Feature-sign step**:

            Compute the analytical solution to the resulting unconstrained QP: $\underset{s_{i\;\Omega }}{\min}J(s_{i\;\Omega })+\lambda \theta_{\Omega }{}^{T}s_{i\;\Omega }$.

                $s_{i\;\Omega \;new}={\left({\left(\Delta_{s_{i}s_{i}}\right)}_{\Omega }\right)}^{-1}(2B_{\Omega }{}^{T}x_{i}-2\beta {\left(S_{-i}L_{i,-i}\right)}_{\Omega }-\lambda \theta_{\Omega })$

                Denote **v**_**Ω**_ as the subvector of vector **v** corresponding to the active set **Ω**, and **M**_**Ω**_ as the submatrix of **M** that contains only the columns corresponding to the active set **Ω**. $\Delta_{s_{i}s_{i}}=\partial^{2}J(s_{i})/\partial s_{i}{}^{2}$.

            Perform a discrete line search on the closed line segment from **s**_*i***Ω**_ to **s**_*i***Ω***new*_:

                Check the objective value at **s**_*i***Ω***new*_ and all points where any coefficient changes sign.

                Update **s**_*i***Ω**_ (and the corresponding entries in **s**_*i***Ω**_) to the point with the lowest objective value.

            Remove zero coefficients of **s**_*i***Ω**_ from the active set **Ω** and update **θ** = *sign*(**s**_*i*_).

    6: **Check the optimality conditions**:

            (a) Optimality condition for nonzero coefficients: $\Delta_{s_{i}}{}^{r}+\lambda sign(s_{i}{}^{r})=0$, ∀**s**_*i*_^*r*^ ≠ 0.

                If condition (a) is not satisfied, go to Step 5 (without any new activation); else check condition (b).

            (b) Optimality condition for zero coefficients: $\left|\Delta_{s_{i}}{}^{r}\right|\leq \lambda$, ∀**s**_*i*_^*r*^ ≠ 0.

                If condition (b) is not satisfied, go to Step 4; otherwise return **s**_*i*_ as the solution, and update the **S** of LSPSc with current **s**_*i*_.

In Algorithm 1, each step reduces the objective $f(s_{i})=J(s_{i})+\lambda {\left\|s_{i}\right\|}_{1},J(s_{i})={\left\|x_{i}-Bs_{i}\right\|}^{2}+\beta {\left\|s_{i}-\sum_{j}w_{ij}s_{j}\right\|}^{2}$, and that the overall algorithm always converges to the optimal solution Eq 10. Consider optimization problem Eq 10 augmented with the additional constraint that **s**_*i*_ is consistent with a given active set and sign vector. If the current coefficients **s**_*ic*_ are consistent with the active set and sign vector, but are not optimal for the augmented problem at the start of Step 5, the feature-sign step is guaranteed to strictly reduce the objective; If the coefficients **s**_*ic*_ at the start of Step 4 are optimal for the augmented problem, but are not optimal for problem Eq 10, the feature-sign step is guaranteed to strictly reduce the objective. The algorithm converges to a global optimum of the optimization problem Eq 10 in a finite number of steps.

1. Let ${\hat{s}}_{ic}$ be the subvector of **s**_*ic*_ corresponding to coefficients in the given active set **Ω**. In Step 5, consider a smooth quadratic function $\tilde{f}({\hat{s}}_{i})={\left\|x_{i}-\hat{B}{\hat{s}}_{i}\right\|}^{2}+\beta {\left\|{\hat{s}}_{i}-\sum_{ij}w_{ij}{\hat{s}}_{j}\right\|}^{2}+\lambda {\hat{\theta }}^{T}{\hat{s}}_{i}$. Since ${\hat{s}}_{ic}$ is not an optimal point of $\tilde{f}$, we have $\tilde{f}({\hat{s}}_{i\;new})<\tilde{f}({\hat{s}}_{i\;c})$. Now consider the two possible cases: (1) if ${\hat{s}}_{i\;new}$ is consistent with the given active set and sign vector, updating ${\hat{s}}_{i}≔{\hat{s}}_{i\;new}$ strictly decreases the objective; (2) if ${\hat{s}}_{i\;new}$ is not consistent with the given active set and sign vector, let ${\hat{s}}_{id}$ be the first zero-crossing point (where any coefficient changes its sign) on a line segment from ${\hat{s}}_{ic}$ to ${\hat{s}}_{i\;new}$, then clearly ${\hat{s}}_{i\;c}\neq {\hat{s}}_{i\;d}$, and $\tilde{f}({\hat{s}}_{id})<\tilde{f}({\hat{s}}_{ic})$ by convexity of $\tilde{f}$, thus we finally have $f({\hat{s}}_{id})=\tilde{f}({\hat{s}}_{id})<\tilde{f}({\hat{s}}_{ic})=f({\hat{s}}_{ic})$. Therefore, the discrete line search described in Step 5 ensures a decrease in the objective value.
2. Since **s**_*ic*_ is optimal for the augmented problem, it satisfies optimality condition (a), but not (b); thus, in Step 4, there is some *r*, such that $\left|\Delta_{s_{i}}{}^{r}\right|>\lambda$; this *r*-th coefficient is activated, and *r* is added to the active set **Ω**. In Step 5, consider the smooth quadratic function $\tilde{f}({\hat{s}}_{i})={\left\|x_{i}-\hat{B}{\hat{s}}_{i}\right\|}^{2}+\beta {\left\|{\hat{s}}_{i}-\sum_{ij}w_{ij}{\hat{s}}_{j}\right\|}^{2}+\lambda {\hat{\theta }}^{T}{\hat{s}}_{i}$. Observe that (1) since a Taylor expansion of $\tilde{f}$ around ${\hat{s}}_{i\;}={\hat{s}}_{ic}$ has a first order term in **s**_*i*_^*r*^ only (using condition 6(a) for the other coefficients), we have that any direction that locally decreases $\tilde{f}({\hat{s}}_{i})$ must be consistent with the sign of the activated **s**_*i*_^*r*^, and, (2) since ${\hat{s}}_{ic}$ is not an optimal point of $\tilde{f}({\hat{s}}_{i})$, $\tilde{f}({\hat{s}}_{i})$ must decrease locally near ${\hat{s}}_{i}={\hat{s}}_{ic}$ along the direction from ${\hat{s}}_{ic}$ to ${\hat{s}}_{i\;new}$. From Eq 1 and Eq 2, the line search direction ${\hat{s}}_{ic}$ to ${\hat{s}}_{i\;new}$ must be consistent with the sign of the activated **s**_*i*_^*r*^. Finally, since $\tilde{f}({\hat{s}}_{i})=f({\hat{s}}_{i})$ when ${\hat{s}}_{i}$ is consistent with the active set **Ω**, either ${\hat{s}}_{i\;new}$ is consistent, or the first zero-crossing from ${\hat{s}}_{ic}$ to ${\hat{s}}_{i\;new}$ has a lower objective value (similar argument to A).
3. From the above arguments, it follows that the steps always strictly reduce the objective *f*(**s**_*i*_). At the start of Step 4, **s**_*i*_ either satisfies optimality condition 6(a) or is $\vec{0}$; in either case, **s**_*i*_ is consistent with the current active set and sign vector, and must be optimal for the augmented problem described in the above arguments. Since the number of all possible active sets and coefficient signs is finite, and since no pair can be repeated (because the objective value is strictly decreasing), the outer loop of Steps 4-6(b) cannot repeat indefinitely. Now, it suffices to show that a finite number of steps is needed to reach Step 6(b) from Step 4. This is true because the inner loop of Steps 5-6(a) always results in either an exit to Step 6(b) or a decrease in the size of the active set.

### Local sparse structure matching model

**Fig 3:**
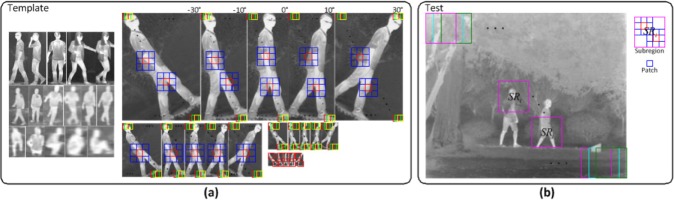
Diagram of the LSSM for human recognition. (a) Sampling from the expanded template set for learning the local sparse structure dictionary; (b) LSSM target recognition based on LSPSc.

**Fig 4:**
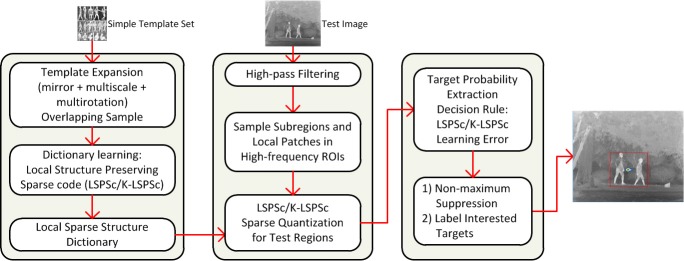
Framework of the LSSM model.

The local sparse structure matching (LSSM) model based on LSPSc and K-LSPSc is designed for robust recognition of general infrared targets. Fig 3 illustrates this idea using human recognition as an example. Fig 4 shows an overview of our proposed framework, which mainly includes dictionary generation, sparse quantization for test regions, and target probability extraction.

#### Local sparse structure dictionary generation

Expand the simple template set by scaling and rotating every image and its mirror in the template set (Fig 3(A)), we can learn the dictionary of interested targets with multi-scale and multi-rotation information. The scale is from 0.2 to 2 by steps of 0.2; the rotation is from −90° to 90° by steps of 10°. Obviously it is time consuming, but the dictionary is pre-learned, which is before sparse quantization of test images. It has no effect on the efficiency of target identification, but improves the robustness of the dictionary.

Sample the patches (small windows in Fig 3(A)) by pixels (with step of one pixel) from the expanded template images. Fix each patch size *p*_*s*_ at 12 and consider its nonoverlapping neighbors. Calculate the neighborhood reconstruction weights among each patch and its eight neighboring patches by Eq 8. We iteratively optimize the sparse coefficients (by Algorithm 1) and dictionary (by Eq 16) while holding the other fixed.

#### LSPSc/K-LSPSc sparse quantization and target probability extraction

Take a similar approach to sample subregions (large windows in Fig 3(B)) from a test image, which is mainly used to study the local sparsity and structure information. The subregion size *r*_*s*_ is set as 60 by considering both sparsity and locality. Each subregion is divided into a set of nonoverlapping patches (same size as template patches). Using the local sparse structure dictionary of the template, the sparse representation of all patches in subregion is quantized according to LSPSc/K-LSPSc. Through the K-LSPSc dictionary $B_{\phi }={\left[\phi (X){\hat{S}}^{T}{\left(\hat{S}{\hat{S}}^{T}+\Lambda \right)}^{-1}\right]}_{template}$ is nonnumeric, the expression $B_{\phi }^{T}\phi (x_{i})$ and $B_{\phi }^{T}B_{\phi }$ in $\Delta_{{\hat{s}}_{i}}$ and $\Delta_{{\hat{s}}_{i}{\hat{s}}_{i}}$ in Algorithm 1 can be formulated as $B_{\phi }^{T}\phi (x_{i})={\left[{\left(\hat{S}{\hat{S}}^{T}+\Lambda \right)}^{-1}\hat{S}\right]}_{template}k(X_{template},x_{i})$ and $B_{\phi }^{T}B_{\phi }={\left[{\left(\hat{S}{\hat{S}}^{T}+\Lambda \right)}^{-1}\hat{S}\right]}_{template}k(X_{template},X_{template}){\left[{\hat{S}}^{T}{\left(\hat{S}{\hat{S}}^{T}+\Lambda \right)}^{-1}\right]}_{template}$, they are calculable and numerical. Here, **x**_*i*_ is a sample from test patches.

We present a new definition of target probability for each subregion, which is used to determine the matching degree between test local region and template. The target probability *ρ*_*t*_ is defined by the average LSPSc/K-LSPSc objective value H_*t*_ of all patches in each subregion *SR*_*t*_, as in Eq 17. H_*t*_ is also called “matching error” hereinafter.

$$\begin{array}{c} H_{t}=\frac{1}{N}\sum_{SR_{t}}\left\{\underset{S}{\min}{\left\|X-\mathrm{BS}\right\|}^{2}+\lambda \sum_{i}{\left\|s_{i}\right\|}_{1}+\beta tr(\mathrm{SM}S^{T})\right\}\\ \rho_{t}=\exp(-H_{t}{}^{2}/2\sigma^{2}) \end{array}$$ (18)

Here **X** is a sample set of all patches in a test subregion. The target probability in the kernel skill is formulated as:

$$\begin{array}{c} {\hat{H}}_{t}=\frac{1}{N}\sum_{SR_{t}}\left\{\underset{\hat{S}}{\min}{\left\|X_{\phi }-B_{\phi }\hat{S}\right\|}^{2}+\lambda \sum_{i}{\left\|{\hat{s}}_{i}\right\|}_{1}+\beta tr(\hat{S}M_{\phi }{\hat{S}}^{T})\right\}\\ {\hat{\rho }}_{t}=\exp(-{\hat{H}}_{t}{}^{2}/2{\hat{\sigma }}^{2}) \end{array}$$ (19)

The Gaussian variables *σ* and $\hat{\sigma }$ control the degree of attenuation from target probability to matching error value. They are set as the average LSPSc/K-LSPSc matching error according to target category, and are regarded as priori constants in LSSM. After the target probability tests are performed, we employ the approaches of significance tests and non-maxima suppression [51] for final detection.

The local sparse structure dictionary contains sparse elements and spatial local manifolds of template. From Eqs 18 and 19, the second term constrains the consistency between test patches and template basis; the third term preserves the consistent neighborhood reconstruction relationship between test patches and their sparse codes. The sparse coefficient **S** and $\hat{S}$ rely on the template dictionary, while the constraint weights of local structure **M** and **M**_*ϕ*_ rely on test regions, so the minimum of the third term ensures a similar local sparse structure between test regions and template set. Therefore, only the test regions, which have patches matching with template dictionary basis and local sparse structure similar with template subregions both, deserve low sparse quantization error (first term), few dictionary basis response (second term), and low constrained local sparse structure error (third term). Then these regions get large target probability.

### Analysis of LSPSC/K-LSPSc in LSSM

#### Analysis of the ability of similarity preserving by LSPSc

**Fig 5:**
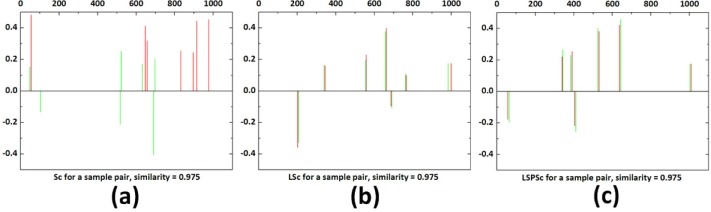
Comparison of the sparse codes of similar samples (a pair of neighboring patches with the local reconstruction weight of 0.975) by Sc, LSc, and LSPSc.

**Fig 6:**
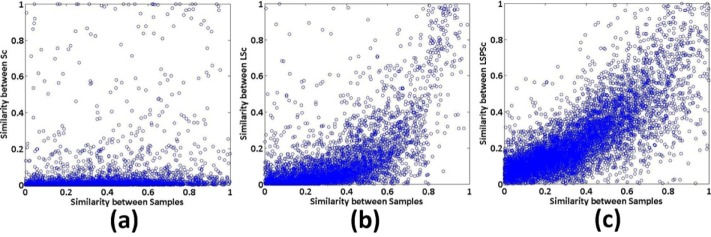
Analysis of the similarity preserving performance of Sc, LSc, and LSPSc. The X-axis is the similarity between original samples, evaluated by local reconstruction weights of spatial neighboring patches. The Y-axis is the similarity between sparse codes, evaluated by local reconstruction weights of the sparse codes corresponding to spatial neighboring samples.

As a local sparse matching approach, our model requires the stability of sparse representation. It should suppress the noise interference and constrain the consistency of sparse representations among similar samples. LSc ensures that sparse representations of global similar samples are consistent, but the local different information among patches is neglected. So it is sensitive to noise and change of background. In contrast, the main contribution of our formulation is the locality preservation, including both the similarity and non-similarity among spatial local patches in images, as illustrated in Figs 5 and 6.

The *Caltech 101* dataset (http://www.vision.caltech.edu/Image_Datasets/Caltech101/), containing 101 categories, is used to demonstrate the principle of our formulation, which is compared with the classical sparse coding Sc (by OMP) and group sparse coding algorithms (LSc). We fix the dictionary size at 1,024; *λ* 0.3, and *β* 0.2. The detailed analysis of parameters can be found in the next section. Template patches are dense grid sampled (template patch number is 1.5 × 10^4^) from 10 randomly selected images in the *Lotus* category, and 9.0 × 10^4^ test patches are sampled from the rest 56 images. All the template patches are used to learn the *lotus* dictionaries by Sc, LSc, and LSPSc. Then, a pair of patches with maximum neighborhood weight of 0.975 selected from test patches are sparse represented by Sc, LSc, and LSPSc according to the corresponding dictionary. The sparse representation of the two samples is one with red and one with green. Fig 5(A), 5(B) and 5(C) correspond to different sparse coding methods, in which higher the red and green coincidence degree indicates that the sparse representation consistency of the two is higher. It can be seen that the sparse features of similar patches by Sc vary a lot; they are very similar by LSc; the similarity between sparse features by LSPSc is not as good as that of LSc, but they are roughly consistent.

To further evaluate the non-similarity preserving ability of LSPSc, we dense grid sample template patches (patch number is 1.74 × 10^4^) from the 30 randomly selected images in the *Leopards* category, 9.86 × 10^4^ test patches from the remaining 170 images in this category. To compare three algorithms in Fig 6, we calculate and plot the spatial local reconstruction weights of original test patches as the similarity between samples in the X-axis, and the local reconstruction weights of their sparse codes as the similarity between sparse features by Sc/LSc/LSPSc in the Y-axis.

The similarity among original patches is totally destroyed by Sc in (a). Because kNN (k nearest neighbors) is used to compute the similarity weights from global patches in LSc, the similarity constraint between highly similar patches is strong and the similarity is preserved well in their sparse codes. But the constraint between patches with weak similarity is ignored, which may lead to dissimilar samples having similar sparse codes on the sample manifold. In particular, although it is a global similarity constraint in LSc but a local similarity used in (b), it does not impair the performance of LSc due to the equivalence from local similarity to global similarity. LSPSc constrains the preservation of the local sparse structure by keeping the spatial neighborhood reconstruction weights among samples into their sparse codes. The consistent neighborhood reconstruction weights in input space and sparse code space indicate that when the weight from neighbor to center patch is big (highly similar patches), the corresponding sparse codes are similar; when weight is small (dissimilar patches) the corresponding sparse codes are dissimilar. So the constraint in LSPSc contains both similarity and dissimilarity. Therefore, (c) shows a more evident linear trend of the similarity between sparse codes against the similarity between patches. It proves that LSPSc can preserve the local structure of original samples well.

#### Analysis of the performance in infrared target representation by LSPSC/K-LSPSc

Based on the well property of LSPSc in visible images, we further use an *Infrared Human* dataset (The *Infrared Human* dataset is captured by FILR Tau 2–640 and Tau 2–324 Uncooled LWIR Thermal Imaging Cameras, including total 224 images with 162 images containing human and 62 images not containing human in different scenes.) to evaluate the performance of LSPSc and K-LSPSc. In this dataset, we fix the dictionary size at 256, *λ* is 0.4, and *β* is 0.3.

**Fig 7:**
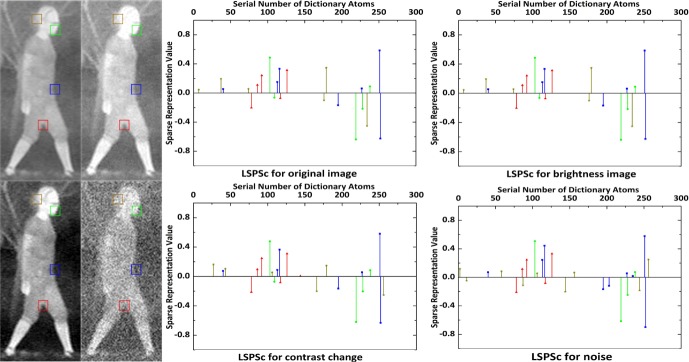
The invariance and robustness of LSPSc in brightness, contrast, and noise changes.

Test the robustness of LSPSc sparse quantization in infrared image with changing brightness, contrast, and noise. The LSPSc and K-LSPSc dictionaries of *infrared human* are learned from the simple template set in Fig 3(A). In Fig 7, LSPSc sparse representations of brightness changed infrared patches are totally invariant, the contrast changed and noise added infrared patches are slightly influenced. The influence is weak on patches with relatively complex neighborhood structure (green and red), is stronger on patches with simple structure in their neighborhood (blue), and is more prominent on patches with no obvious structure (brown). Because the local sparse structure of target remains unchanged in condition of different environments, more interdependent neighborhood reconstruction relationships indicate a stronger local spatial manifold constrain, which can weaken the interference of noise, imaging blurring and background, and enhance the robustness of sparse quantization in infrared images.

**Fig 8:**
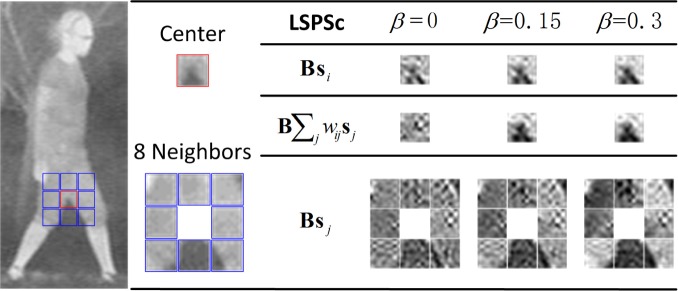
Analysis of spatial local manifold preserving property by LSPSc. The dictionary size is set at 256, *λ* is 0.4.

Based on the *infrared human* dictionaries, we measure the local structure preserving by LSPSc in infrared images. In Fig 8, we present the sparse decoding of the center patch (row 2) and its 8 neighboring patches (row 4) by LSPSc. When *β* = 0 LSPSc regresses to Sc. The details in infrared image are fuzzy, stronger constraint of noise and blur invariant structure features can improve the accuracy of sparse decomposition. It is obvious that a bigger *β* executes a stronger constraint of the spatial local manifold, and the sparse decoding of center patch (row 2) is more similar with the reconstitution from its neighbors (row 3). However, an overlarge *λ* or *β* will make it hard for the optimization problem to meet both sparsity and structure preserving simultaneously. This will lead the objective to be nonconvergent or a big convergence value.

**Fig 9:**
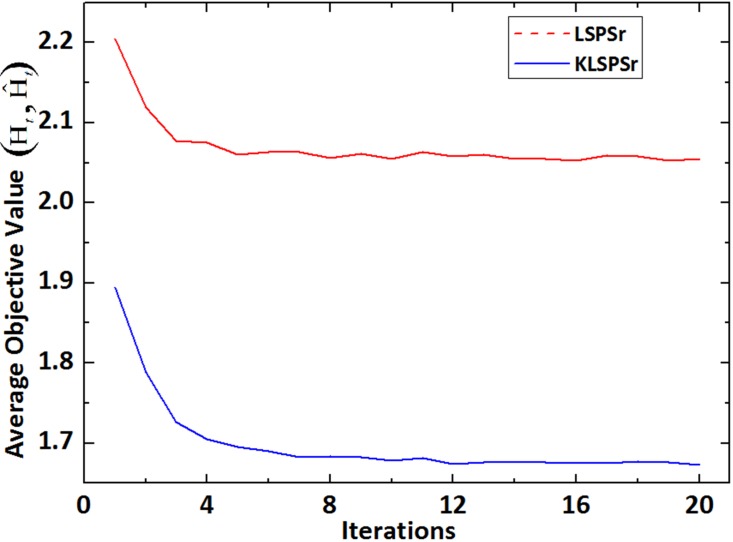
Comparison of convergence between LSPSc and K-LSPSc. The average objective value in Y-axis is the mean of LSPSc and K-LSPSc objectives of all patches in Fig 7, whereas the X-axis shows the iterations.

By the kernel skill, K-LSPSc optimizes the linear assumption for actual data in LSPSc. So under the condition of same parameters, K-LSPSc achieves more accurate local structure constraints than LSPSc, and K-LSPSc has a smaller convergence of average objective value than LSPSc in Fig 9.

**Fig 10:**
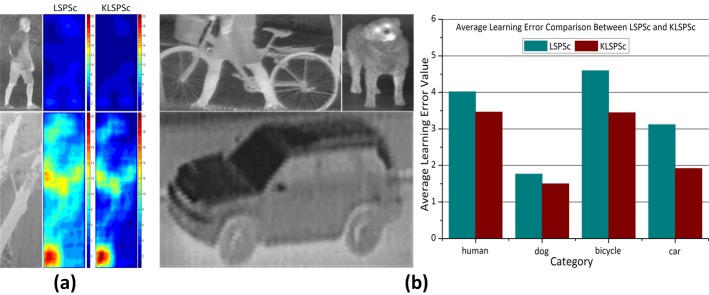
Comparison of the identification ability to infrared target categories between LSPSc and K-LSPSc. (a) Original images and their LSPSc/K-LSPSc matching error based on the *infrared human* dictionary; (b) dictionary generation for different categories and comparison of average LSPSc and K-LSPSc matching errors in each category based on the corresponding dictionary.

To analyze the identification ability of infrared targets between LSPSc and K-LSPSc, we sample subregions from the original image in Fig 10(A), learn the LSPSc/K-LSPSc matching error H_*t*_ by (21) and (22) in each subregion. To facilitate visual comparison, the LSPSc/K-LSPSc matching errors are computed by pixels in the original image. The results are plotted on the right side of Fig 10(A). Based on the human dictionary, the LSPSc/K-LSPSc matching error of pedestrian is small for consistent basic elements and similar structures. Although parts of the basic elements are consistent, the local structures of tree trunk are different from the human body, so the LSPSc/K-LSPSc matching error is large in branch. Therefore, LSPSc/K-LSPSc has the ability to identify infrared target according to the dictionary, and can reduce the influence of noise and fuzzy details in thermal images and natural texture in background. Furthermore, compared with LSPSc, the K-LSPSc matching error is smaller in the areas of human body and homogeneous background, but the relative differences between human and branch is bigger. This is beneficial for K-LSPSc to further reduce the interference of scene and distinguish infrared targets in different categories.

We use the template sets in human, dog, bicycle, and car categories (as instanced in Fig 10(B)) to produce corresponding local sparse structure dictionaries, and count the statistical mean of LSPSc/K-LSPSc matching errors in each category images. The smaller average matching error is, the stronger identification ability of the dictionary to the corresponding object class. First, as a result of the linear method in estimating the spatial local manifold relationship, the target with complex shape (bicycle and human) have a higher average LSPSc matching error, and the target with simple shape (dog and car) have a lower average LSPSc matching error. Second, for targets without subjective postures (bicycle and car), their shape depends only on imaging angles and their K-LSPSc matching error is remarkably lower than that of LSPSc. For targets with subjective postures (human and dog), the advantage of K-LSPSc is weak due to the dynamic shapes (changing local structures) of the targets.

**Fig 11:**
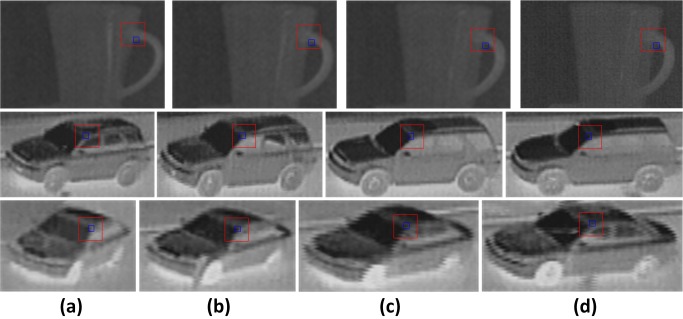
The differences of patch and subregion feature in changing viewing angles.

For rigid targets, the structural change is mainly caused by the change of imaging angles. The template set itself contains targets in large viewing angle changes, and the same physical structure in small viewing angle changes will produce a certain imaging distortion. LSPSc computation of local nonlinear structure is inaccurate, which makes it difficult to match the different error structures before and after the change of the viewing angle. K-LSPSc is more precise because of the nonlinear calculation of the structure. In a certain range of viewing angles, the patch feature and essential structure in the subregion change little, as shown Fig 11, so the K-LSPSc is more robust to viewing angles. In Table 1, the LSPSc and K-LSPSc objective value H_*t*_ and ${\hat{H}}_{t}$ of red subregions in Fig 11(A), 11(B) and 11(D) are calculated using 11(C) as a template. K-LSPSc results smaller objective values.

**Table 1:** Comparison of LSPSc and K-LSPSc in changing viewing angles.

Dictionary	Subregions	LSPSc H_*t*_	K-LSPSc ${\hat{H}}_{t}$
Cup (c)	Cup (a)	0.99	0.87
	Cup (b)	0.95	0.86
	Cup (d)	1.01	0.96
Car1 (c)	Car1 (a)	1.57	1.49
	Car1 (b)	1.49	1.46
	Car1 (d)	1.51	1.47
Car2 (c)	Car2 (a)	1.67	1.58
	Car2 (b)	1.53	1.54
	Car2 (d)	1.62	1.55

**Fig 12:**
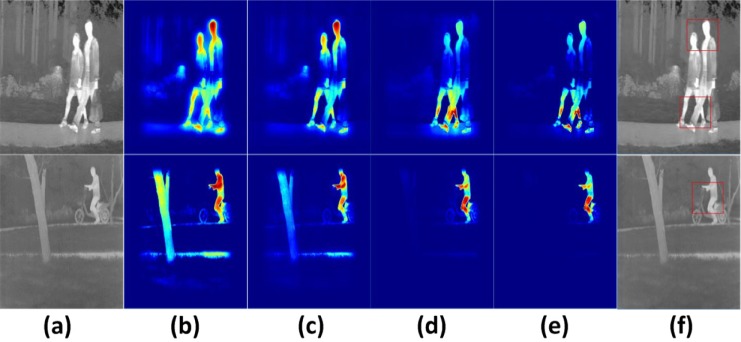
Comparison of target probability extraction by KSc, LSc, LSPSc, and K-LSPSc. (a) the source image; (b~e) the outputs of KSc/LSc/LSPSc/K-LSPSc target probability multiplied with ROIs of original images.

To compare the performance in infrared target recognition by KSc, LSc, LSPSc and K-LSPSc-based LSSM, we replace LSPSc/K-LSPSc with the LSc/KSc algorithm, but keep the rest of the steps in LSSM the same. The extraction of target probability *ρ*_*t*_ is implemented based on the corresponding *infrared human* dictionaries in the ROIs of original images. ROI can filter out homogeneous background, leaving only target areas consistent with the template. The KSc/LSc/LSPSc/K-LSPSc target probability results are shown in Fig 12B–12E. By requiring only robust sparsity, KSc and LSc are confused by pseudo targets, and the false alarm rates are high (stone, road, and branch in (b) (c)) for some similar local features between pseudo targets and template. There are obviously different neighboring structure relations between pseudo targets and template, which results in a small false alarm rate in LSPSc (d). K-LSPSc has a higher identification ability with more significant peaks of target probability and less background interference than LSPSc (e). Red boxes in (f), same size as the subregion, mark the most potential target areas by the target probability image.

## Results and discussion

To demonstrate the performance of LSSM by using the simple template set, the contrastive analysis of different sparse methods [52] based on a single template and multiple templates is provided. Besides, LSSM is compared with other general target detection method [34,35], which uses a robust LARK feature to implement entire matching with a single template, and has good capability to detect human faces and targets with simple shape and compact structure.

In this section, experiments are executed on three data sets: *Caltech 256*, *Infrared Car and Bicycle* (captured by FILR Tau 2–640 Uncooled LWIR Thermal Imaging Camera, including total 155 images with 95 images containing bicycle and 60 images not containing bicycle, 87 images containing car and 68 images not containing car.), and *Infrared Human* dataset. For fair comparison, we use the approach in [52] to label bounding boxes around interested targets. If the detected region by our methods lies on the ground truth, we evaluate it as a correct detection and a false positive otherwise. The receiver operating characteristic (ROC) and the Recall versus 1-Precision curves [52] are employed to measure performance.

### Parameter setting

In LSPSc/K-LSPSc formulation there are three free variables: the dictionary size, the weight of sparse term *λ*, and the weight of local manifold preserving term *β*. Some works [20,50] have been done to show the relationship from the dictionary size and sparsity to image classification accuracy. We employ the *Caltech 256* and *Infrared* datasets to study the dependence of the target recognition accuracy on three important parameters.

**Fig 13:**
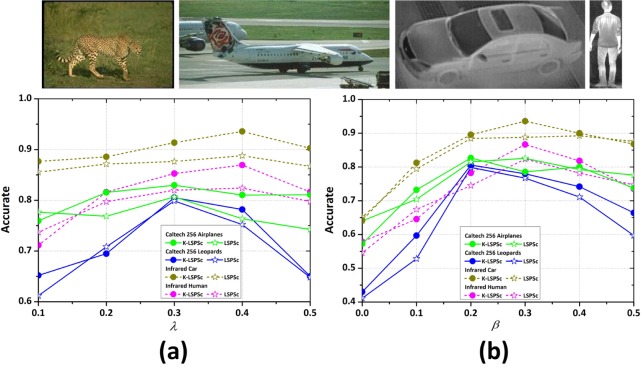
The effect of the weight of the sparse term *λ* and the weight of the structure term *β* on target recognition accuracy in the *Caltech 256 Leopards*, *Airplanes*, *Infrared Car*, and *Infrared Human* datasets by LSSM.

We build two mixed datasets from the *Caltech 256* dataset (http://www.vision.caltech.edu/Image_Datasets/Caltech256/), which contains 256 categories. One is the *Airplanes* dataset, consisting of 750 positive and 750 negative images. The 750 positive images are from the *Airplanes* category (800 images, 50 of them used to learn the *airplanes* dictionary), whereas the 750 negative images are randomly sampled from the rest of the categories in *Caltech 256*. The other dataset is the *Leopards* dataset, consisting of 160 positive and 160 negative images. The 160 positive images are from the *Leopards* category (190 images, 30 of them used to learn the *leopard* dictionary), whereas the 160 negative images are randomly sampled from the rest of the categories in *Caltech 256*. Based on the Sc/LSPSc/K-LSPSc dictionaries of *airplanes*, *leopard*, *infrared car* and *infrared human*, we apply the Sc-, LSPSc- and K-LSPSc-based LSSM on the four datasets, and count the corresponding recognition accuracy, which is defined as *Acc* = (*TP* + *TN*)/(*nP* + *nN*), where *nP* is the total number of positive, and *nN* is the total number of negative. Table 2 and Fig 13 show the results.

**Table 2:** The effects of dictionary size on Sc, LSPSc, and K-LSPSc on datasets by LSSM.

Dataset	Dictionary	LSSM methods	Dictionary Size		
			256	512	1024
*Caltech 256 Leopards*	*leopard*	Sc	58.48	60.56	**61.27**
		LSPSc	70.08	76.01	**79.84**
		K-LSPSc	75.89	77.18	**80.52**
*Caltech 256 Airplanes*	*airplanes*	Sc	62.17	61.41	**66.33**
		LSPSc	78.16	76.69	**80.54**
		K-LSPSc	78.82	**82.90**	81.93
*Infrared Car and Bicycle*	*infrared car*	Sc	75.12	**76.24**	74.57
		LSPSc	**88.79**	88.54	87.12
		K-LSPSc	**93.56**	93.47	91.03
*Infrared Human*	*infrared human*	Sc	63.16	**63.48**	62.65
		LSPSc	81.48	**82.39**	81.91
		K-LSPSc	**86.94**	85.18	85.27

Intuitively, if the dictionary size is too small, LSSM may lose discriminant ability; if the dictionary size is too large, LSSM will be time consuming. As shown in Table 2, for the *Caltech 256 Leopards* dataset, in which the visible targets have rich texture details, the performance of Sc/LSPSc/K-LSPSc increases as the dictionary size goes up to 1024. For the *Infrared Car* and *Infrared Human* datasets, in which the infrared targets have simple structures and blurred textures, the performance of Sc/LSPSc/K-LSPSc peaks when the dictionary size is 256 or 512. In addition, static targets get higher recognition accuracy than dynamic targets (*Airplanes* versus *Leopards*, *Infrared Car* versus *Infrared Human*). Infrared targets achieve higher accuracy than visible objects for the relatively less textures and simple structures (*Infrared Human* versus *Leopards*, *Infrared Car* versus *Airplanes*). It can be seen that for the targets with complex and changing structures, the dictionary size should be large (*Airplanes* and *Leopards*). However, for targets with simple structures, performance has no evident improvement as the dictionary size grows. Therefore, in experiments the dictionary size is set as 1024 for visible dynamic targets and 256 for visible static and infrared targets, to reduce the computed amount.

The weight of sparse term *λ* enforces the sparsity of the solution. The weight of local structure preserving term *β* constrains the locality of targets. Fig 13 shows that *λ* has little effect on recognition accuracy, but the accuracy is impacted greatly by *β*. Accuracy increases initially and then decreases as *β* grows further. Overall, LSPSc/K-LSPSc achieve good performance when *λ* values are 0.3~0.4 and *β* values are 0.2~0.3. In visible images, excessive structure constrain makes the solution difficult to converge, thus causing the reduction in accuracy. In infrared images, the details are fuzzy and local structures are simple relatively, so appropriate enhancement of the structure constraint can improve accuracy. Thus, for the *Caltech 256* dataset, *λ* = 0.3 and *β* = 0.2, and for the *Infrared* dataset, *λ* = 0.4 and *β* = 0.3.

### Infrared vehicle detection

**Fig 14:**
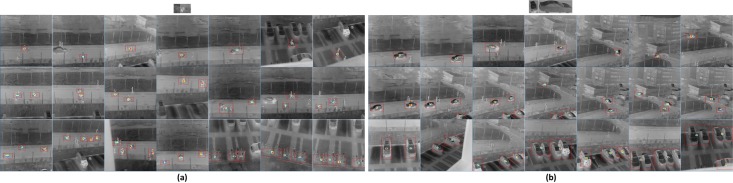
Recognition results of infrared vehicles (bicycle/car) in different scales, rotations, and imaging angles by LSPSc-based LSSM. The red boxes are drawn at the target locations.

In the *Infrared Car and Bicycle* dataset, the images with size 640*480 have little scene changes. Two experiments are conducted with the dataset. One involves 155 images for bicycle and car detection, which contain bicycles and cars from side to front view at various sizes, with a ratio of bicycle size about 2 and a ratio of car size about 3. We use a simple bicycle template set to detect bicycles from 95 positives and 60 negatives, as shown in Fig 14(A). A simple car template set is used to detect cars from 87 positives and 68 negatives, as shown in Fig 14(B). In addition, as explained in Section 3, we construct an expanded template set to deal with the cases of multi-scale, multi-rotation, and different imaging angles of infrared targets. We fix *λ* = 0.4, *β* = 0.3, and the dictionary size at 256. Fig 14 shows the original simple template set of bicycle and car, and outputs of LSPSc-based LSSM for bicycle and car detection. It shows that different vehicle types can be detected for most of the consistent local structures from test targets to the template. Due to the expanded template, targets in different scales, rotations, and imaging angles are recognized accurately.

**Fig 15:**
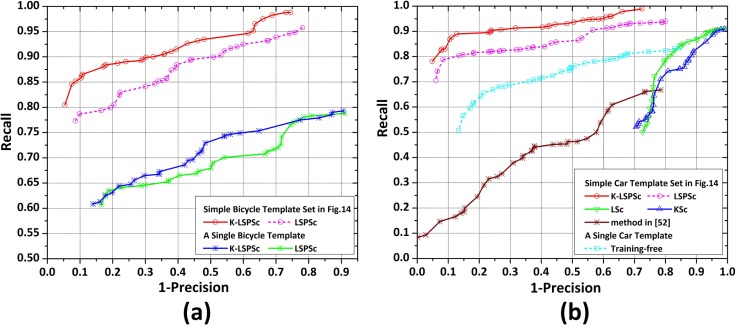
The performance analysis of LSSM in vehicle detection. (a) Precision-recall (recall versus 1-precision) curves of bicycle detection by LSPSc/K-LSPSc-based LSSM models using two bicycle templates. (b) Precision-recall curves of car detection by the training-free method using a single car template, method in [52], and LSc/KSc/LSPSc/K-LSPSc-based LSSM methods using a simple car template set.

By computing recall and precision at parameters variations in methods (dictionary size, weights of sparse and local manifold preserving, overall threshold, and confidence level), we conduct two experiments to show the overall performance of LSSM on different templates, and compare it against other methods. Fig 15(A) plots the performance of LSPSc/K-LSPSc-based LSSM in bicycle detection, using the simple bicycle template set in Fig 14(A) and a single template of the side view of electric bicycle in the template set. Here, 1-Precision is indeed 1.0-Precision, a low 1-Precision is desirable since precision should be high, and that the ideal system will have points in the top left of the ROC curve. We can clearly see that the performance is obviously improved by a choice of the template images with different imaging angles, and it is quite consistent with the previous observations that K-LSPSc-based LSSM outperforms LSPSc-based LSSM on both templates, although the advantage is not remarkable by using a single template.

Essentially, the proposed model is a local template matching method based on sparse learning with very few template images. Thus, we not only measure among the LSc/KSc/LSPSc/K-LSPSc-based LSSM models and the part-based sparse representation method [52] by using the simple car template set in Fig 14(B), but also compare LSSM against the training-free method [34,35] by using a single template of the side view of car. Fig 15(B) shows the results of car detection. The method in [52] distinguishes by the similarity between the test samples and “part vocabulary” (constructed by objects of the target class). Because of the imaging characteristics (noise and fuzzy details) in infrared images and use of the simple template set, its similarity discrimination between different car types has low fault-tolerance and adaptability. The training-free method in [34,35] loses the targets in changing imaging angles due to the single template. LSc and KSc mostly pay attention to sparse representation of the test sample, the local features consistent with the template set are detected as a target, so the false positives are high but the false negatives are relatively low. The LSPSc/K-LSPSc-based LSSM keep the consistent local features and sparse structures in neighborhood synchronously, so they have outstanding performance.

### Infrared human recognition

The *Infrared Human* dataset is chosen for human recognition. To be more general to test environments, it is composed of 224 images captured by two thermal cameras in different scenes with resolution ratio of 308*239, 558*419 and 617*506.

#### Multi-scale and multi-rotation test

**Fig 16:**
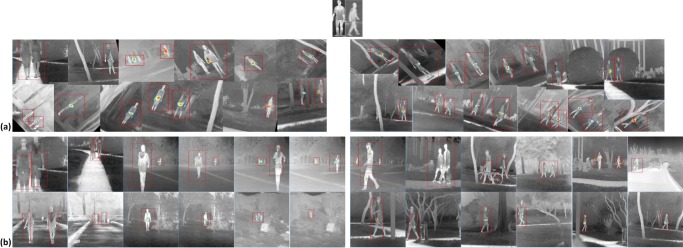
The original simple human template set and results of LSPSc-based LSSM in standing and walking human recognition in large distinctions of scale, rotation, and imaging angle. (a) multi-rotation; (b) multi-scale. The red boxes are drawn at the target locations.

In the vehicle detection we showed the performance of LSSM in the presence of quite different imaging angles but with a moderate scale variation (a size ratio of about 3). In this part, we further evaluate LSSM on a more general scenario for human recognition, where the scale ratio is over 10, various rotations are executed on part images from −50°∼50°, and a large difference of target imaging angles exists. We construct a simple human template set consisting of standing and walking man, to consider both the posture and motion changes of humans. It is expanded in the same manner as the vehicle template set to mainly detect standing and walking humans with slight gesture variances but diverse sizes and rotations. Fig 16 shows the original simple human template set and outputs of LSPSc-based LSSM. Because dictionary learning has programmed the mirroring, scaling and rotation of template images, coupled with a certain ability of anti-interference from background (Fig 10) and fault-tolerant of targets (Fig 12) in LSPSc, LSSM is capable of recognizing and localizing for infrared standing and walking humans based on the few template images as far as possible in Fig 16.

#### General human recognition

**Fig 17:**
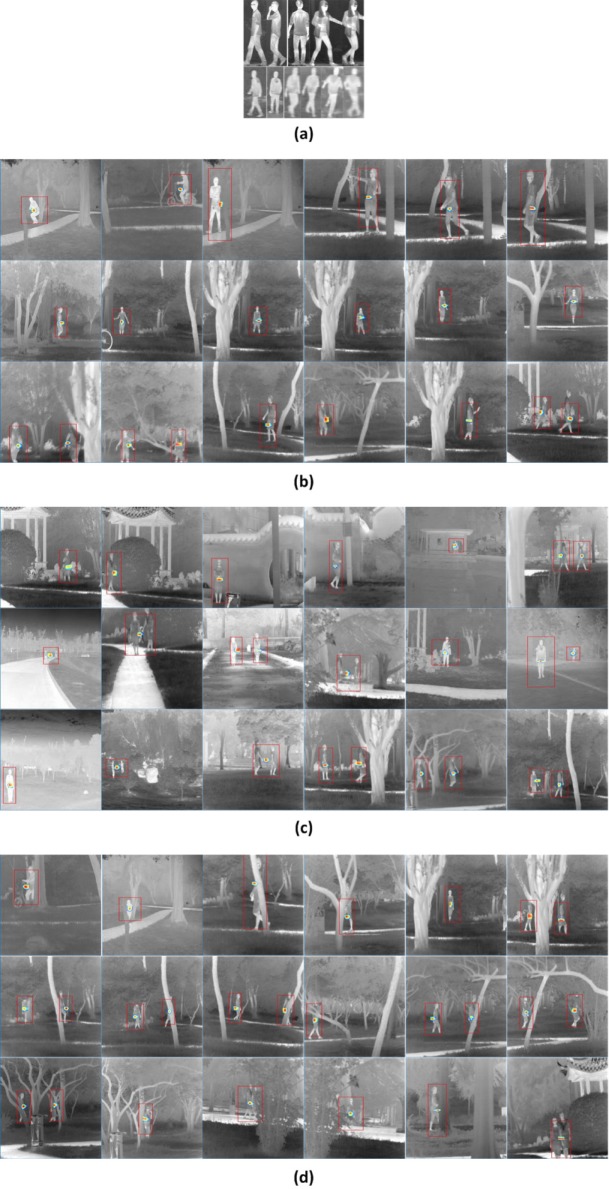
The robust general infrared human recognition by K-LSPSc based LSSM. The red boxes are drawn at target locations. (a) Simple template set of human; (b) Robust human recognition with posture variations; (c) Robust human recognition with scene changes; (d) Robust human recognition with occlusions.

Our model is further applied into more complicated scenarios, where we consider robust human recognition in condition of posture variations (Fig 17(B)), scene changes (Fig 17(C)), and occlusion occurrences (Fig 17(D)).To improve the robustness of LSSM, we use the template set in Fig 17(A). It contains more postures and the expansion includes sufficient local features and structures in infrared human body, so the corresponding learned *infrared human* dictionary is relatively robust. Because of the enhanced complication of structures in template and test images, we employ K-LSPSc to further improve the abilities of anti-interference and fault-tolerance in LSSM for identification. Fig 17 shows that the K-LSPSc-based LSSM can recognize and localize the infrared human body reliably in complex environments.

It should be emphasized that, because the *Infrared Human* dataset has targets of standing and walking bodies in different imaging angles and behavior patterns basically, the template set picks several images of standing and walking bodies in representative imaging angles and postures. Moreover, to improve the stability of the dictionary, template images are captured by disparate cameras with different image qualities. If the postures of interested or to be detected humans are abundant, more images of typical motions can be added into the template set further.

**Fig 18:**
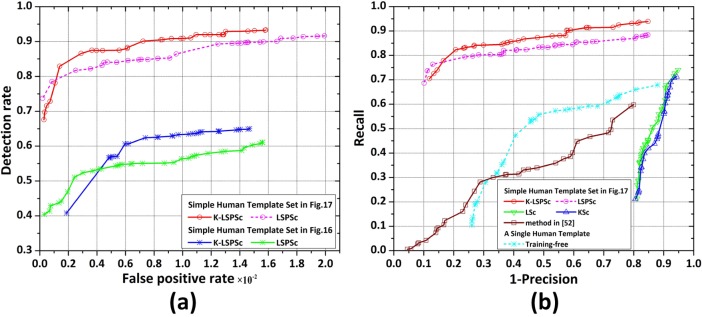
The performance analysis of LSSM in human detection. (a) ROC curves of LSPSc/K-LSPSc-based LSSM using two human templates; (b) Precision-recall curves of the training-free method using a single human template, method in [52], KSc/LSc/LSPSc/K-LSPSc-based LSSM models using the simple human template set in Fig 17(A). They are recognitions of human body in the *Infrared Human* dataset.

Fig 18 shows the ROC curves of LSPSc/K-LSPSc-based LSSM with respect to two different human template sets (template sets in Fig 16 and Fig 17) and recall versus 1-precision curves corresponding to methods in Fig 15(B) (single template is the side view of walking human in Fig 16). It can be seen from Fig 18(A) that the performance of the proposed model is obviously affected by the choice of template sets with different comprehensive degrees of local sparse structures. More specifically, in the range of low false positive rates LSPSc provides a higher detection rate, and this phenomenon is stable in the recall versus 1-precision curves in Fig 18(B).

As seen in Fig 18(B), the performance of LSSM in the human dataset is consistent with the results in the vehicle dataset, which detects better than other sparse coding-based methods and the training-free method. As long as the template set contains sufficient local sparse structures in different shapes of the target class, the corresponding LSSM models have stronger robustness for imaging characters, scene variations, shape changes, and partial occlusions of targets. Although the training-free method can recognize human action and the matching process has a certain fault-tolerance, the accuracy using a single template is low in presence of diverse shapes, imaging angles, and occlusions.

### Computation time analysis

Table 3 gives the comparison of computational efficiency on two infrared datasets by different algorithms. It can be seen that LSSM is more time consuming, in particular the KLSPSc-LSSM model. Although the kernel nonlinear processing improves the recognition performance for natural data, it reduces the efficiency of the model. On the other aspect, LSSM training time is obviously higher than training-free method and method in [52]. However, training is usually carried out only once and offline, computational efficiency is more important when testing in real-time implementations, so LSSM has more advantages in the test efficiency and performance.

**Table 3:** The comparison of calculational efficiency by different algorithms.

Dataset	Testing time (s)			
	Training-free method	Method in [52]	LSPSc-LSSM	KLSPSc-LSSM
*Infrared Car and Bicycle*	9.2411	15.5295	13.8028	30.9442
*Infrared Human*	13.4669	22.5344	20.0323	44.6790
	Training time (s)			
*Infrared Car and Bicycle*	0	0.0747	1.5476	2.4638
*Infrared Human*	0	0.5930	26.9531	49.2764

## Conclusions

By adding a spatial local manifold constraint into the classical sparse coding algorithm, we propose the LSPSc and K-LSPSc formulation in this paper, which can simultaneously preserve both the sparsity of patches and the intrinsic structure of subregions. Moreover, we analyze the relationships between LSPSc/K-LSPSc, Sc, KSc, LSc and structured sparse coding. Experiments show that the local sparse structure quantization by LSPSc/K-LSPSc can alleviate the background interference and improve the stability of sparse representation of infrared targets. We further present an LSSM approach to realize the robust general target detection by using a simple template set. Without plenty of template images, the simple template set of a target class needs only several images containing relatively comprehensive local structures to learn a sufficient sparse structure dictionary.

In the future, our work need to be extended in the following directions: 1) Feature selection: Currently, we use the original gray information in images. How to employ more intrinsic features to improve the discrimination for LSPSc is badly needed. 2) Nonlinear structure constraint: We use a linear manifold to mine the spatial local manifold among samples and embed the local manifold relationships into their sparse quantization. To be appropriate for nonlinear data, LSPSc is extended into kernel space, which entails large computation. We will directly consider a nonlinear manifold method in our future work, such as LTSA, ISOMAP, and so on.

## Supporting information

S1 File The source infrared image datasets used in the paper. (ZIP)
