# Supplementary figures and images for: Local structure preserving sparse coding for infrared target recognition (part 2 of 2)
Source: PLoS One. 2017 Mar 21;12(3):e0173613. doi: 10.1371/journal.pone.0173613 (PMC5360252; doi:10.1371/journal.pone.0173613)

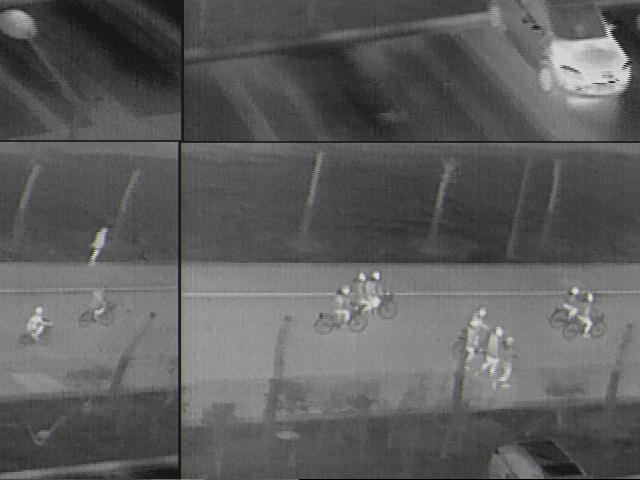

Supplement: S1 File — (ZIP) [file pone.0173613.s001.zip › infrared car and bicycle set/V34467.bmp]

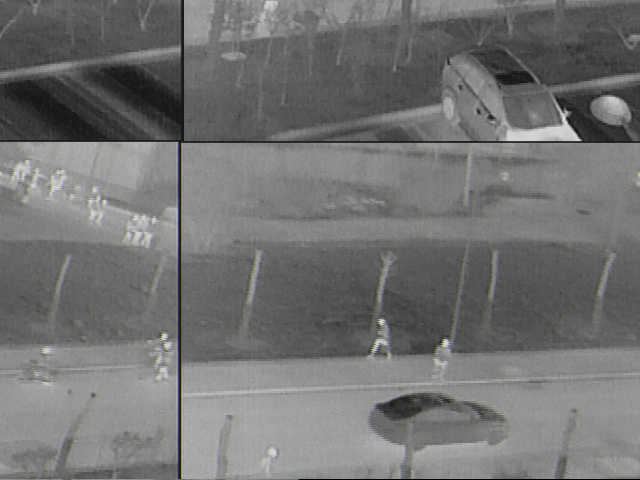

Supplement: S1 File — (ZIP) [file pone.0173613.s001.zip › infrared car and bicycle set/V34497.bmp]

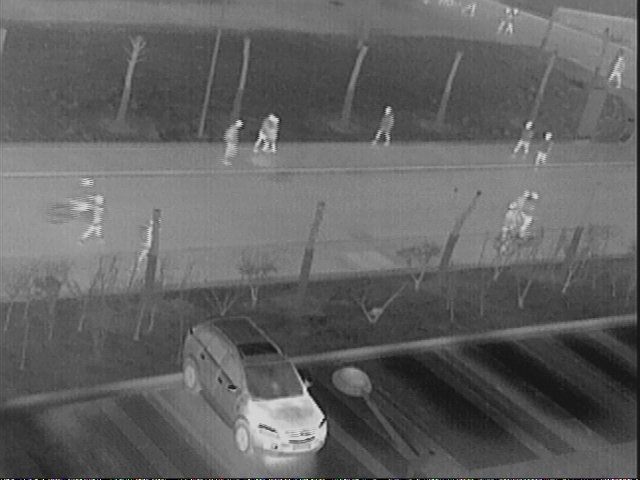

Supplement: S1 File — (ZIP) [file pone.0173613.s001.zip › infrared car and bicycle set/V34534.bmp]

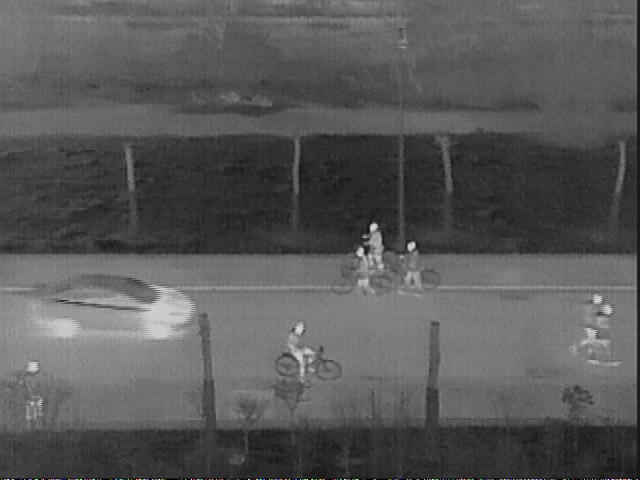

Supplement: S1 File — (ZIP) [file pone.0173613.s001.zip › infrared car and bicycle set/V34546.bmp]

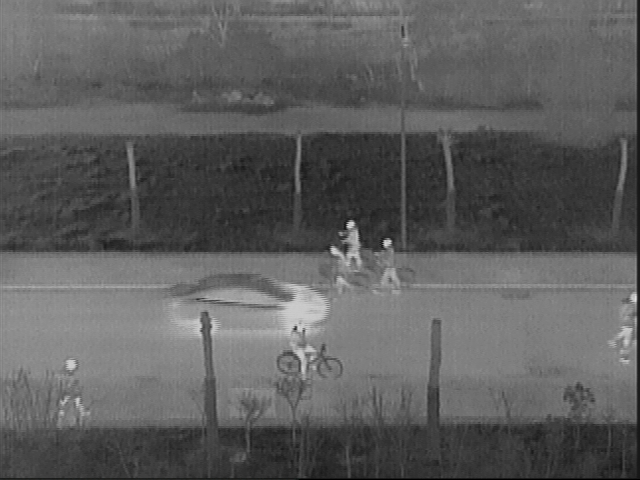

Supplement: S1 File — (ZIP) [file pone.0173613.s001.zip › infrared car and bicycle set/V34547.bmp]

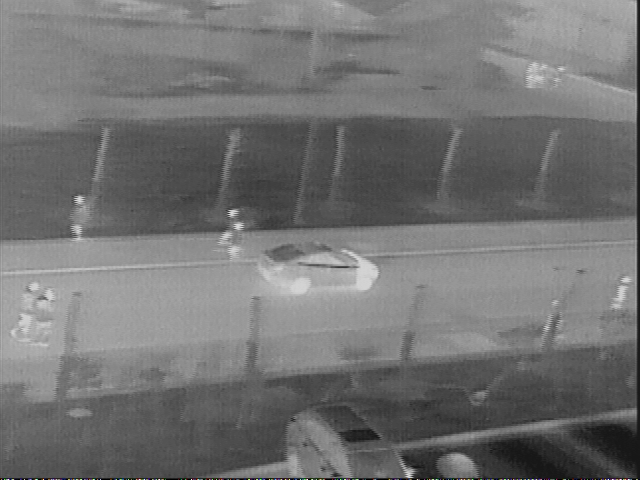

Supplement: S1 File — (ZIP) [file pone.0173613.s001.zip › infrared car and bicycle set/V34554.bmp]

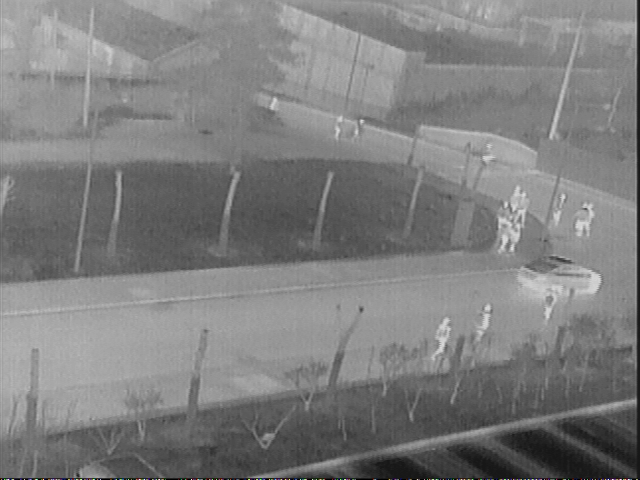

Supplement: S1 File — (ZIP) [file pone.0173613.s001.zip › infrared car and bicycle set/V34560.bmp]

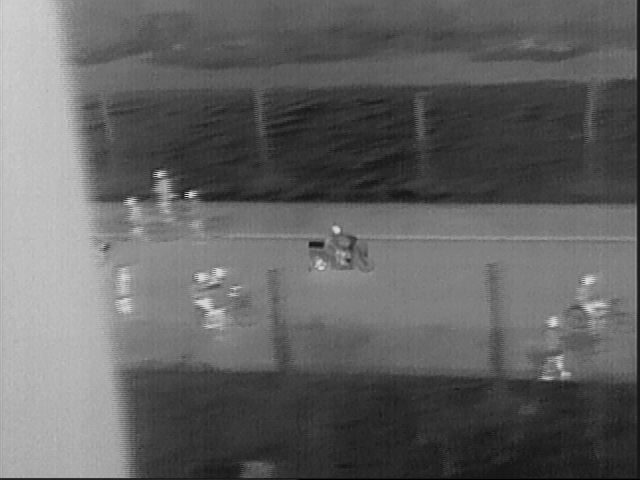

Supplement: S1 File — (ZIP) [file pone.0173613.s001.zip › infrared car and bicycle set/V34567.bmp]

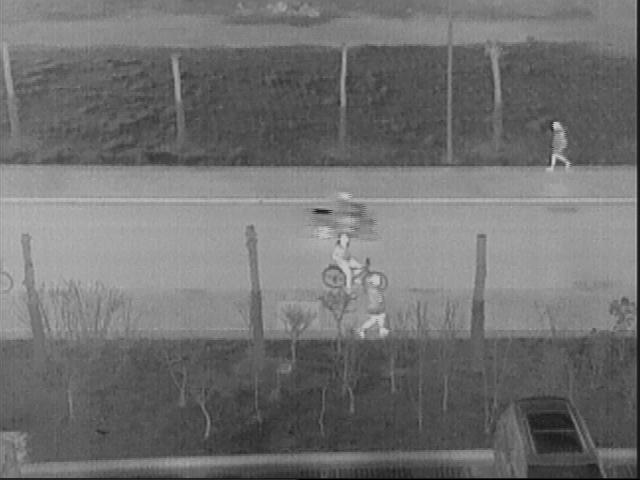

Supplement: S1 File — (ZIP) [file pone.0173613.s001.zip › infrared car and bicycle set/V34569.bmp]

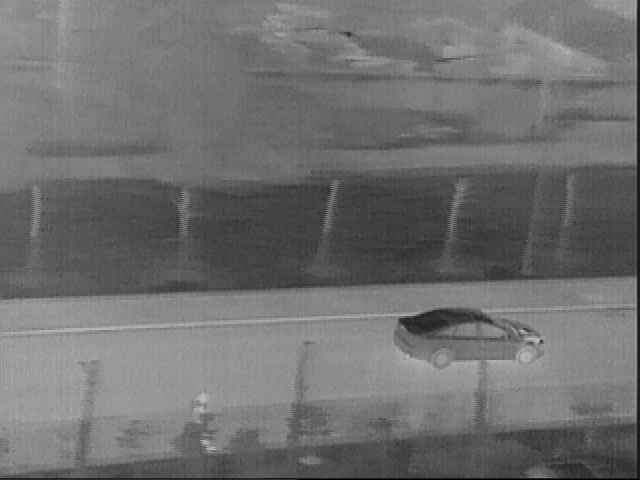

Supplement: S1 File — (ZIP) [file pone.0173613.s001.zip › infrared car and bicycle set/V34583.bmp]

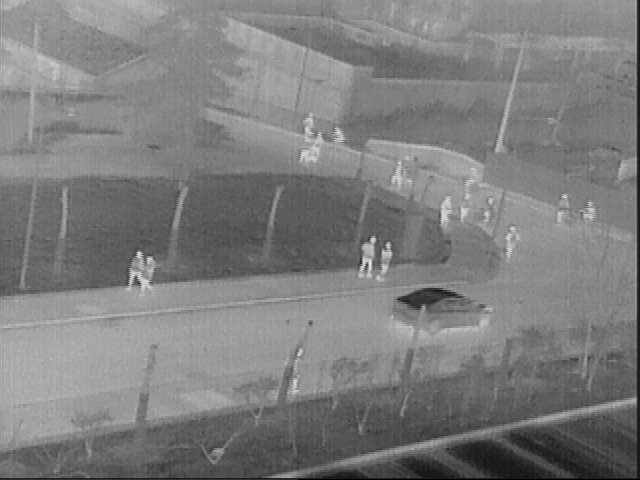

Supplement: S1 File — (ZIP) [file pone.0173613.s001.zip › infrared car and bicycle set/V34589.bmp]

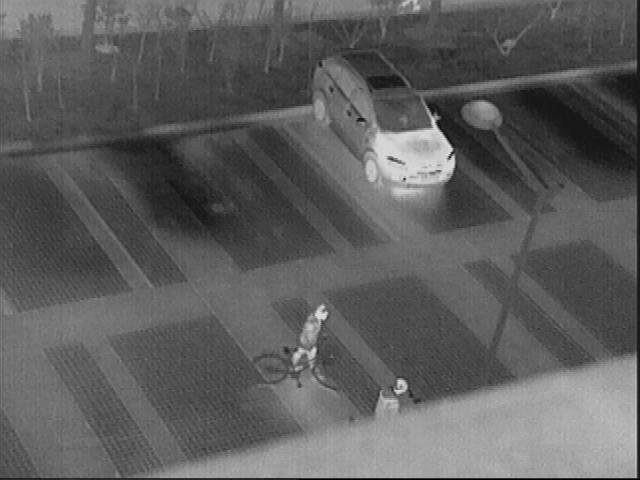

Supplement: S1 File — (ZIP) [file pone.0173613.s001.zip › infrared car and bicycle set/V34599.bmp]

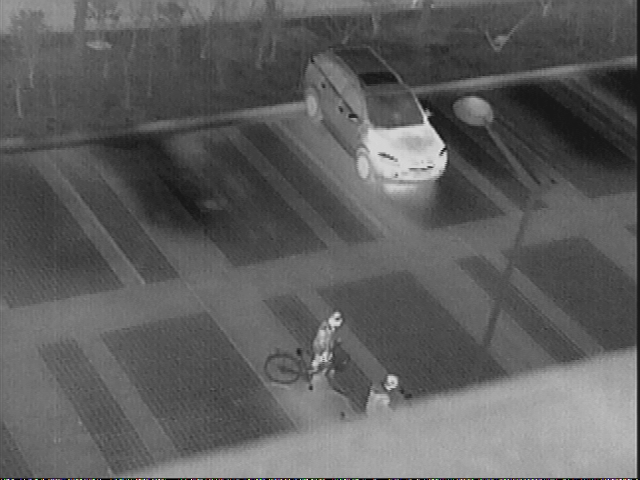

Supplement: S1 File — (ZIP) [file pone.0173613.s001.zip › infrared car and bicycle set/V34600.bmp]

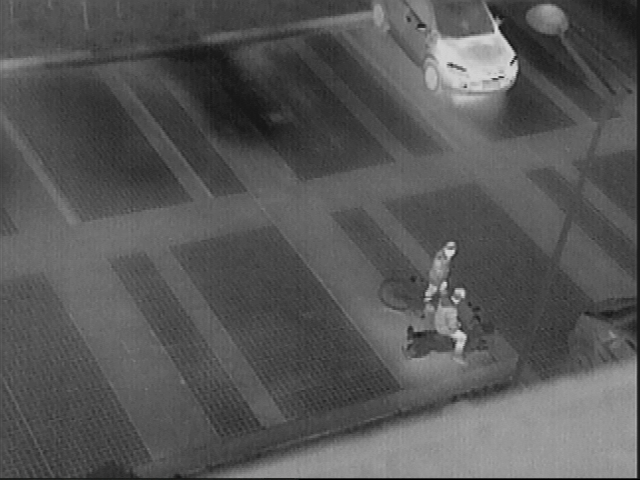

Supplement: S1 File — (ZIP) [file pone.0173613.s001.zip › infrared car and bicycle set/V34603.bmp]

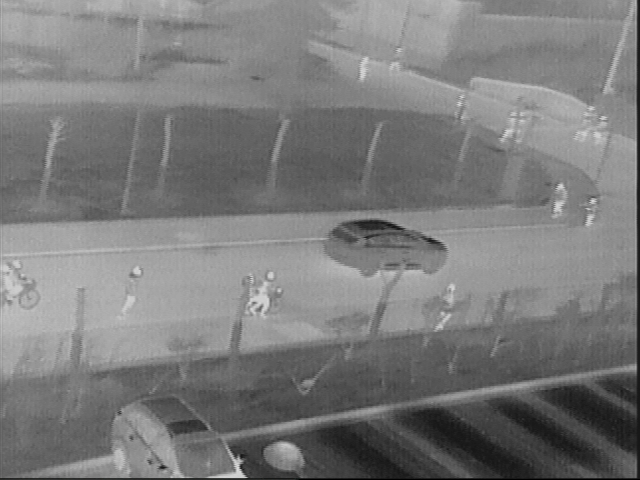

Supplement: S1 File — (ZIP) [file pone.0173613.s001.zip › infrared car and bicycle set/V34611.bmp]

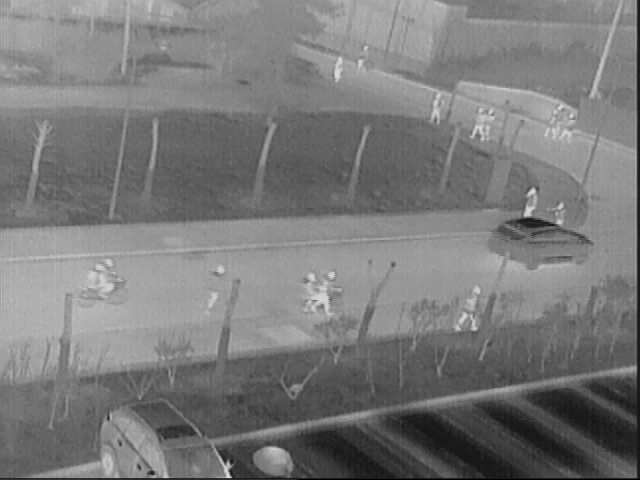

Supplement: S1 File — (ZIP) [file pone.0173613.s001.zip › infrared car and bicycle set/V34613.bmp]

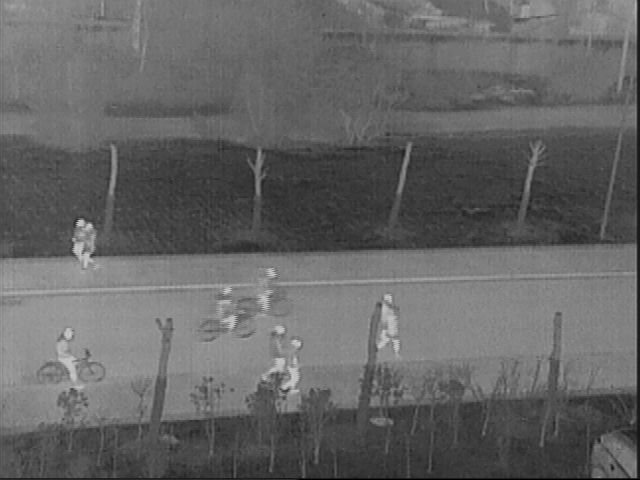

Supplement: S1 File — (ZIP) [file pone.0173613.s001.zip › infrared car and bicycle set/V34621.bmp]

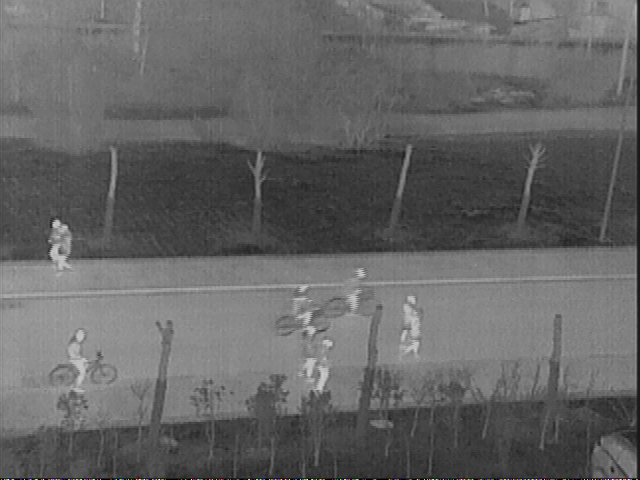

Supplement: S1 File — (ZIP) [file pone.0173613.s001.zip › infrared car and bicycle set/V34622.bmp]

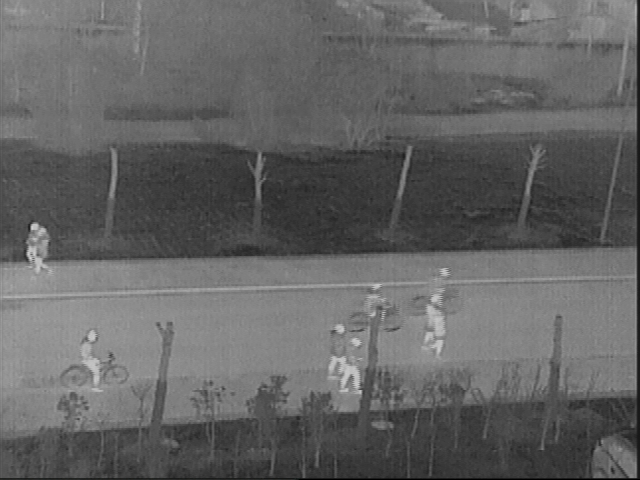

Supplement: S1 File — (ZIP) [file pone.0173613.s001.zip › infrared car and bicycle set/V34623.bmp]

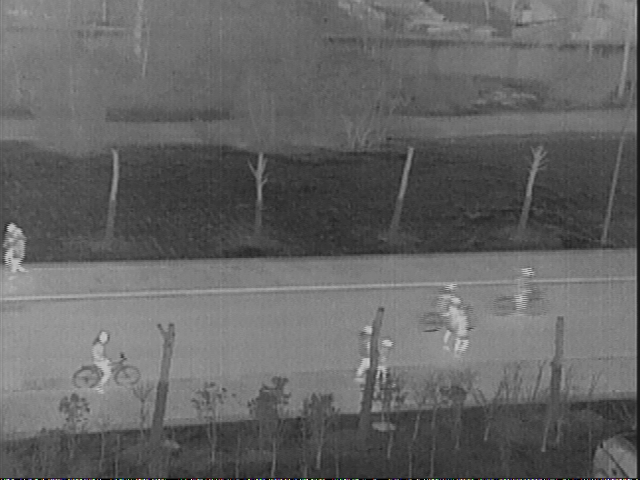

Supplement: S1 File — (ZIP) [file pone.0173613.s001.zip › infrared car and bicycle set/V34624.bmp]

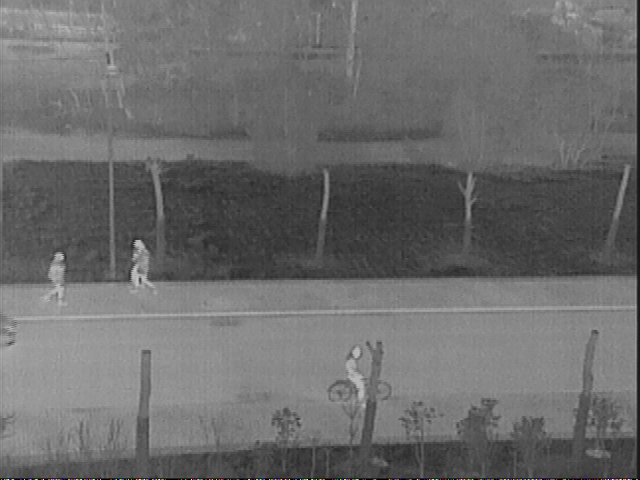

Supplement: S1 File — (ZIP) [file pone.0173613.s001.zip › infrared car and bicycle set/V34628.bmp]

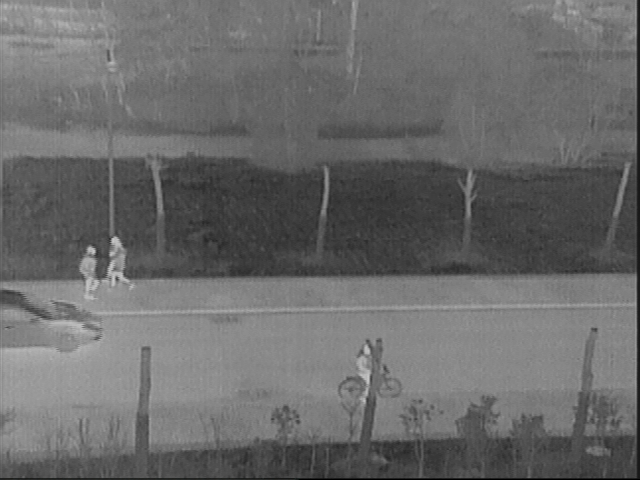

Supplement: S1 File — (ZIP) [file pone.0173613.s001.zip › infrared car and bicycle set/V34629.bmp]

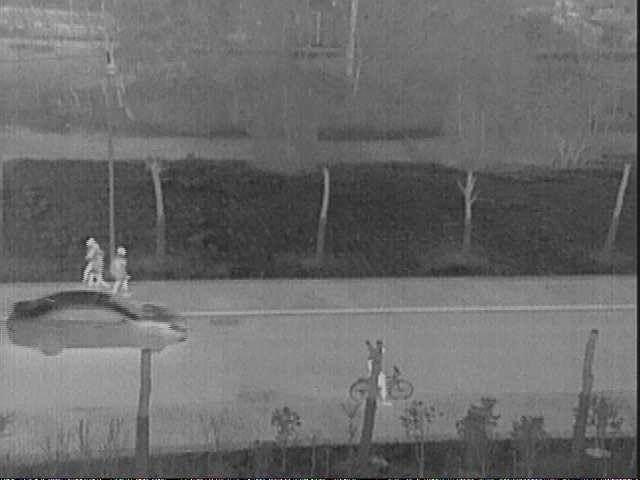

Supplement: S1 File — (ZIP) [file pone.0173613.s001.zip › infrared car and bicycle set/V34630.bmp]

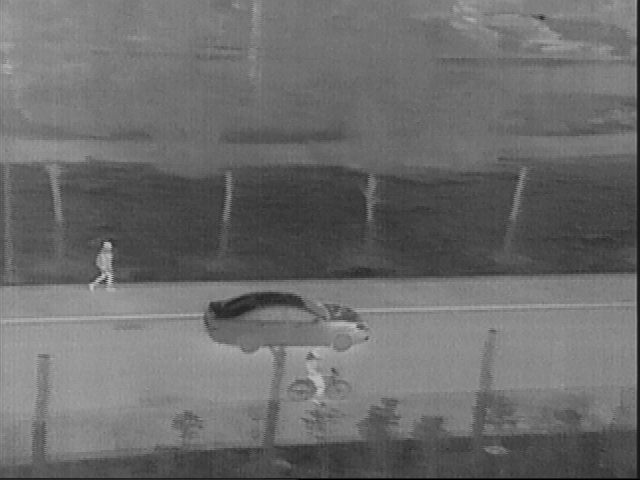

Supplement: S1 File — (ZIP) [file pone.0173613.s001.zip › infrared car and bicycle set/V34633.bmp]

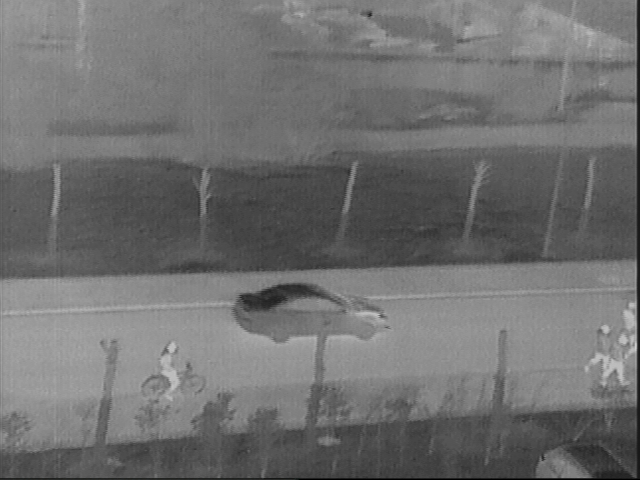

Supplement: S1 File — (ZIP) [file pone.0173613.s001.zip › infrared car and bicycle set/V34635.bmp]

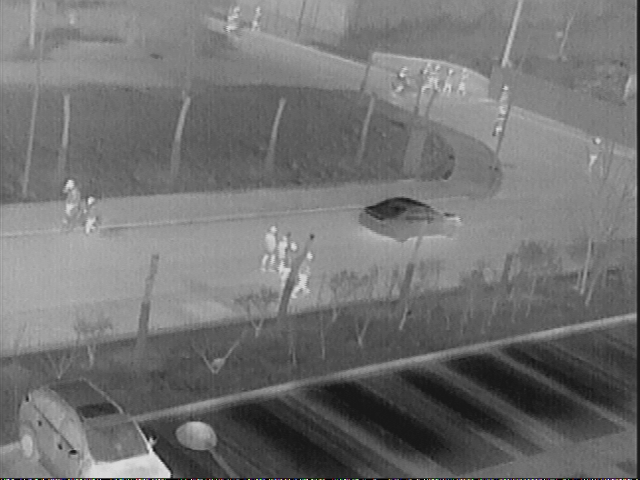

Supplement: S1 File — (ZIP) [file pone.0173613.s001.zip › infrared car and bicycle set/V34644.bmp]

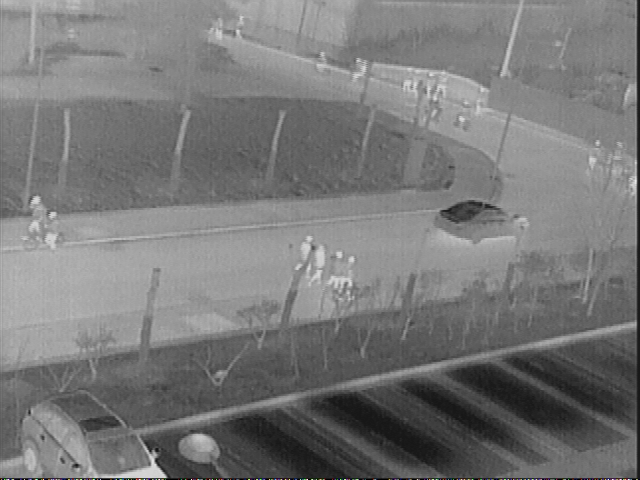

Supplement: S1 File — (ZIP) [file pone.0173613.s001.zip › infrared car and bicycle set/V34646.bmp]

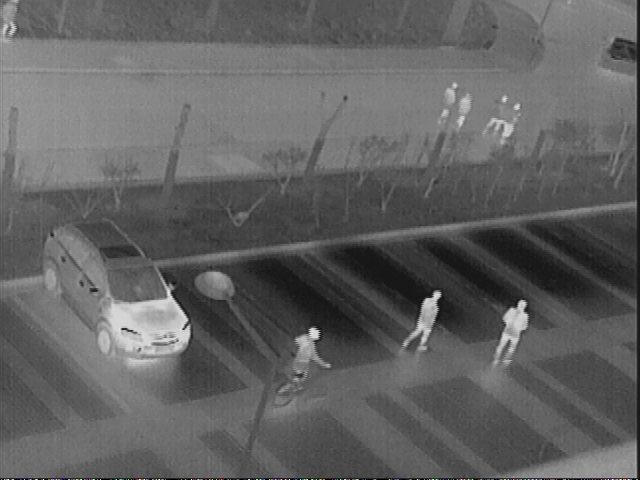

Supplement: S1 File — (ZIP) [file pone.0173613.s001.zip › infrared car and bicycle set/V34654.bmp]

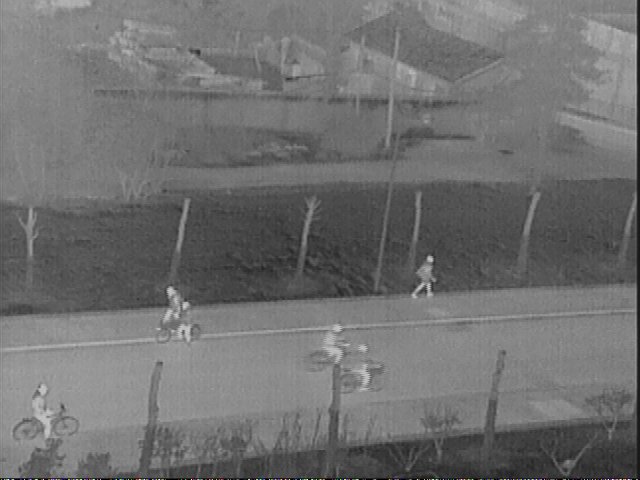

Supplement: S1 File — (ZIP) [file pone.0173613.s001.zip › infrared car and bicycle set/V34658.bmp]

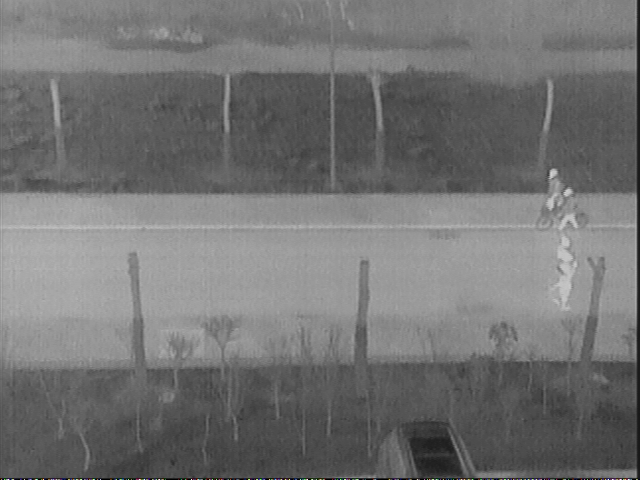

Supplement: S1 File — (ZIP) [file pone.0173613.s001.zip › infrared car and bicycle set/V34672.bmp]

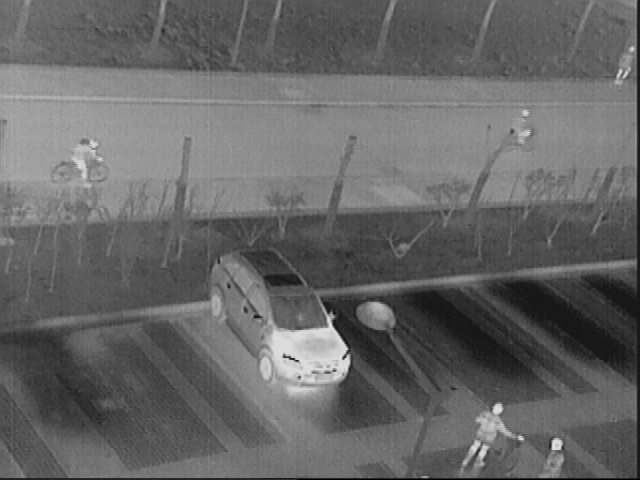

Supplement: S1 File — (ZIP) [file pone.0173613.s001.zip › infrared car and bicycle set/V34677.bmp]

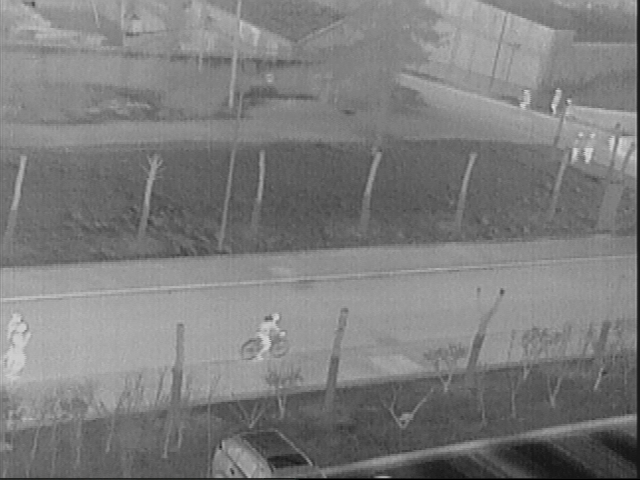

Supplement: S1 File — (ZIP) [file pone.0173613.s001.zip › infrared car and bicycle set/V34685.bmp]

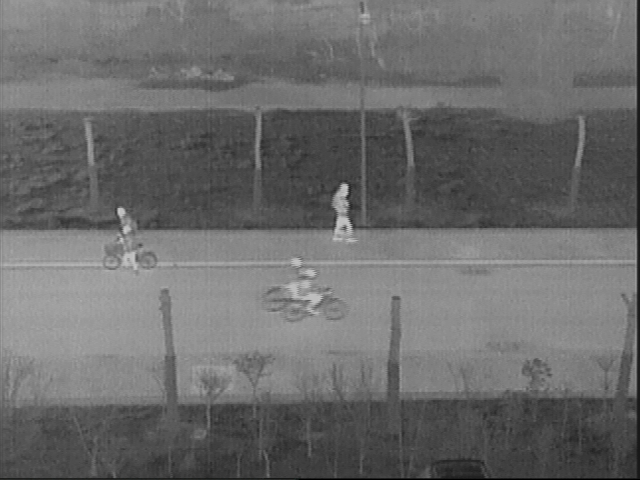

Supplement: S1 File — (ZIP) [file pone.0173613.s001.zip › infrared car and bicycle set/V34693.bmp]

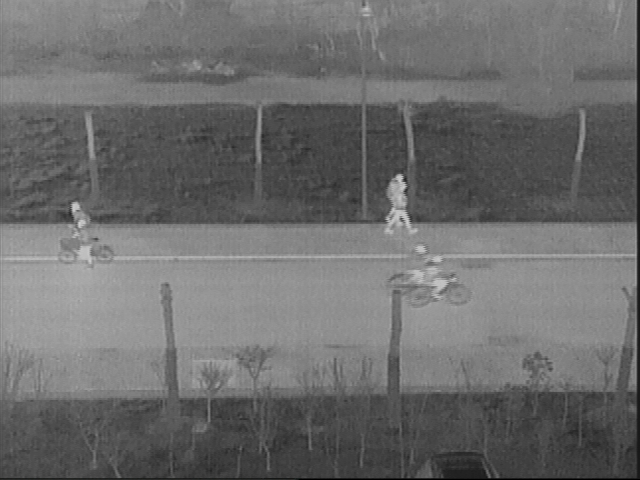

Supplement: S1 File — (ZIP) [file pone.0173613.s001.zip › infrared car and bicycle set/V34695.bmp]

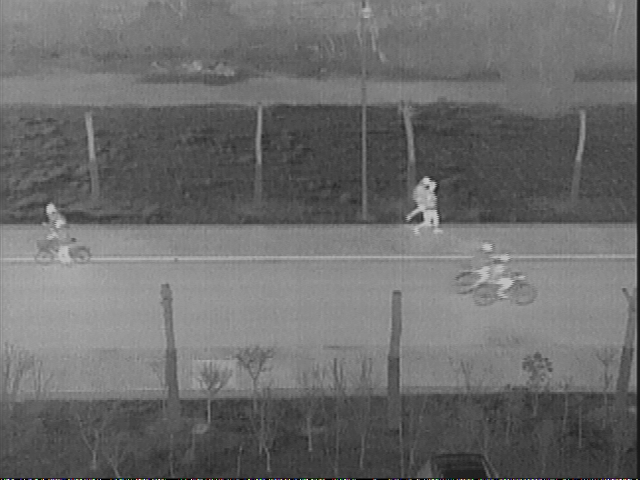

Supplement: S1 File — (ZIP) [file pone.0173613.s001.zip › infrared car and bicycle set/V34696.bmp]

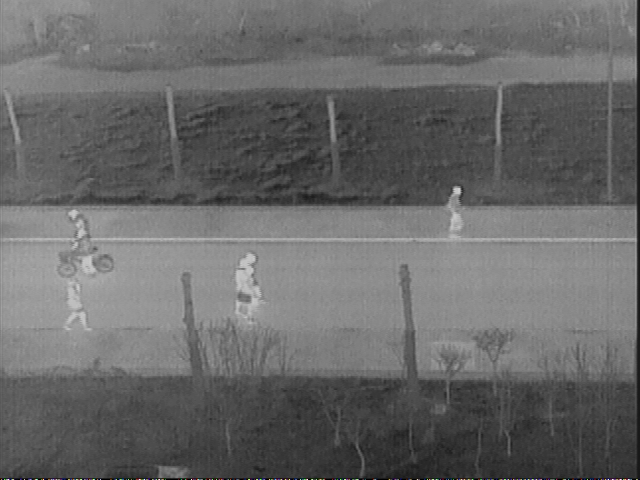

Supplement: S1 File — (ZIP) [file pone.0173613.s001.zip › infrared car and bicycle set/V34706.bmp]

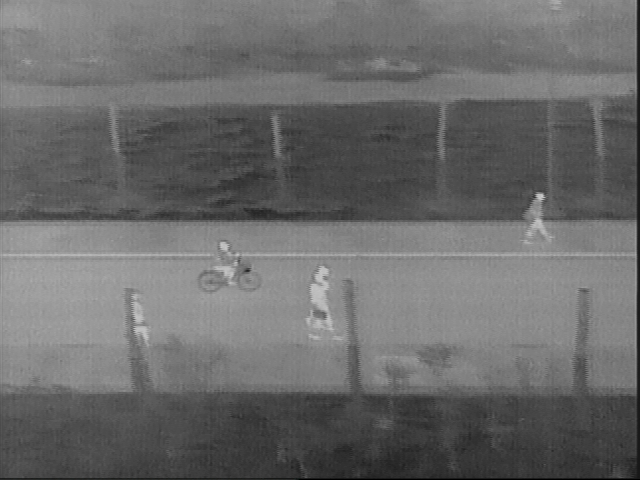

Supplement: S1 File — (ZIP) [file pone.0173613.s001.zip › infrared car and bicycle set/V34711.bmp]

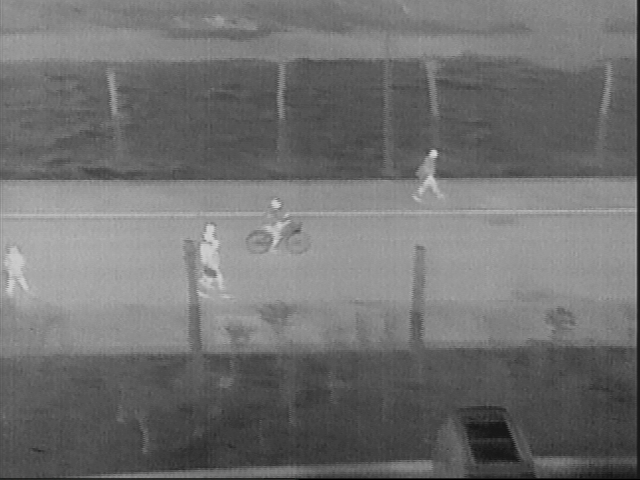

Supplement: S1 File — (ZIP) [file pone.0173613.s001.zip › infrared car and bicycle set/V34713.bmp]

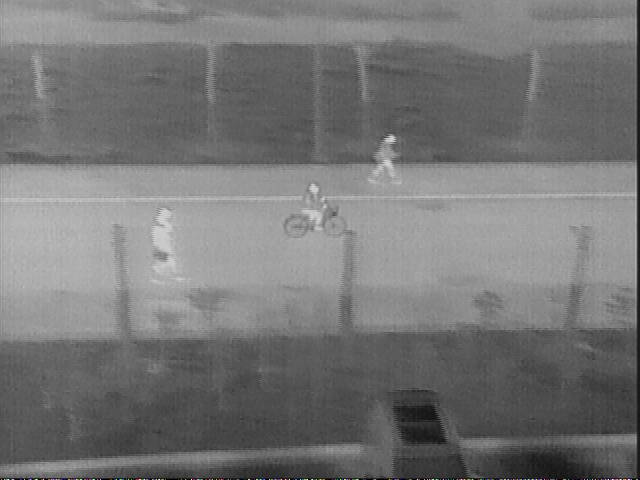

Supplement: S1 File — (ZIP) [file pone.0173613.s001.zip › infrared car and bicycle set/V34714.bmp]

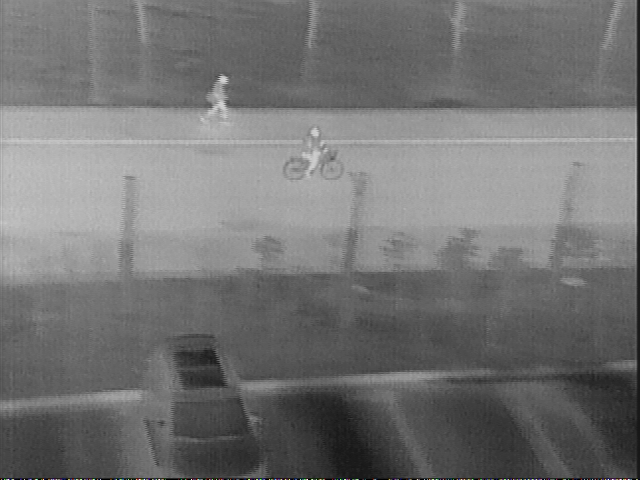

Supplement: S1 File — (ZIP) [file pone.0173613.s001.zip › infrared car and bicycle set/V34716.bmp]

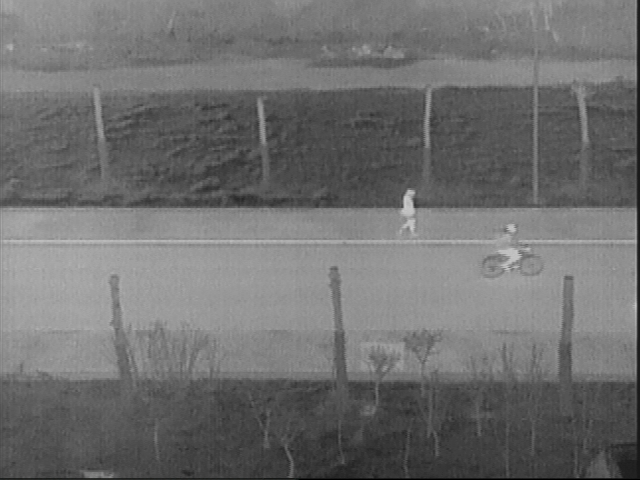

Supplement: S1 File — (ZIP) [file pone.0173613.s001.zip › infrared car and bicycle set/V34725.bmp]

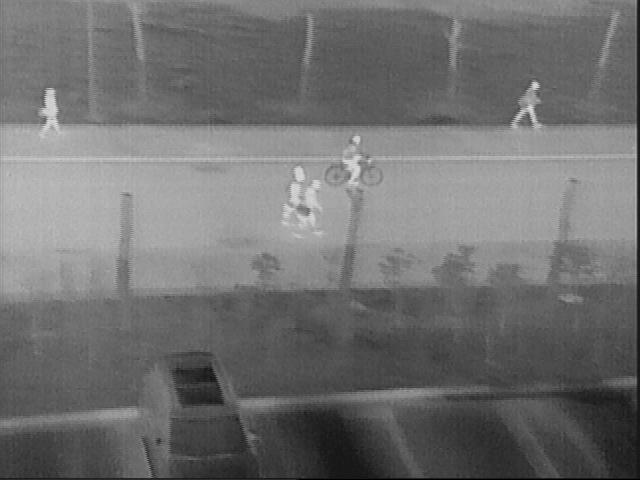

Supplement: S1 File — (ZIP) [file pone.0173613.s001.zip › infrared car and bicycle set/V34729.bmp]

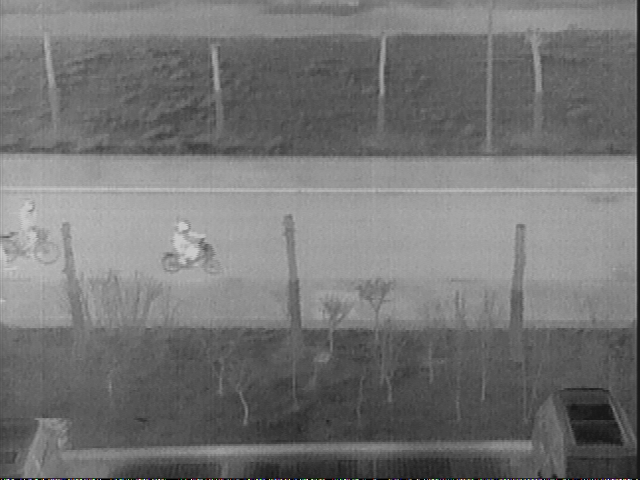

Supplement: S1 File — (ZIP) [file pone.0173613.s001.zip › infrared car and bicycle set/V34738.bmp]

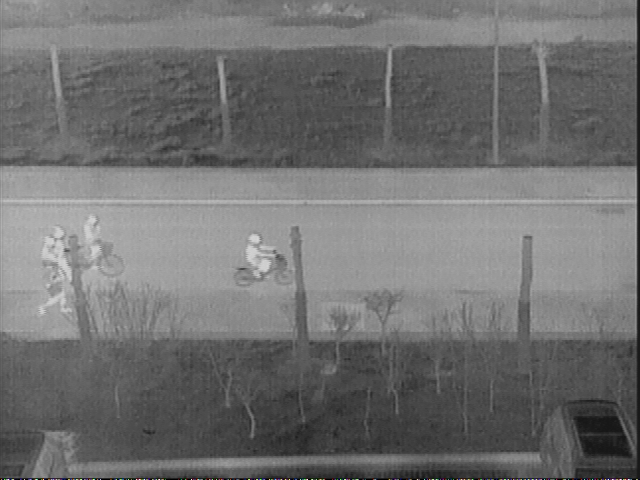

Supplement: S1 File — (ZIP) [file pone.0173613.s001.zip › infrared car and bicycle set/V34740.bmp]

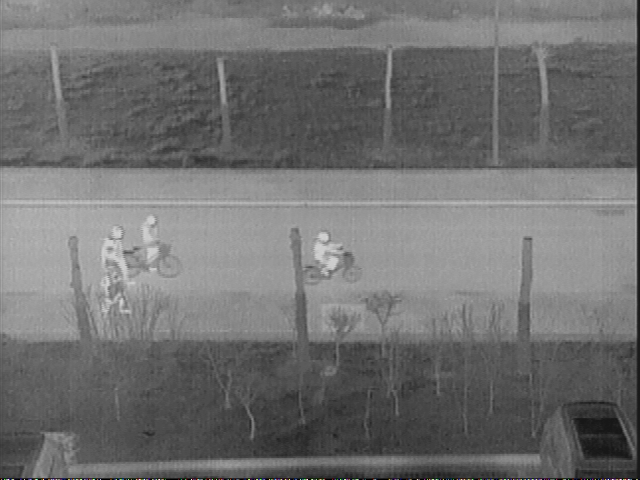

Supplement: S1 File — (ZIP) [file pone.0173613.s001.zip › infrared car and bicycle set/V34742.bmp]

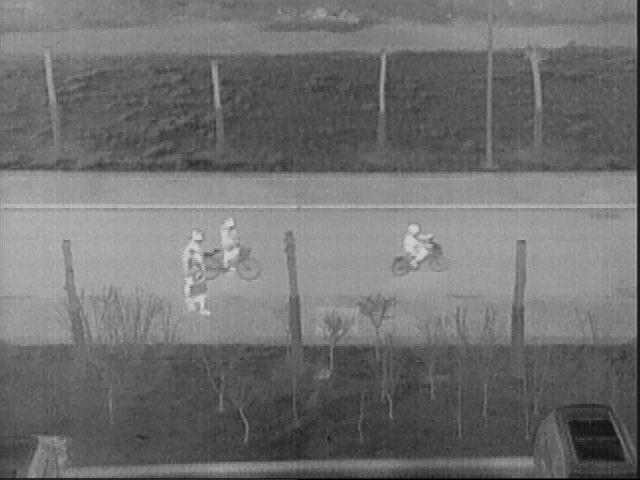

Supplement: S1 File — (ZIP) [file pone.0173613.s001.zip › infrared car and bicycle set/V34745.bmp]

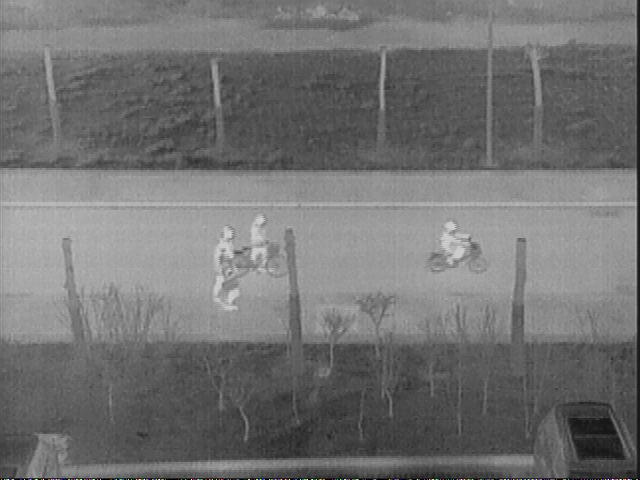

Supplement: S1 File — (ZIP) [file pone.0173613.s001.zip › infrared car and bicycle set/V34746.bmp]

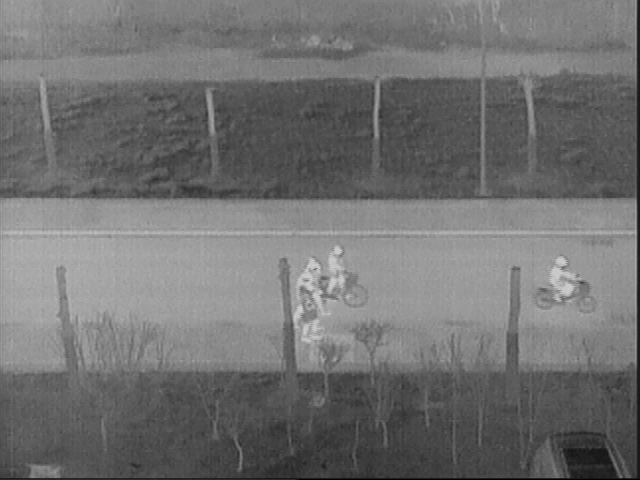

Supplement: S1 File — (ZIP) [file pone.0173613.s001.zip › infrared car and bicycle set/V34749.bmp]

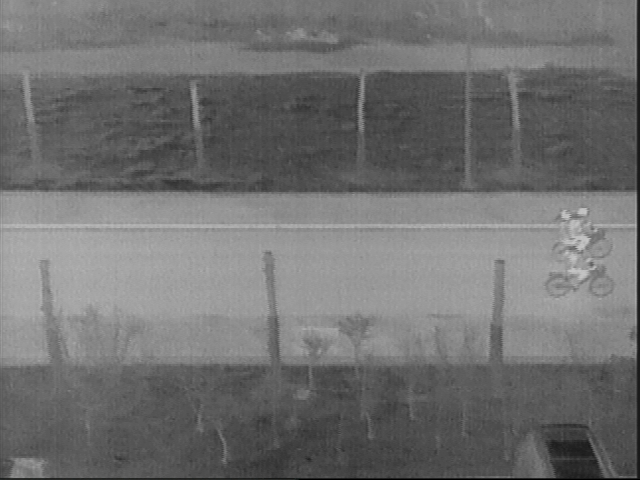

Supplement: S1 File — (ZIP) [file pone.0173613.s001.zip › infrared car and bicycle set/V34771.bmp]

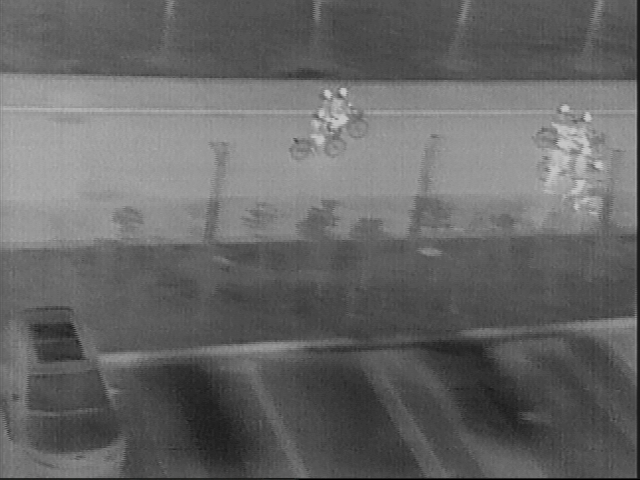

Supplement: S1 File — (ZIP) [file pone.0173613.s001.zip › infrared car and bicycle set/V34775.bmp]

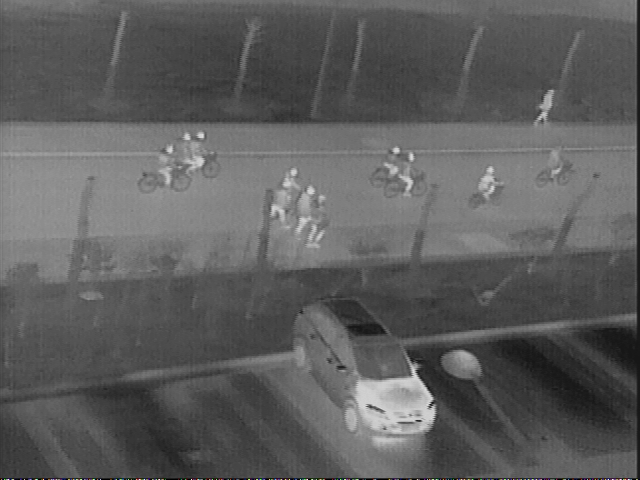

Supplement: S1 File — (ZIP) [file pone.0173613.s001.zip › infrared car and bicycle set/V34778.bmp]

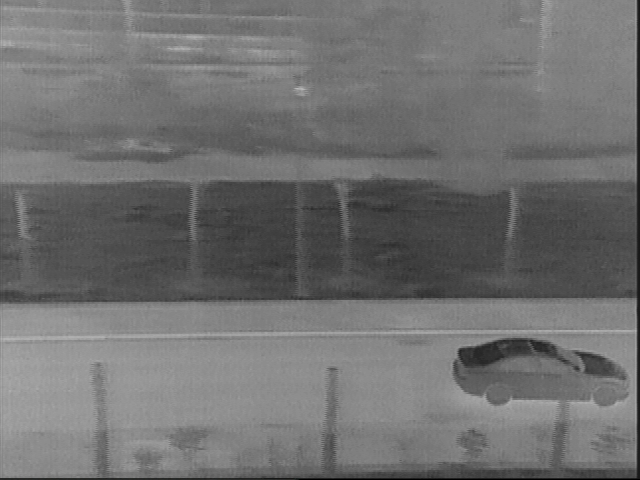

Supplement: S1 File — (ZIP) [file pone.0173613.s001.zip › infrared car and bicycle set/V34803.bmp]

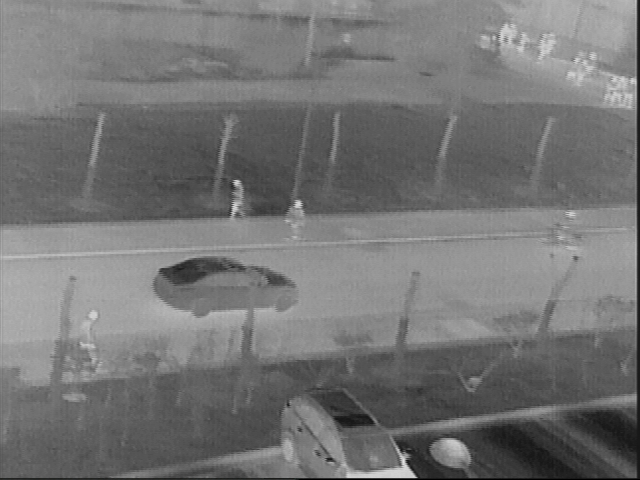

Supplement: S1 File — (ZIP) [file pone.0173613.s001.zip › infrared car and bicycle set/V34807.bmp]

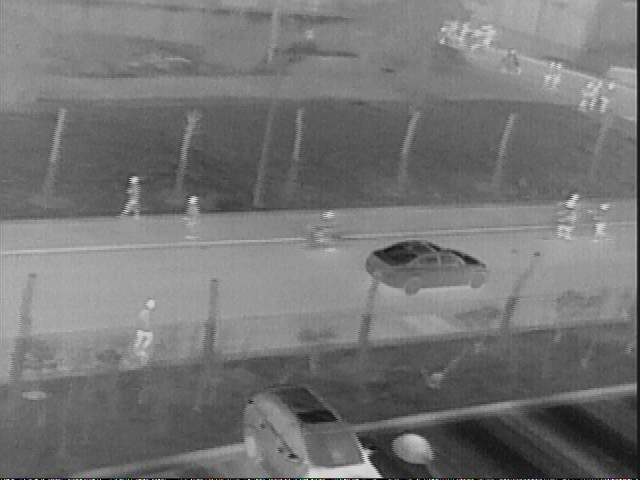

Supplement: S1 File — (ZIP) [file pone.0173613.s001.zip › infrared car and bicycle set/V34810.bmp]

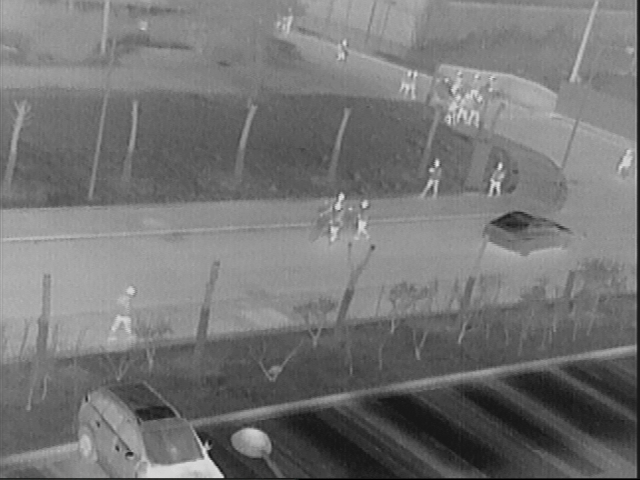

Supplement: S1 File — (ZIP) [file pone.0173613.s001.zip › infrared car and bicycle set/V34815.bmp]

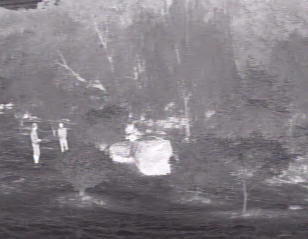

Supplement: S1 File — (ZIP) [file pone.0173613.s001.zip › infrared human set/I1 (0).bmp]

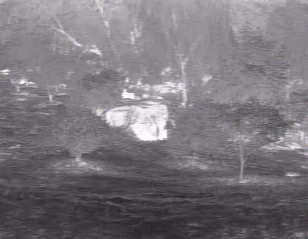

Supplement: S1 File — (ZIP) [file pone.0173613.s001.zip › infrared human set/I1 (1).bmp]

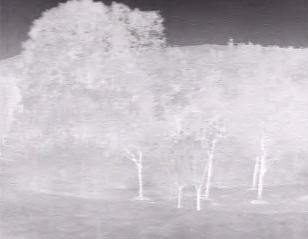

Supplement: S1 File — (ZIP) [file pone.0173613.s001.zip › infrared human set/I1 (10).bmp]

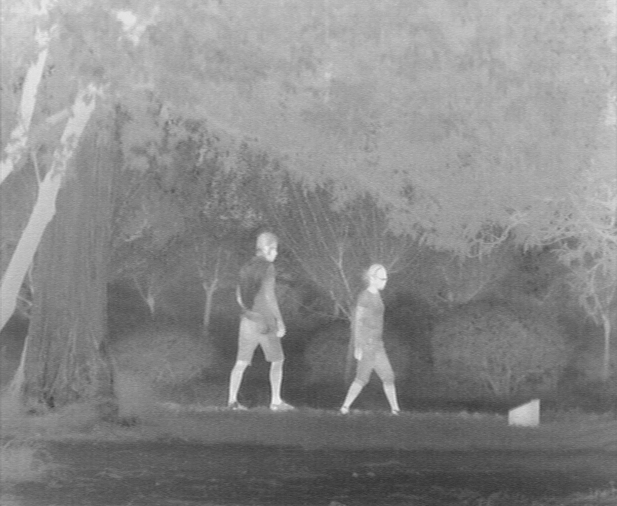

Supplement: S1 File — (ZIP) [file pone.0173613.s001.zip › infrared human set/I1 (100).bmp]

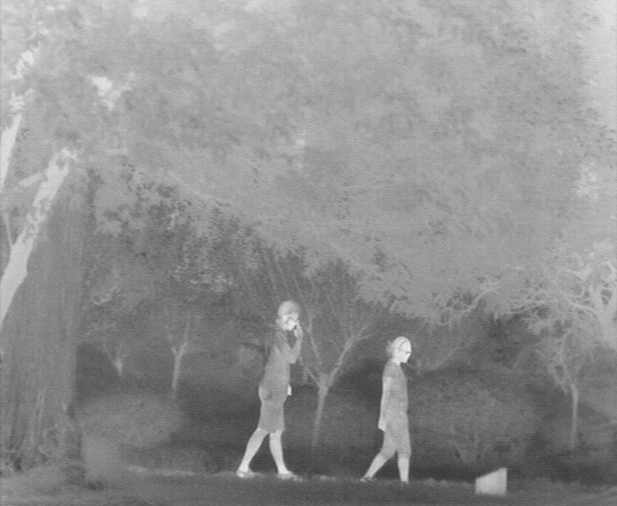

Supplement: S1 File — (ZIP) [file pone.0173613.s001.zip › infrared human set/I1 (101).bmp]

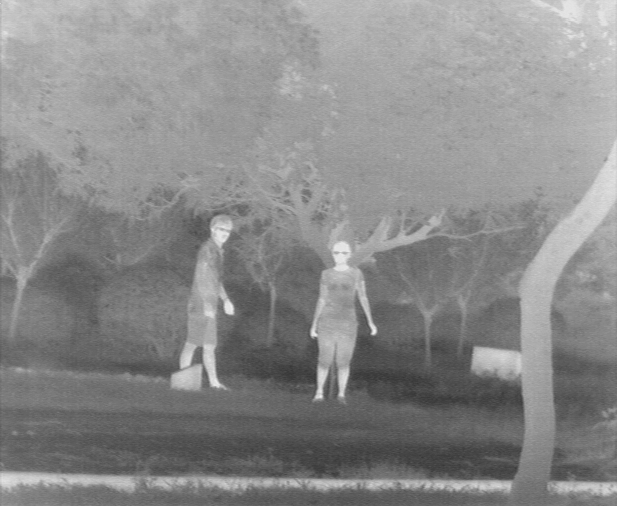

Supplement: S1 File — (ZIP) [file pone.0173613.s001.zip › infrared human set/I1 (102).bmp]

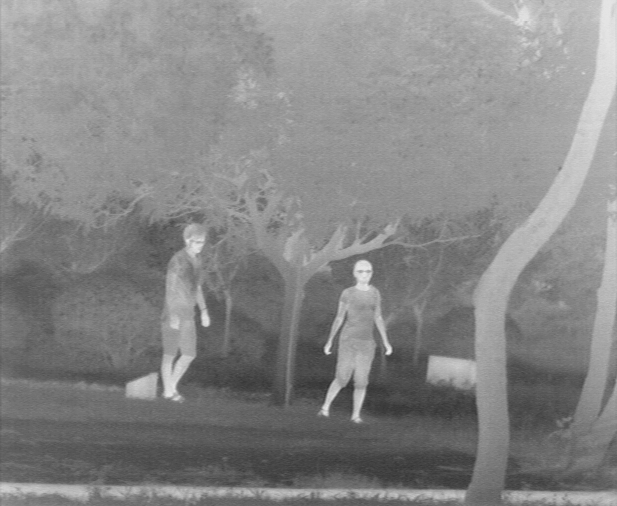

Supplement: S1 File — (ZIP) [file pone.0173613.s001.zip › infrared human set/I1 (103).bmp]

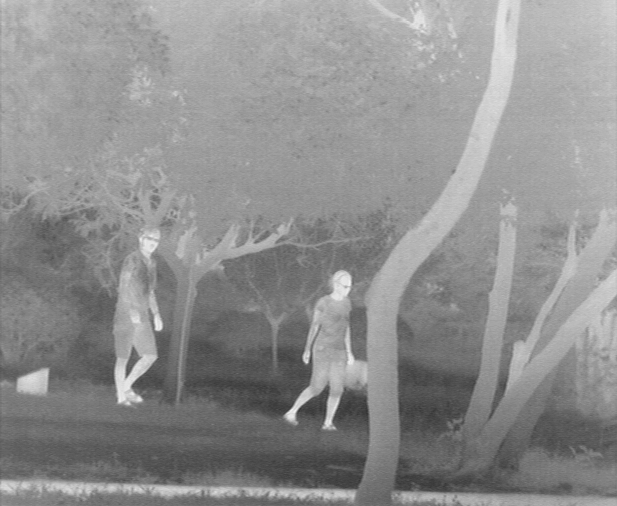

Supplement: S1 File — (ZIP) [file pone.0173613.s001.zip › infrared human set/I1 (104).bmp]

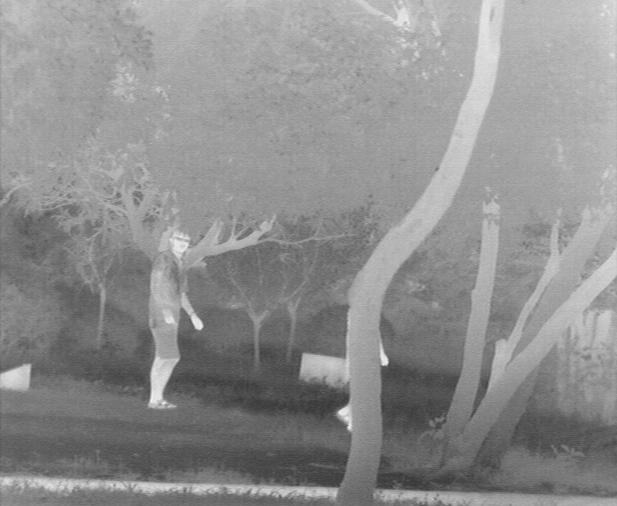

Supplement: S1 File — (ZIP) [file pone.0173613.s001.zip › infrared human set/I1 (105).bmp]

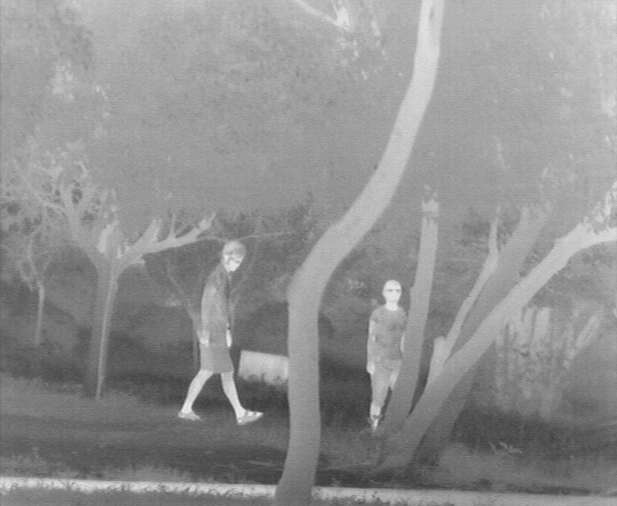

Supplement: S1 File — (ZIP) [file pone.0173613.s001.zip › infrared human set/I1 (106).bmp]

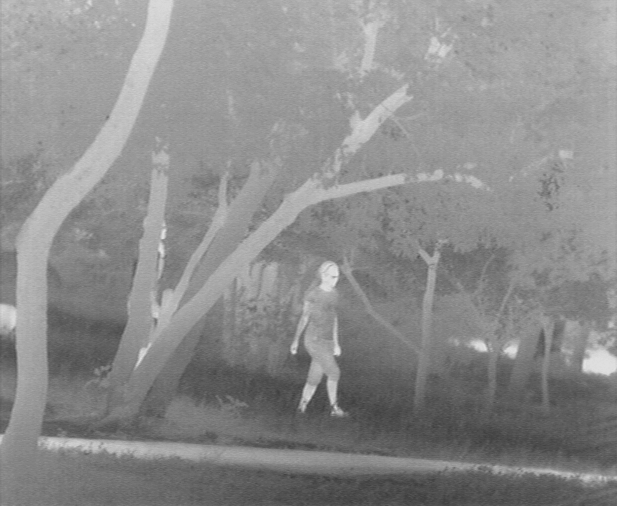

Supplement: S1 File — (ZIP) [file pone.0173613.s001.zip › infrared human set/I1 (107).bmp]

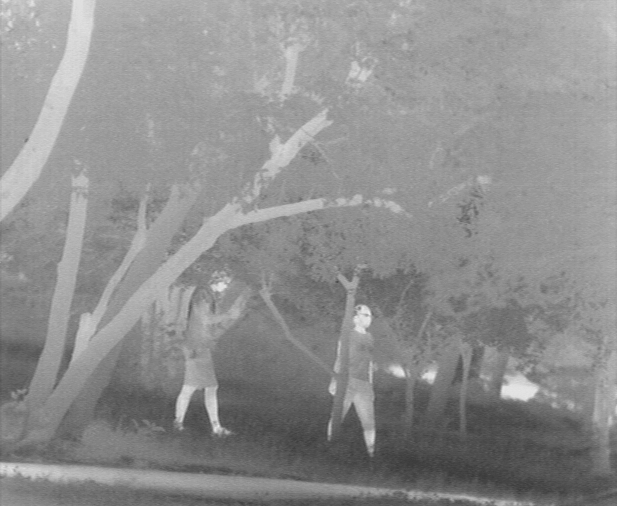

Supplement: S1 File — (ZIP) [file pone.0173613.s001.zip › infrared human set/I1 (108).bmp]

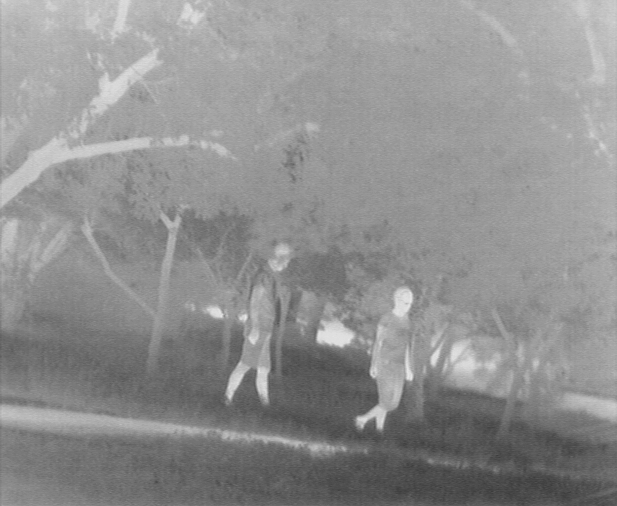

Supplement: S1 File — (ZIP) [file pone.0173613.s001.zip › infrared human set/I1 (109).bmp]

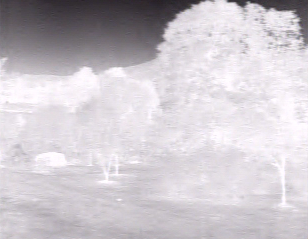

Supplement: S1 File — (ZIP) [file pone.0173613.s001.zip › infrared human set/I1 (11).bmp]

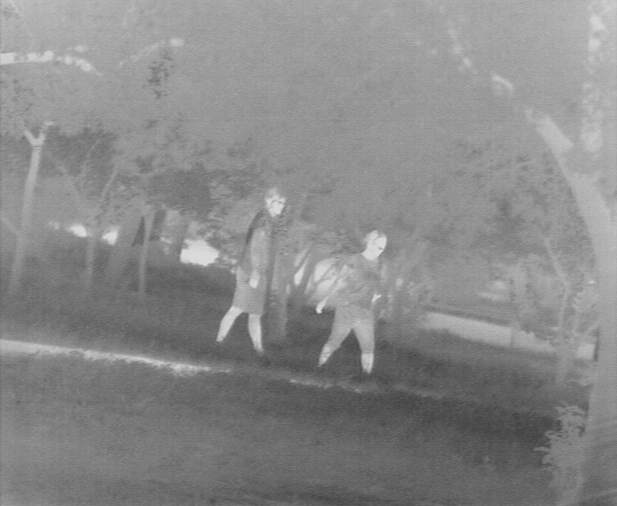

Supplement: S1 File — (ZIP) [file pone.0173613.s001.zip › infrared human set/I1 (110).bmp]

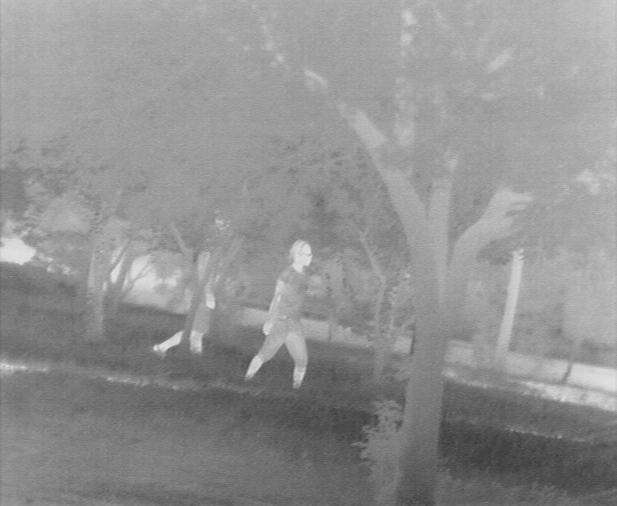

Supplement: S1 File — (ZIP) [file pone.0173613.s001.zip › infrared human set/I1 (111).bmp]

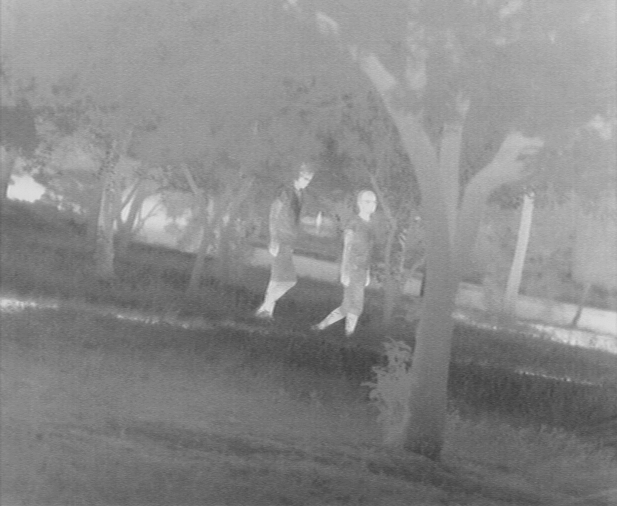

Supplement: S1 File — (ZIP) [file pone.0173613.s001.zip › infrared human set/I1 (112).bmp]

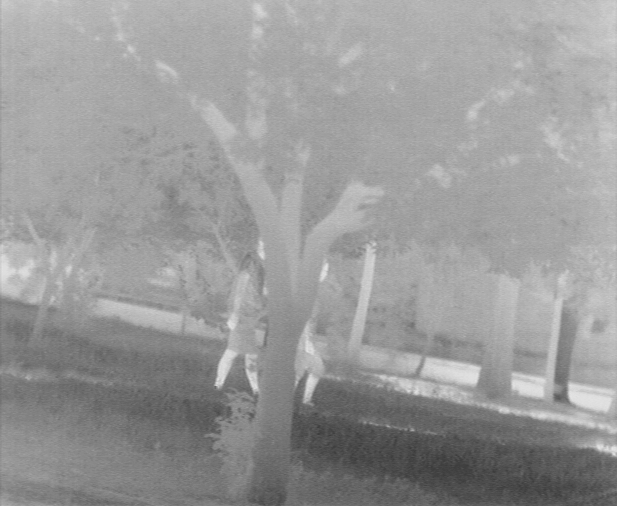

Supplement: S1 File — (ZIP) [file pone.0173613.s001.zip › infrared human set/I1 (113).bmp]

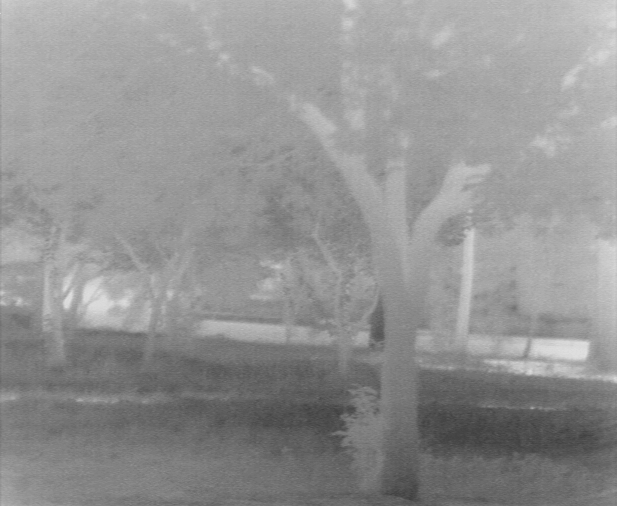

Supplement: S1 File — (ZIP) [file pone.0173613.s001.zip › infrared human set/I1 (114).bmp]

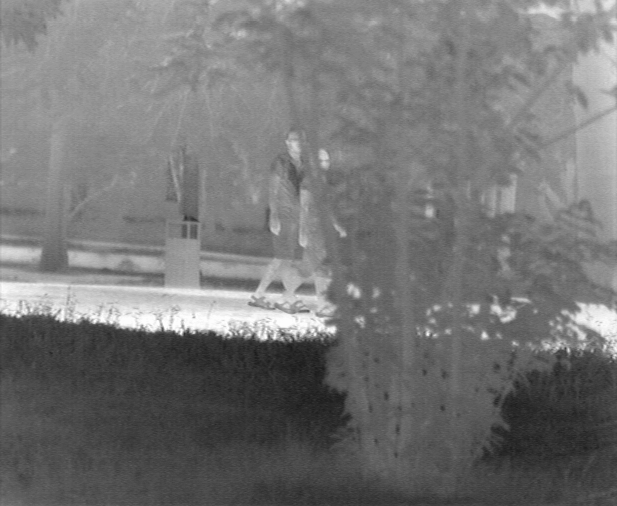

Supplement: S1 File — (ZIP) [file pone.0173613.s001.zip › infrared human set/I1 (115).bmp]

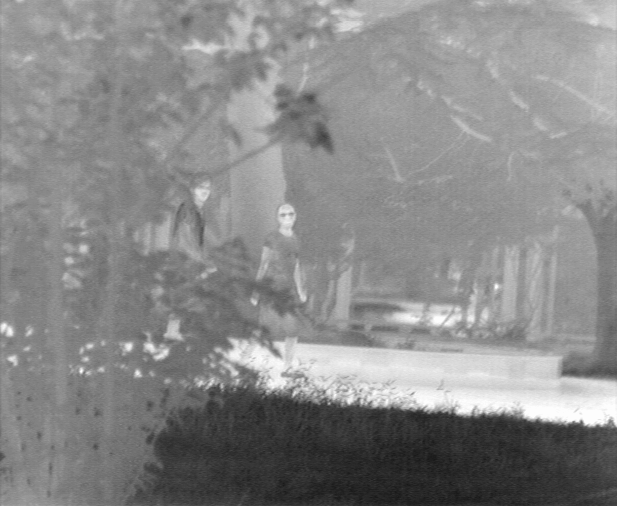

Supplement: S1 File — (ZIP) [file pone.0173613.s001.zip › infrared human set/I1 (116).bmp]

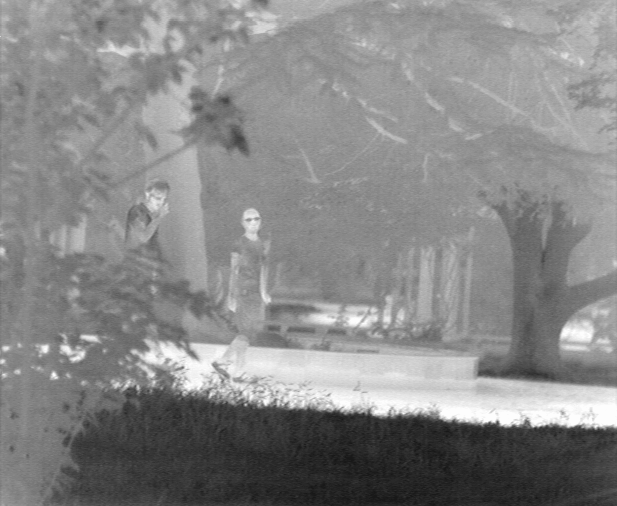

Supplement: S1 File — (ZIP) [file pone.0173613.s001.zip › infrared human set/I1 (117).bmp]

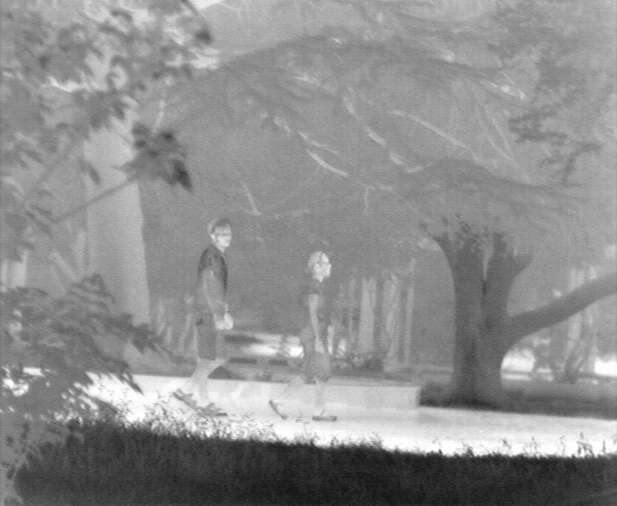

Supplement: S1 File — (ZIP) [file pone.0173613.s001.zip › infrared human set/I1 (118).bmp]

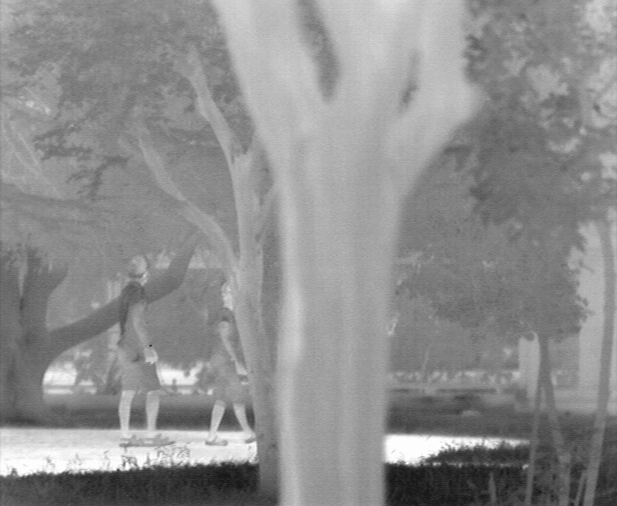

Supplement: S1 File — (ZIP) [file pone.0173613.s001.zip › infrared human set/I1 (119).bmp]

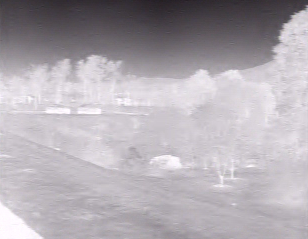

Supplement: S1 File — (ZIP) [file pone.0173613.s001.zip › infrared human set/I1 (12).bmp]

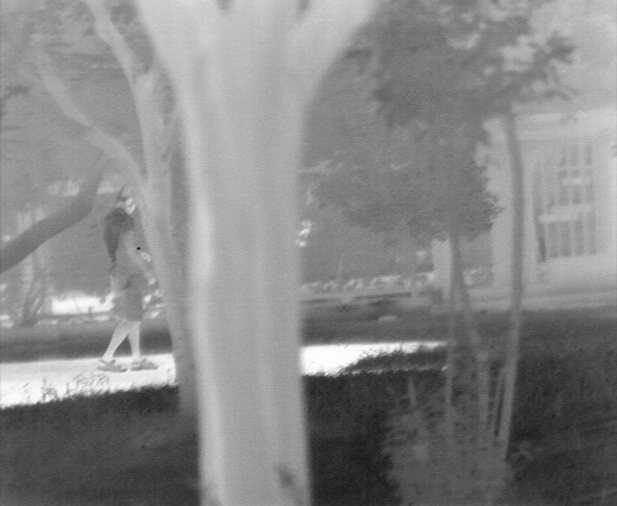

Supplement: S1 File — (ZIP) [file pone.0173613.s001.zip › infrared human set/I1 (120).bmp]

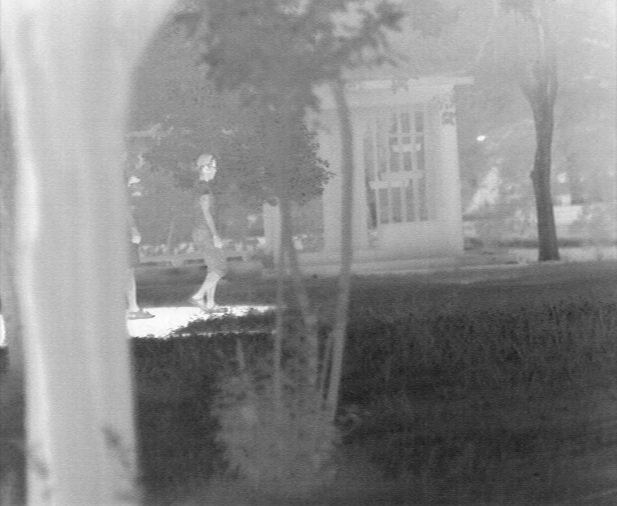

Supplement: S1 File — (ZIP) [file pone.0173613.s001.zip › infrared human set/I1 (121).bmp]

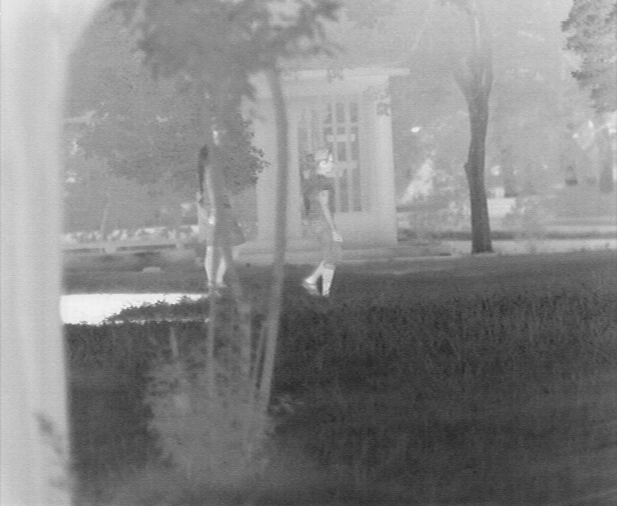

Supplement: S1 File — (ZIP) [file pone.0173613.s001.zip › infrared human set/I1 (122).bmp]

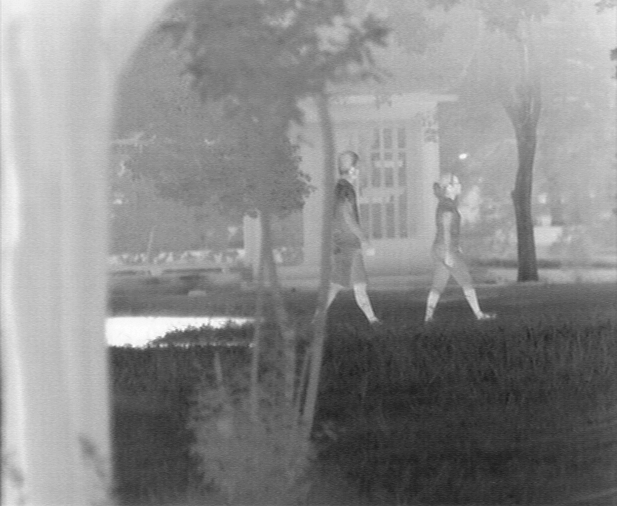

Supplement: S1 File — (ZIP) [file pone.0173613.s001.zip › infrared human set/I1 (123).bmp]

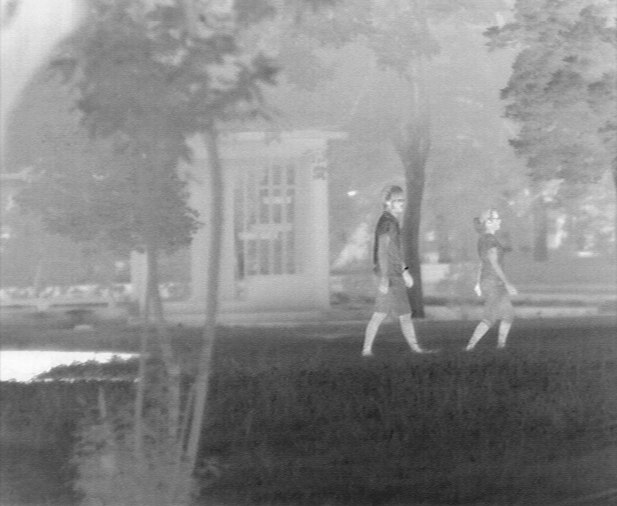

Supplement: S1 File — (ZIP) [file pone.0173613.s001.zip › infrared human set/I1 (124).bmp]

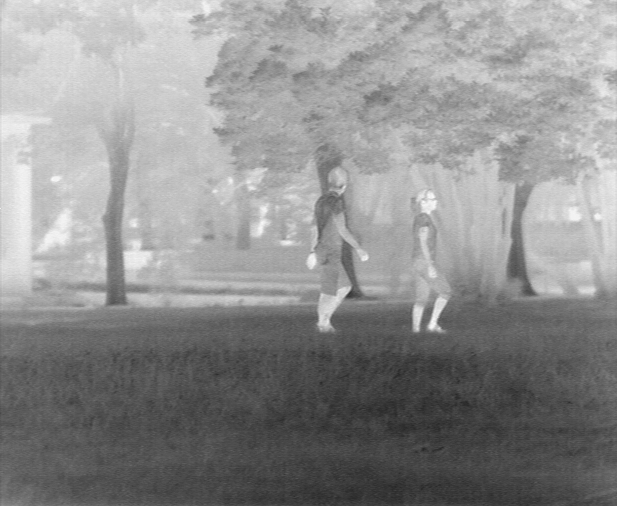

Supplement: S1 File — (ZIP) [file pone.0173613.s001.zip › infrared human set/I1 (125).bmp]

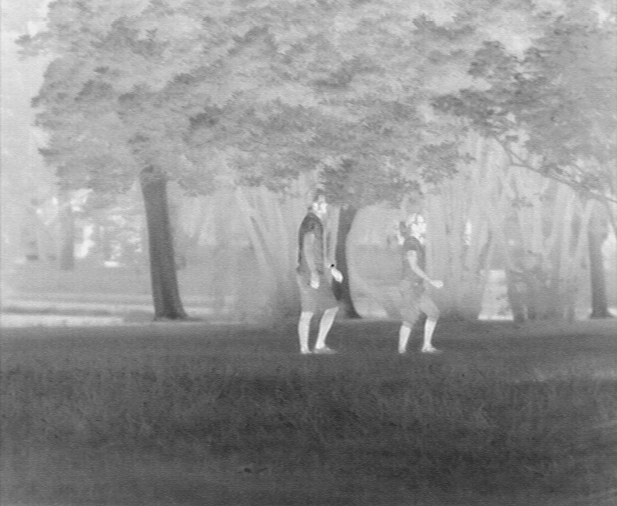

Supplement: S1 File — (ZIP) [file pone.0173613.s001.zip › infrared human set/I1 (126).bmp]

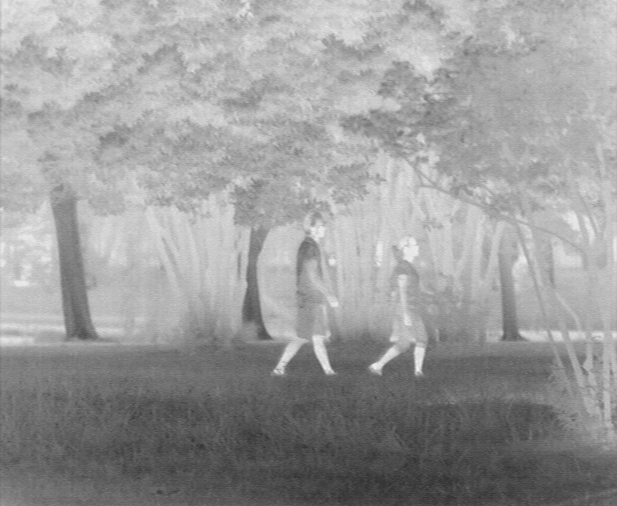

Supplement: S1 File — (ZIP) [file pone.0173613.s001.zip › infrared human set/I1 (127).bmp]

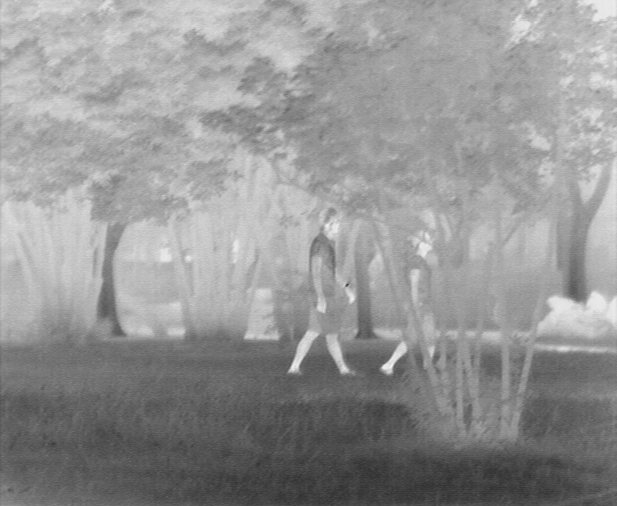

Supplement: S1 File — (ZIP) [file pone.0173613.s001.zip › infrared human set/I1 (128).bmp]

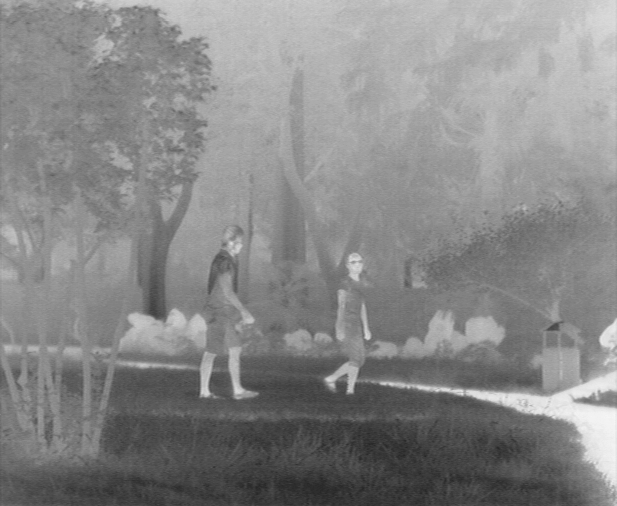

Supplement: S1 File — (ZIP) [file pone.0173613.s001.zip › infrared human set/I1 (129).bmp]

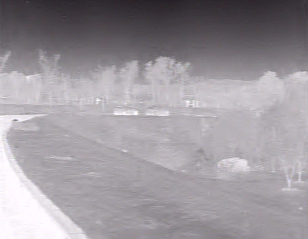

Supplement: S1 File — (ZIP) [file pone.0173613.s001.zip › infrared human set/I1 (13).bmp]

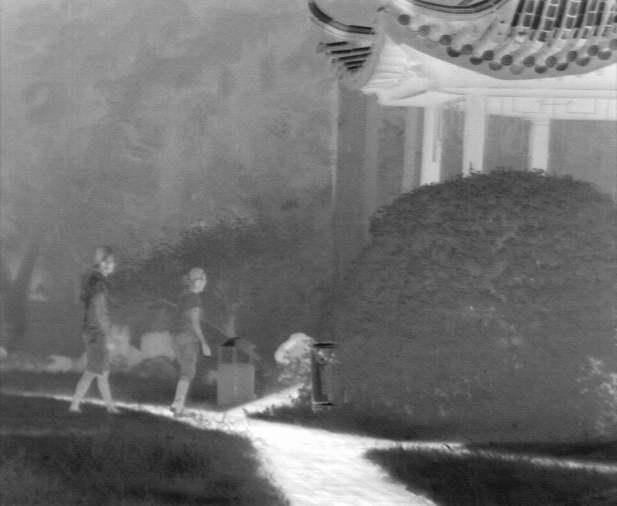

Supplement: S1 File — (ZIP) [file pone.0173613.s001.zip › infrared human set/I1 (130).bmp]

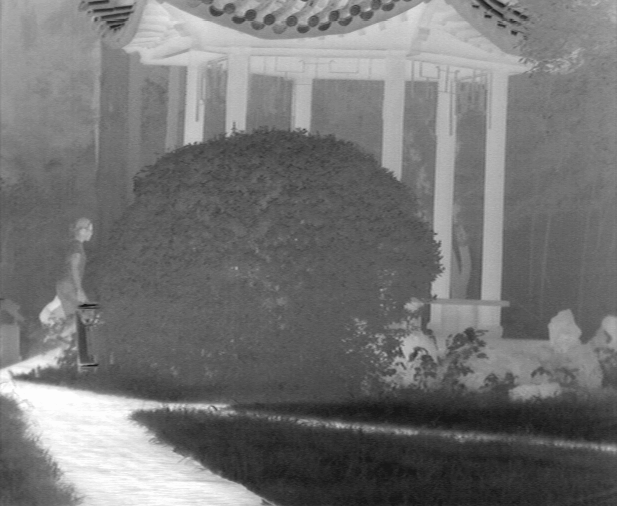

Supplement: S1 File — (ZIP) [file pone.0173613.s001.zip › infrared human set/I1 (131).bmp]

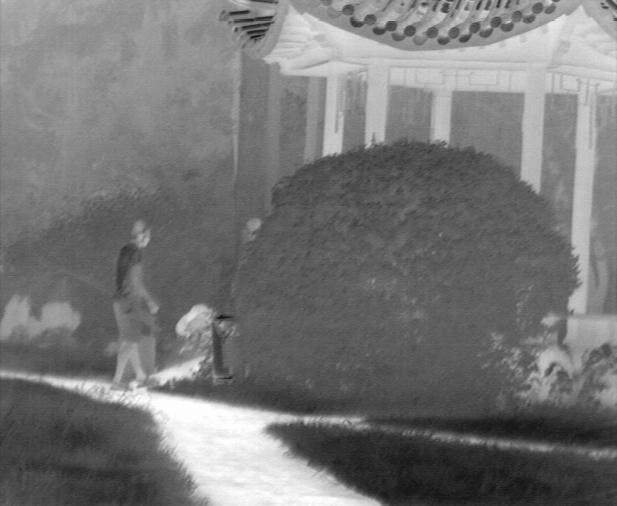

Supplement: S1 File — (ZIP) [file pone.0173613.s001.zip › infrared human set/I1 (132).bmp]

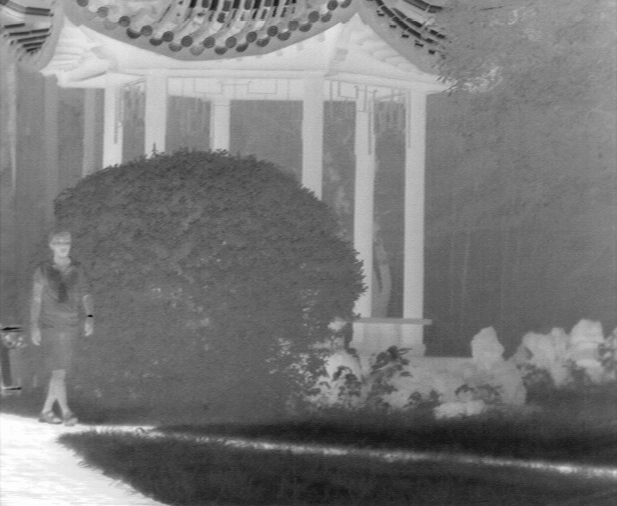

Supplement: S1 File — (ZIP) [file pone.0173613.s001.zip › infrared human set/I1 (133).bmp]

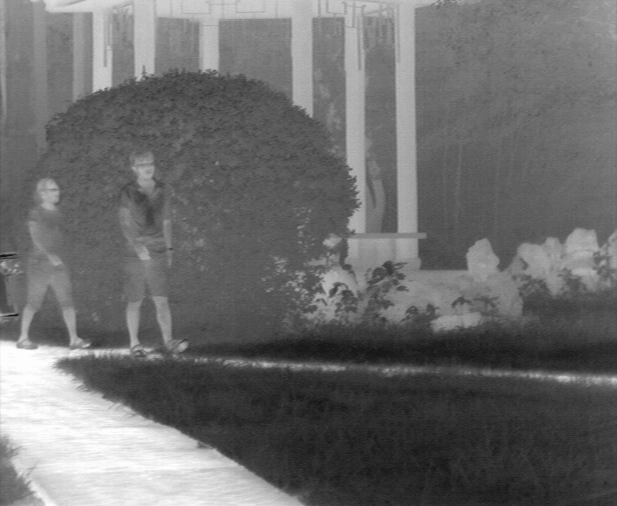

Supplement: S1 File — (ZIP) [file pone.0173613.s001.zip › infrared human set/I1 (134).bmp]

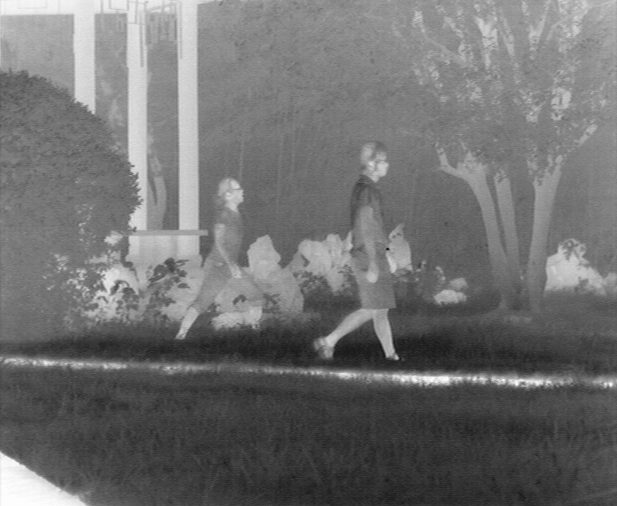

Supplement: S1 File — (ZIP) [file pone.0173613.s001.zip › infrared human set/I1 (135).bmp]

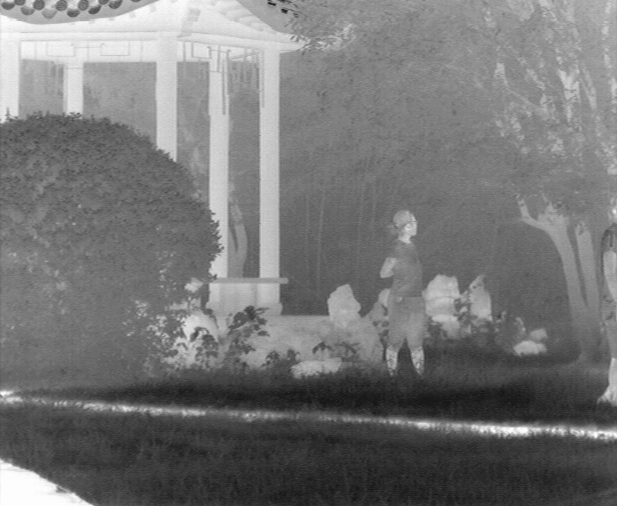

Supplement: S1 File — (ZIP) [file pone.0173613.s001.zip › infrared human set/I1 (136).bmp]

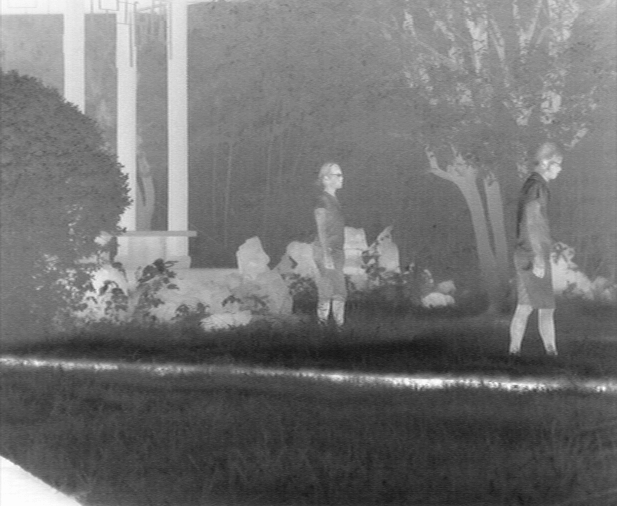

Supplement: S1 File — (ZIP) [file pone.0173613.s001.zip › infrared human set/I1 (137).bmp]

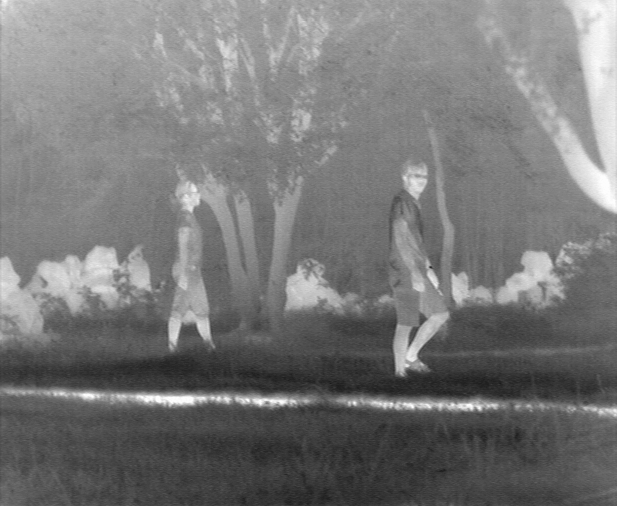

Supplement: S1 File — (ZIP) [file pone.0173613.s001.zip › infrared human set/I1 (138).bmp]
