# Supplementary figures and images for: Local structure preserving sparse coding for infrared target recognition (part 1 of 2)
Source: PLoS One. 2017 Mar 21;12(3):e0173613. doi: 10.1371/journal.pone.0173613 (PMC5360252; doi:10.1371/journal.pone.0173613)

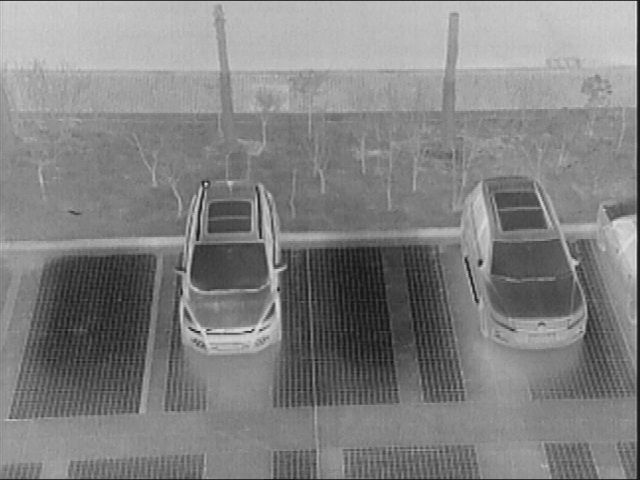

Supplement: S1 File — (ZIP) [file pone.0173613.s001.zip › infrared car and bicycle set/V1056.bmp]

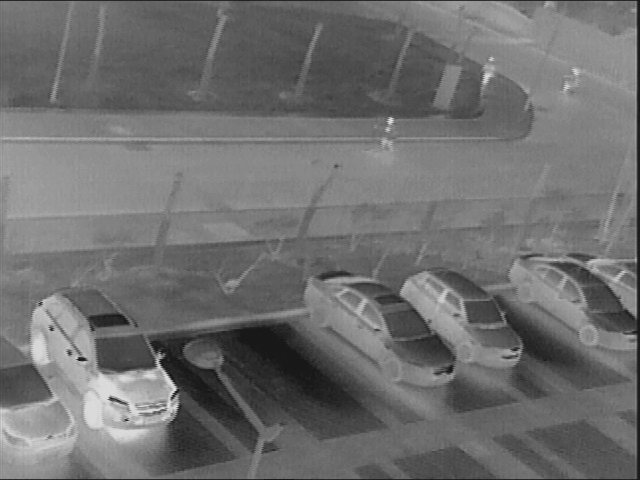

Supplement: S1 File — (ZIP) [file pone.0173613.s001.zip › infrared car and bicycle set/V1061.bmp]

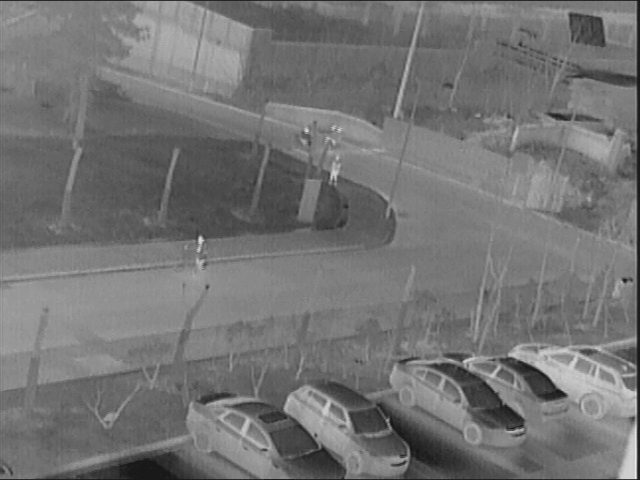

Supplement: S1 File — (ZIP) [file pone.0173613.s001.zip › infrared car and bicycle set/V1067.bmp]

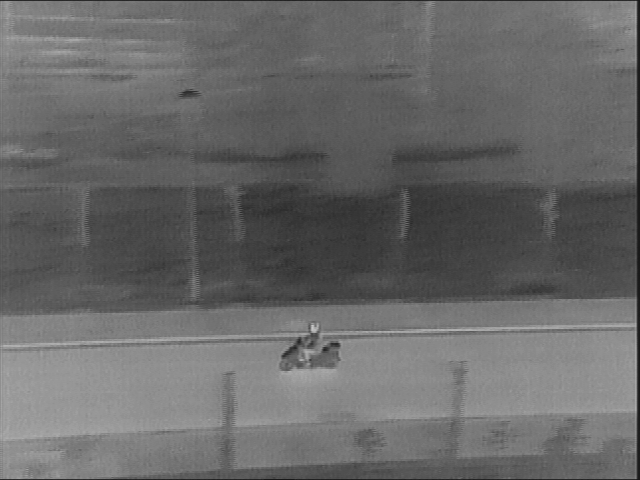

Supplement: S1 File — (ZIP) [file pone.0173613.s001.zip › infrared car and bicycle set/V1099.bmp]

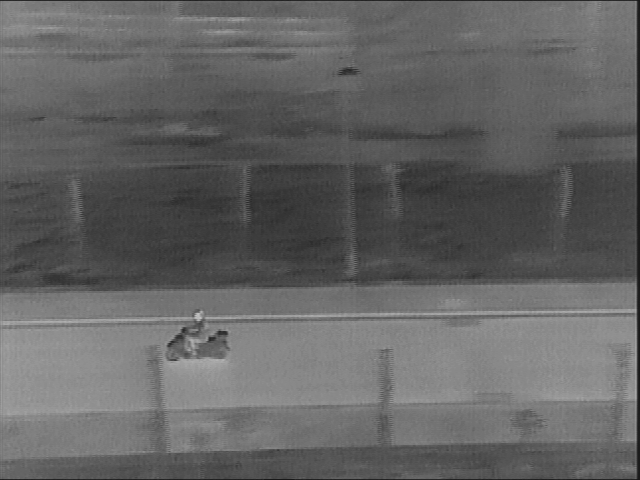

Supplement: S1 File — (ZIP) [file pone.0173613.s001.zip › infrared car and bicycle set/V1102.bmp]

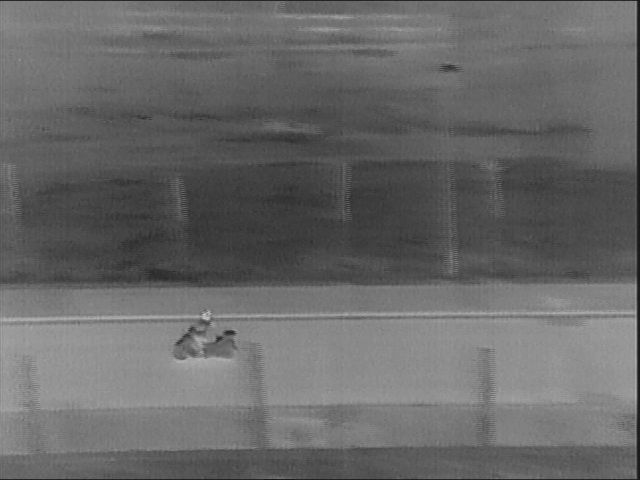

Supplement: S1 File — (ZIP) [file pone.0173613.s001.zip › infrared car and bicycle set/V1103.bmp]

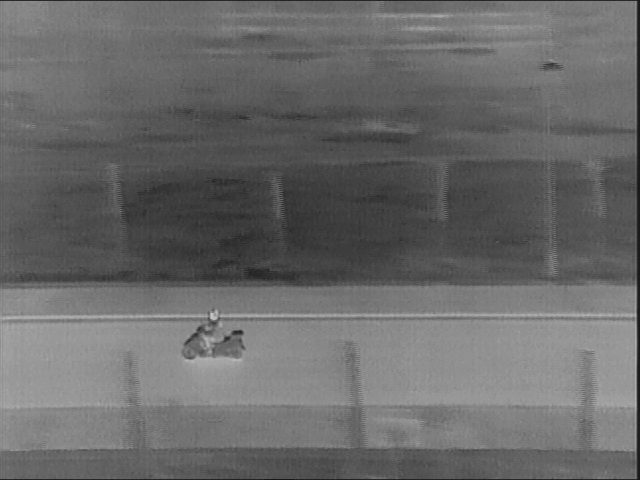

Supplement: S1 File — (ZIP) [file pone.0173613.s001.zip › infrared car and bicycle set/V1104.bmp]

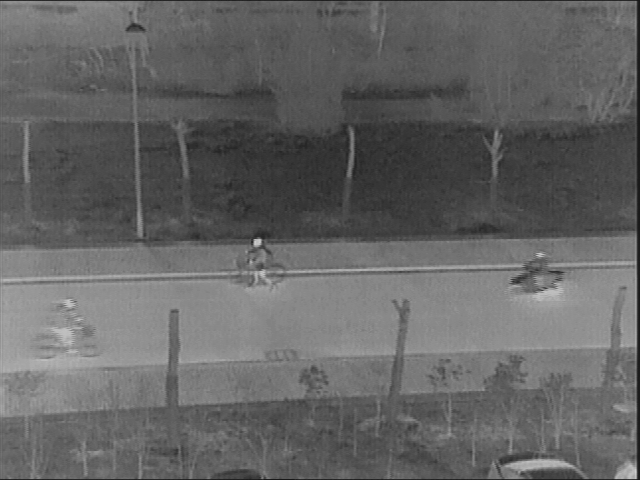

Supplement: S1 File — (ZIP) [file pone.0173613.s001.zip › infrared car and bicycle set/V1128.bmp]

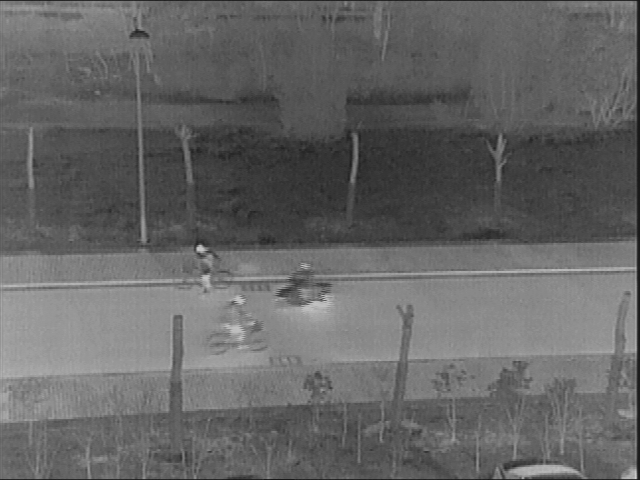

Supplement: S1 File — (ZIP) [file pone.0173613.s001.zip › infrared car and bicycle set/V1131.bmp]

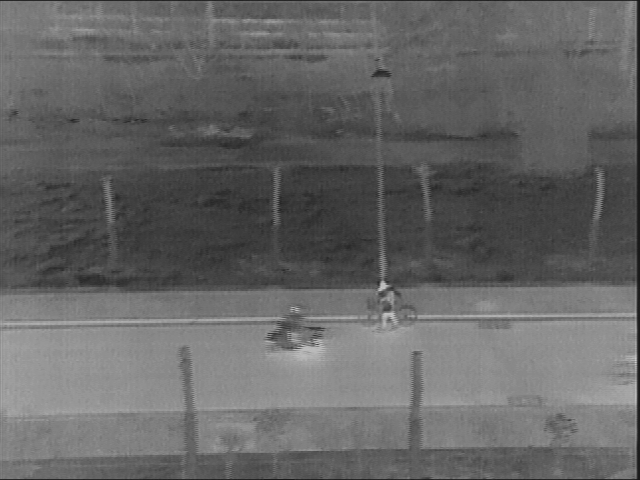

Supplement: S1 File — (ZIP) [file pone.0173613.s001.zip › infrared car and bicycle set/V1134.bmp]

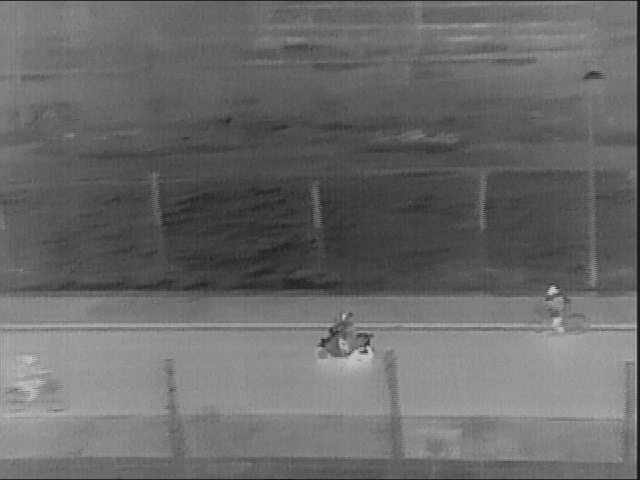

Supplement: S1 File — (ZIP) [file pone.0173613.s001.zip › infrared car and bicycle set/V1136.bmp]

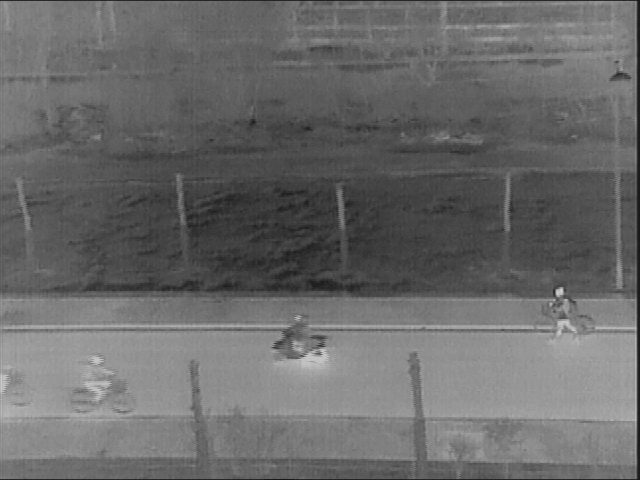

Supplement: S1 File — (ZIP) [file pone.0173613.s001.zip › infrared car and bicycle set/V1137.bmp]

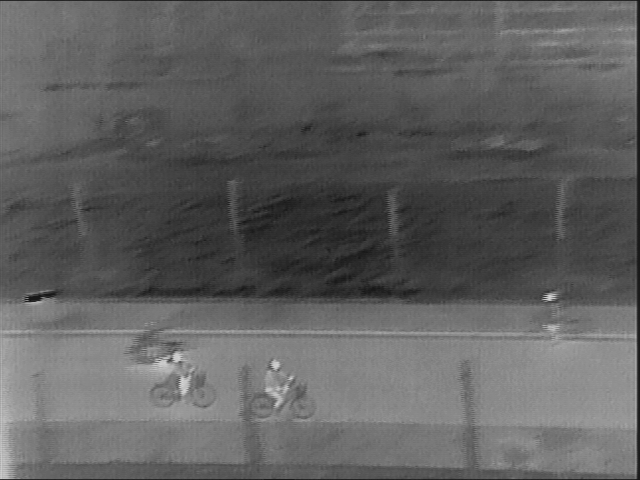

Supplement: S1 File — (ZIP) [file pone.0173613.s001.zip › infrared car and bicycle set/V1140.bmp]

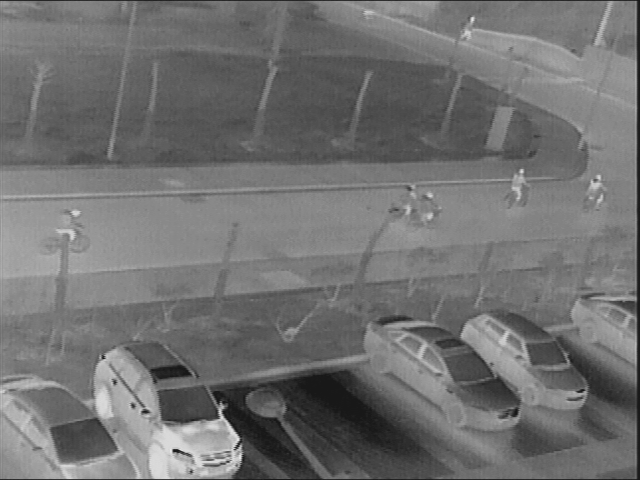

Supplement: S1 File — (ZIP) [file pone.0173613.s001.zip › infrared car and bicycle set/V1177.bmp]

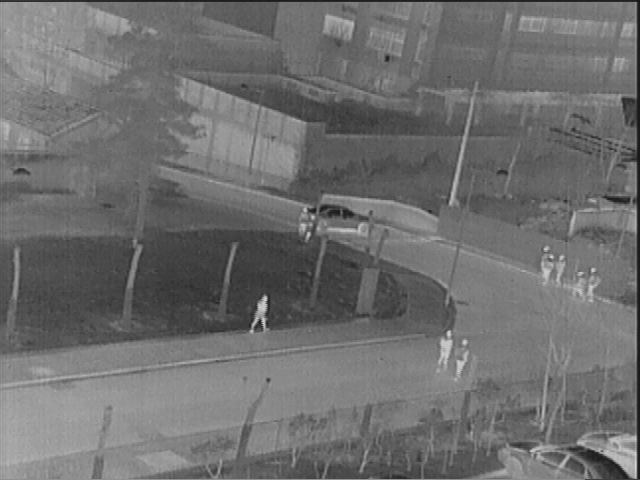

Supplement: S1 File — (ZIP) [file pone.0173613.s001.zip › infrared car and bicycle set/V20390.bmp]

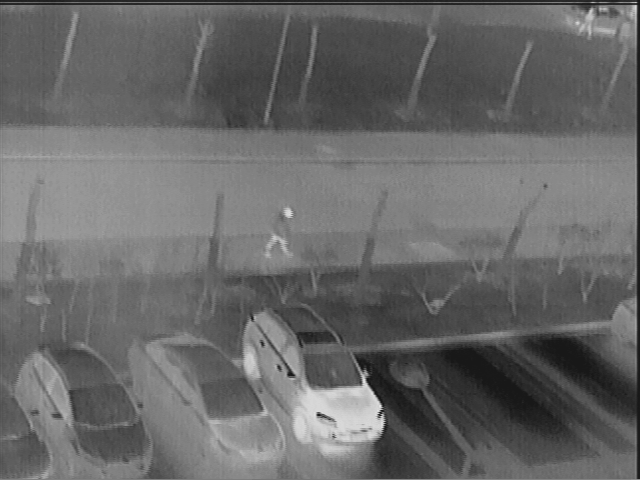

Supplement: S1 File — (ZIP) [file pone.0173613.s001.zip › infrared car and bicycle set/V20421.bmp]

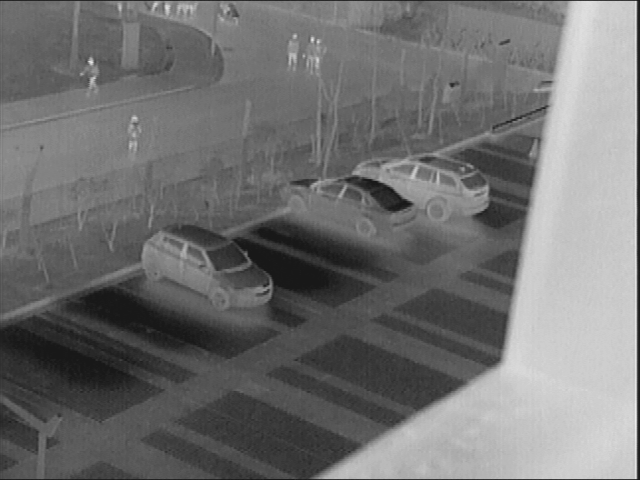

Supplement: S1 File — (ZIP) [file pone.0173613.s001.zip › infrared car and bicycle set/V20510.bmp]

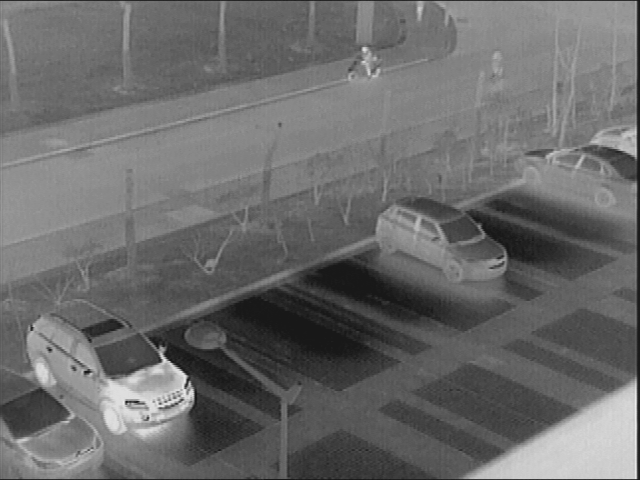

Supplement: S1 File — (ZIP) [file pone.0173613.s001.zip › infrared car and bicycle set/V20535.bmp]

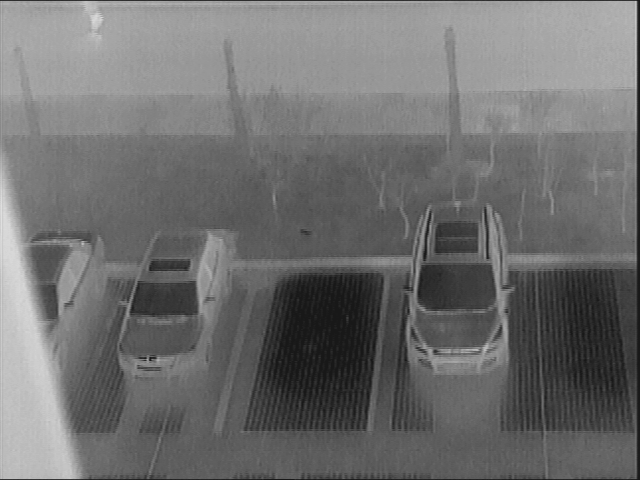

Supplement: S1 File — (ZIP) [file pone.0173613.s001.zip › infrared car and bicycle set/V20543.bmp]

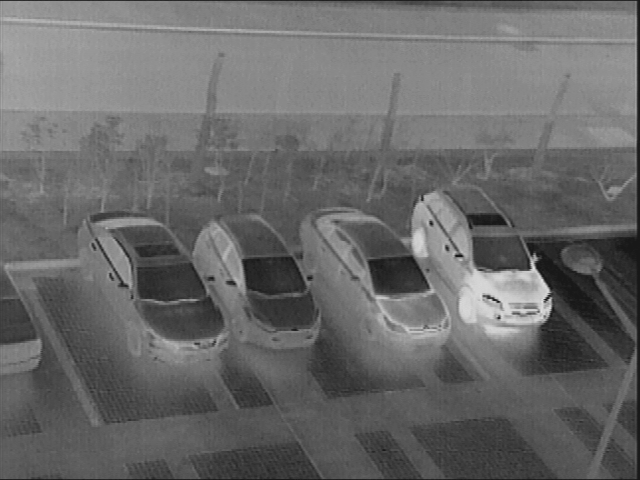

Supplement: S1 File — (ZIP) [file pone.0173613.s001.zip › infrared car and bicycle set/V20554.bmp]

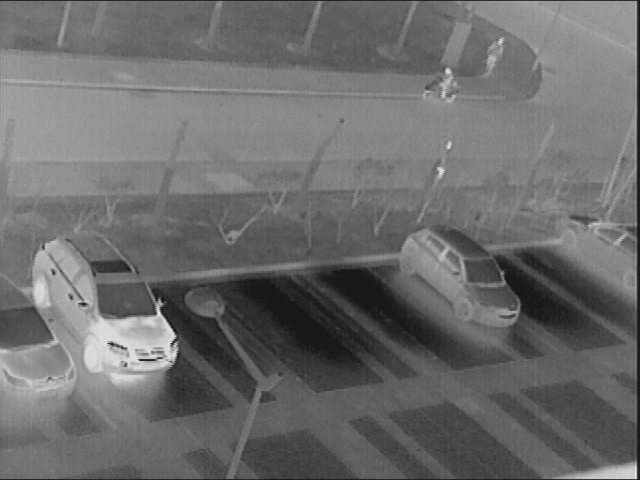

Supplement: S1 File — (ZIP) [file pone.0173613.s001.zip › infrared car and bicycle set/V20557.bmp]

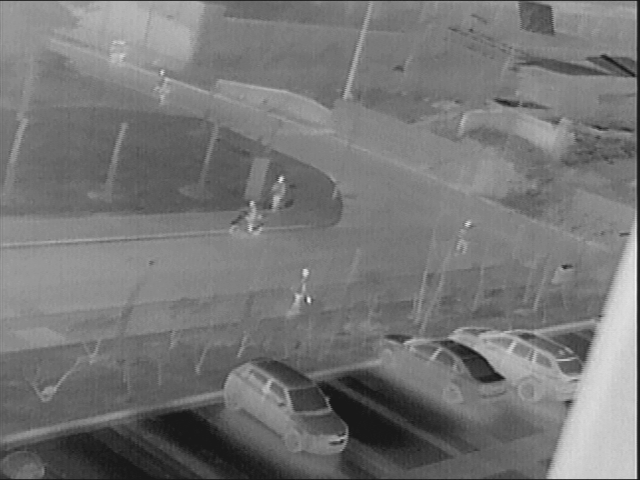

Supplement: S1 File — (ZIP) [file pone.0173613.s001.zip › infrared car and bicycle set/V20560.bmp]

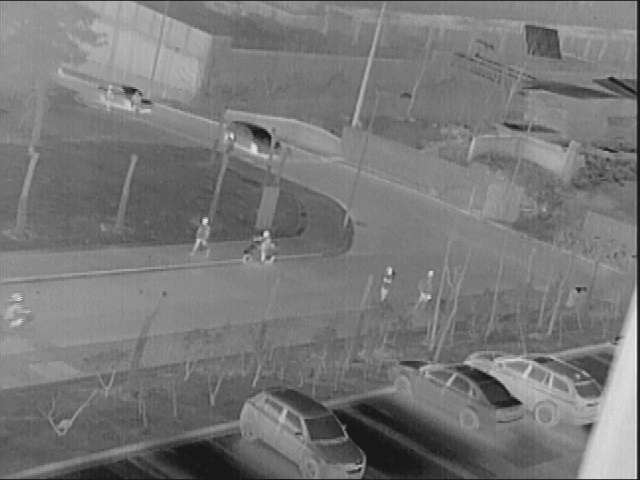

Supplement: S1 File — (ZIP) [file pone.0173613.s001.zip › infrared car and bicycle set/V20570.bmp]

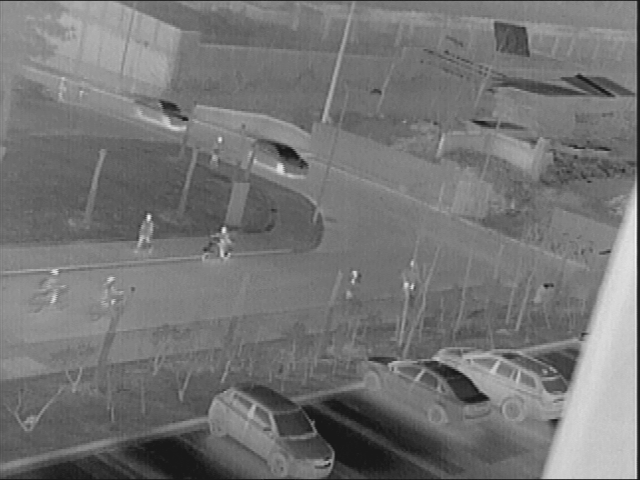

Supplement: S1 File — (ZIP) [file pone.0173613.s001.zip › infrared car and bicycle set/V20572.bmp]

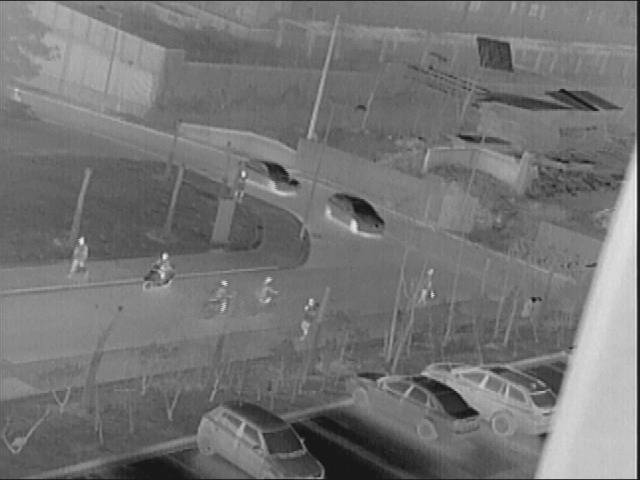

Supplement: S1 File — (ZIP) [file pone.0173613.s001.zip › infrared car and bicycle set/V20576.bmp]

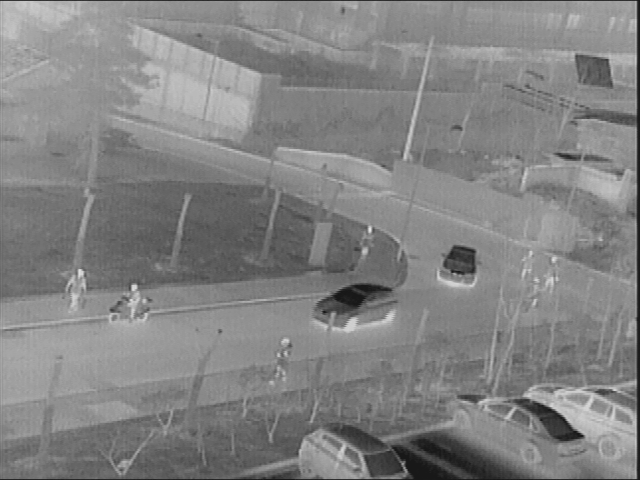

Supplement: S1 File — (ZIP) [file pone.0173613.s001.zip › infrared car and bicycle set/V20585.bmp]

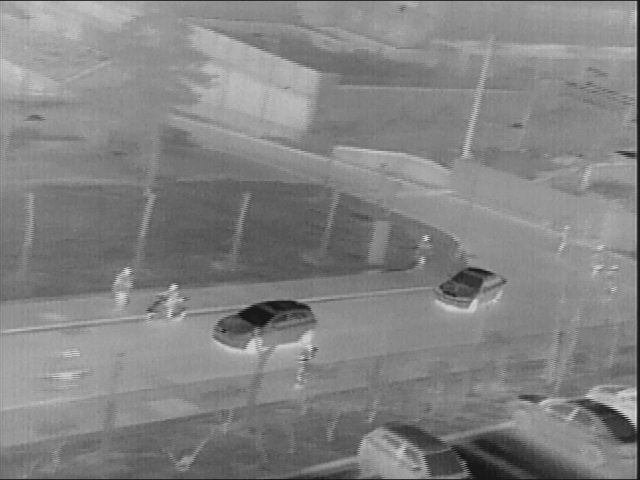

Supplement: S1 File — (ZIP) [file pone.0173613.s001.zip › infrared car and bicycle set/V20587.bmp]

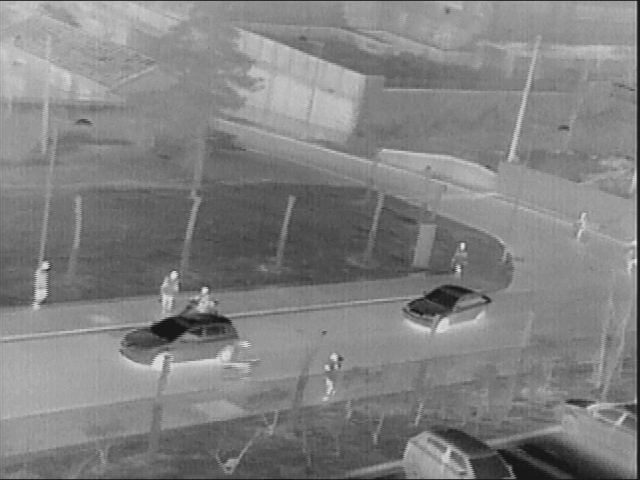

Supplement: S1 File — (ZIP) [file pone.0173613.s001.zip › infrared car and bicycle set/V20588.bmp]

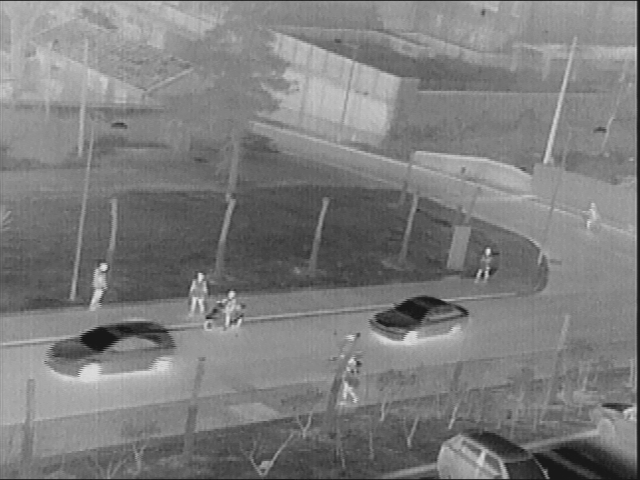

Supplement: S1 File — (ZIP) [file pone.0173613.s001.zip › infrared car and bicycle set/V20589.bmp]

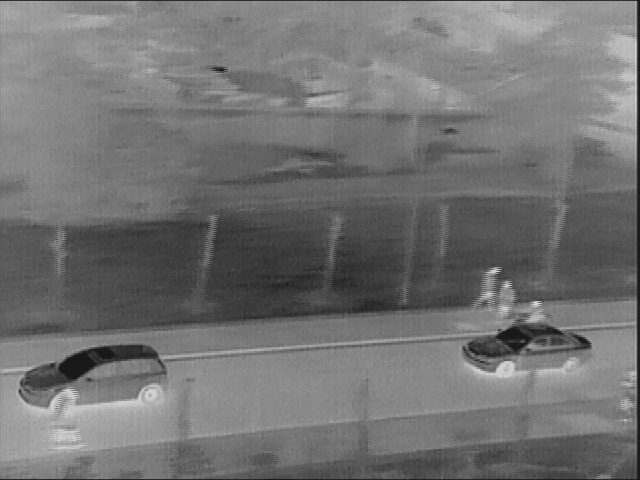

Supplement: S1 File — (ZIP) [file pone.0173613.s001.zip › infrared car and bicycle set/V20592.bmp]

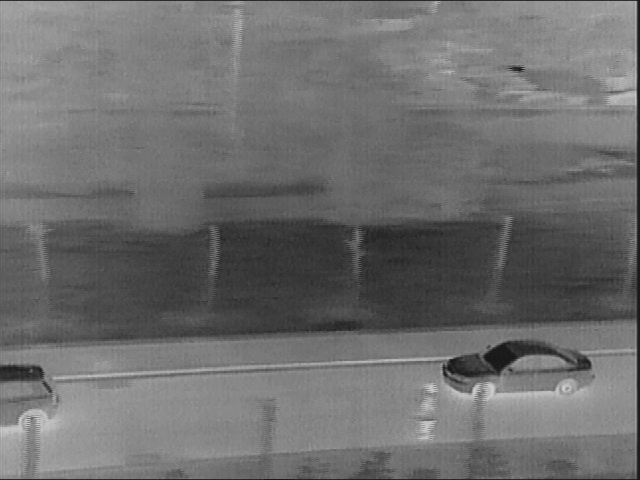

Supplement: S1 File — (ZIP) [file pone.0173613.s001.zip › infrared car and bicycle set/V20595.bmp]

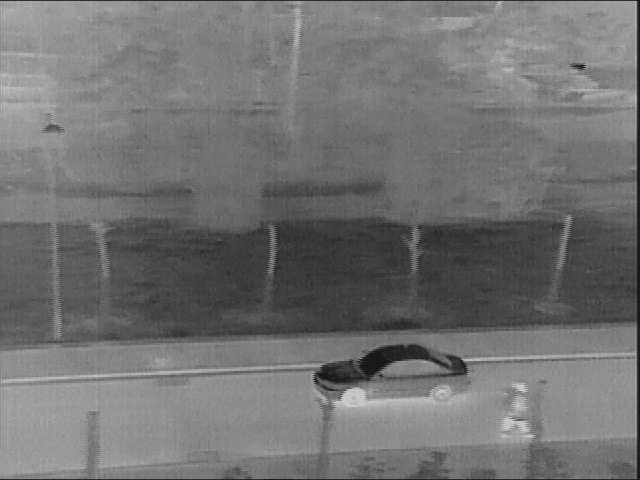

Supplement: S1 File — (ZIP) [file pone.0173613.s001.zip › infrared car and bicycle set/V20596.bmp]

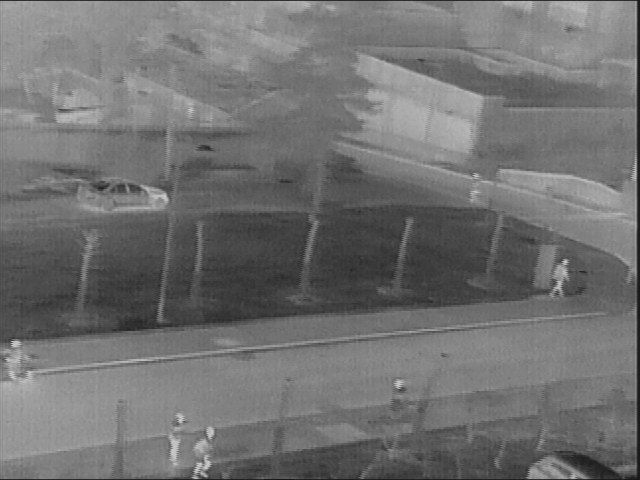

Supplement: S1 File — (ZIP) [file pone.0173613.s001.zip › infrared car and bicycle set/V20611.bmp]

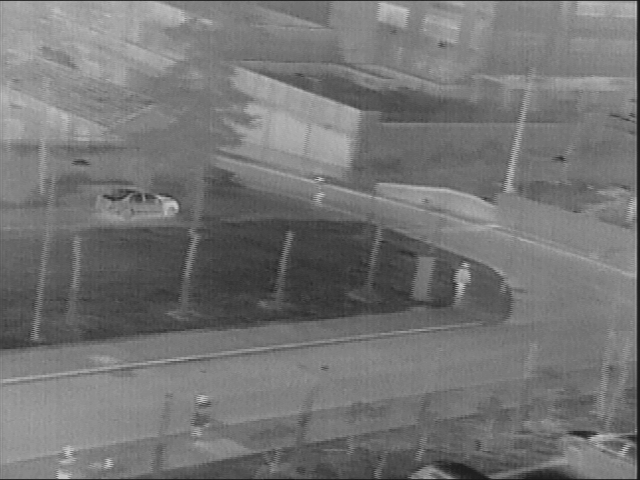

Supplement: S1 File — (ZIP) [file pone.0173613.s001.zip › infrared car and bicycle set/V20614.bmp]

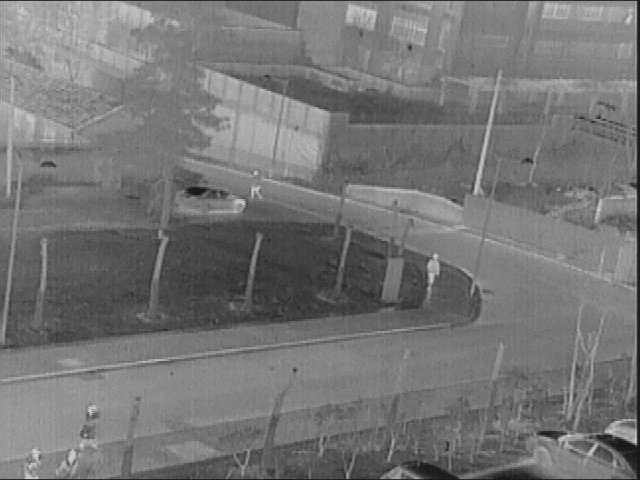

Supplement: S1 File — (ZIP) [file pone.0173613.s001.zip › infrared car and bicycle set/V20617.bmp]

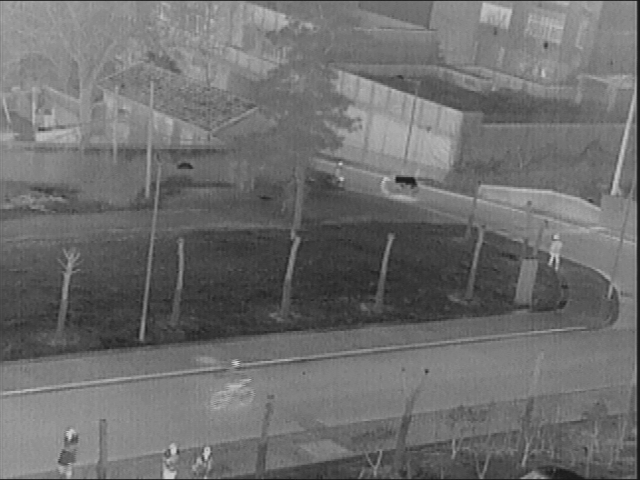

Supplement: S1 File — (ZIP) [file pone.0173613.s001.zip › infrared car and bicycle set/V20623.bmp]

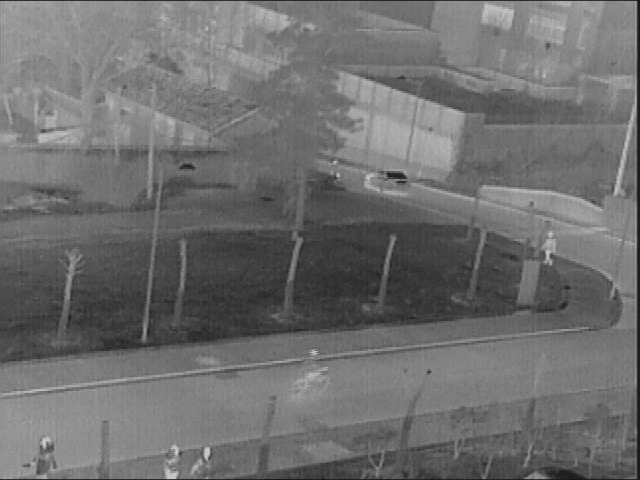

Supplement: S1 File — (ZIP) [file pone.0173613.s001.zip › infrared car and bicycle set/V20624.bmp]

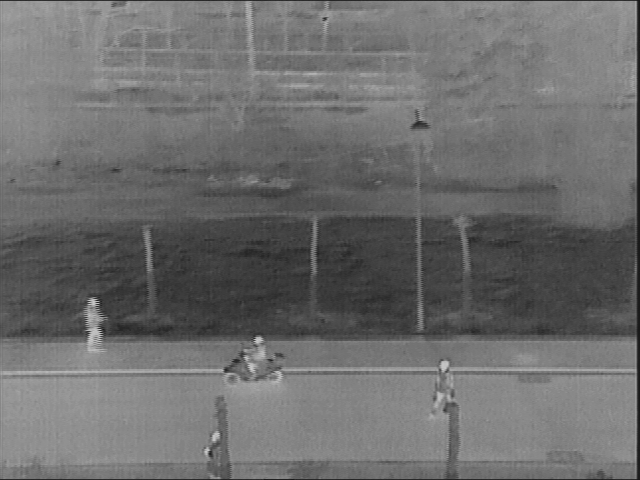

Supplement: S1 File — (ZIP) [file pone.0173613.s001.zip › infrared car and bicycle set/V20633.bmp]

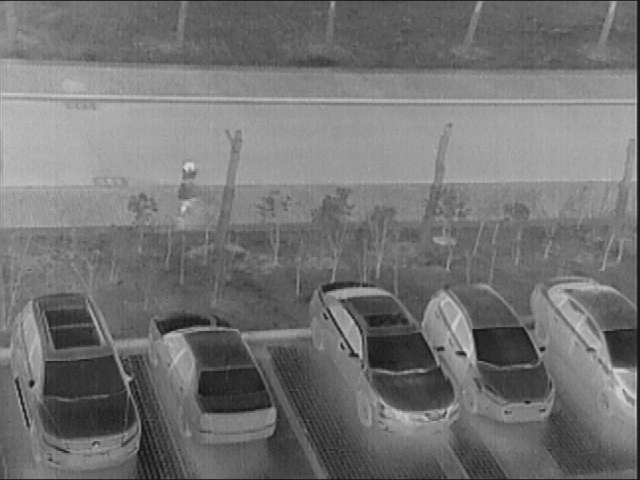

Supplement: S1 File — (ZIP) [file pone.0173613.s001.zip › infrared car and bicycle set/V20636.bmp]

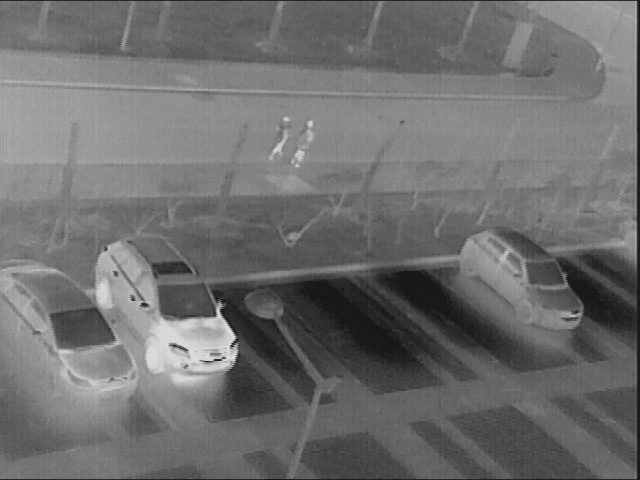

Supplement: S1 File — (ZIP) [file pone.0173613.s001.zip › infrared car and bicycle set/V20639.bmp]

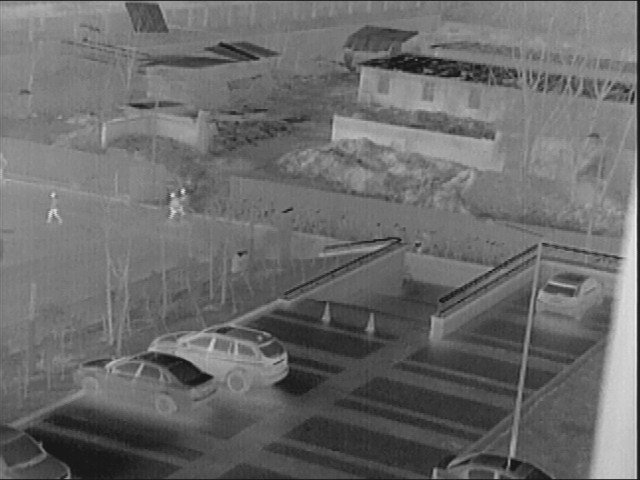

Supplement: S1 File — (ZIP) [file pone.0173613.s001.zip › infrared car and bicycle set/V20680.bmp]

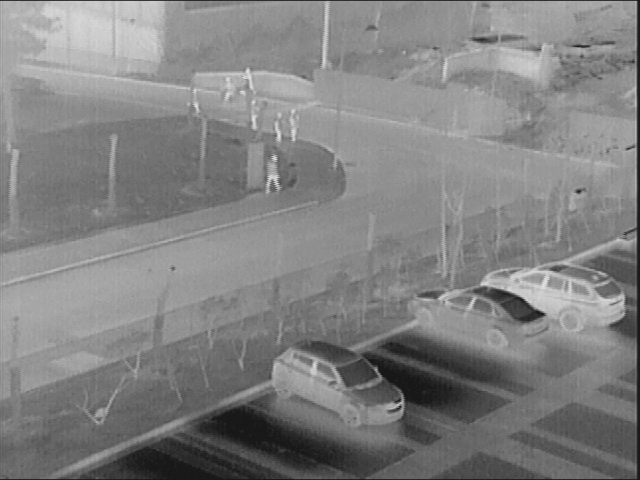

Supplement: S1 File — (ZIP) [file pone.0173613.s001.zip › infrared car and bicycle set/V20691.bmp]

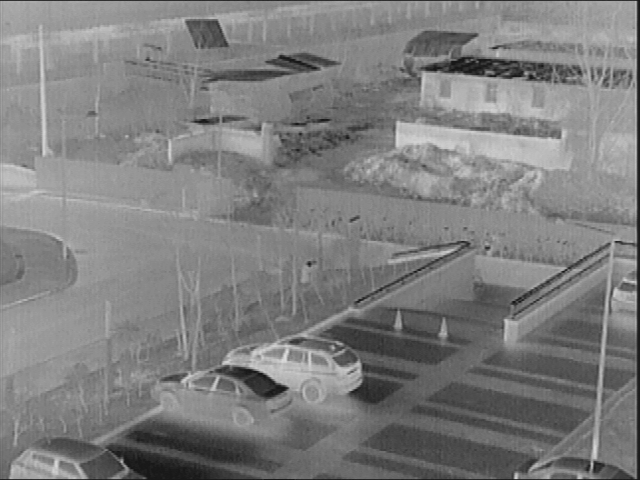

Supplement: S1 File — (ZIP) [file pone.0173613.s001.zip › infrared car and bicycle set/V20704.bmp]

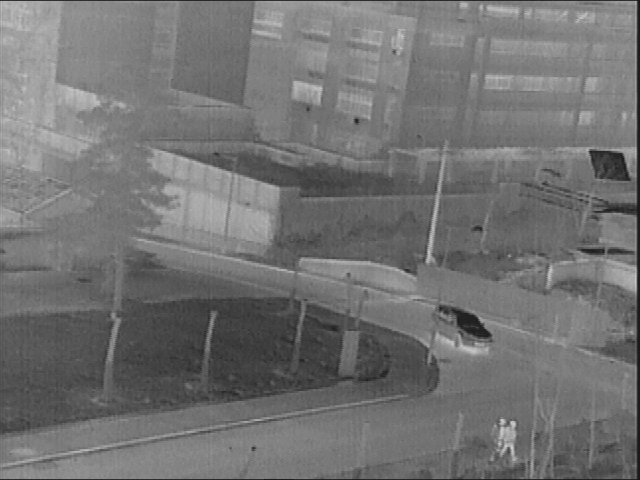

Supplement: S1 File — (ZIP) [file pone.0173613.s001.zip › infrared car and bicycle set/V20748.bmp]

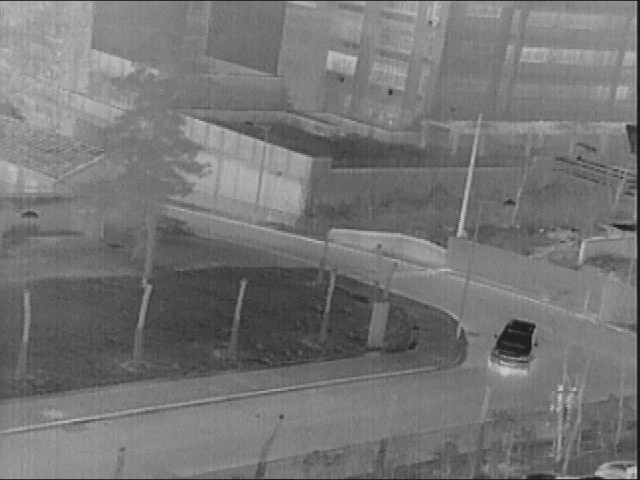

Supplement: S1 File — (ZIP) [file pone.0173613.s001.zip › infrared car and bicycle set/V20751.bmp]

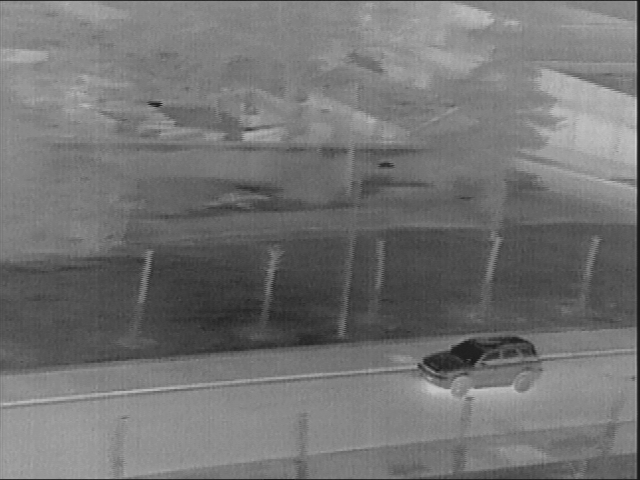

Supplement: S1 File — (ZIP) [file pone.0173613.s001.zip › infrared car and bicycle set/V20757.bmp]

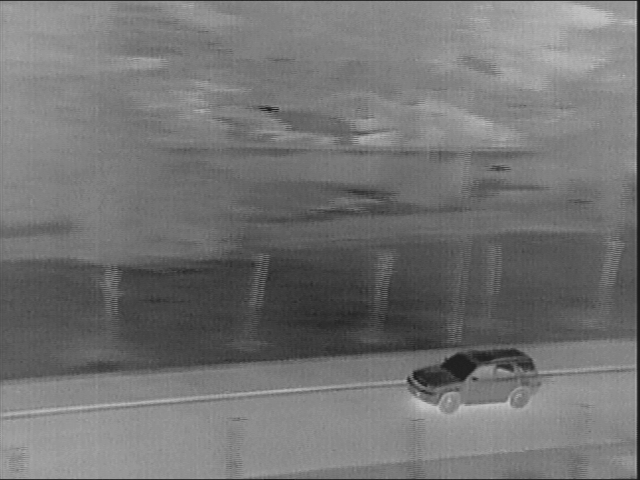

Supplement: S1 File — (ZIP) [file pone.0173613.s001.zip › infrared car and bicycle set/V20758.bmp]

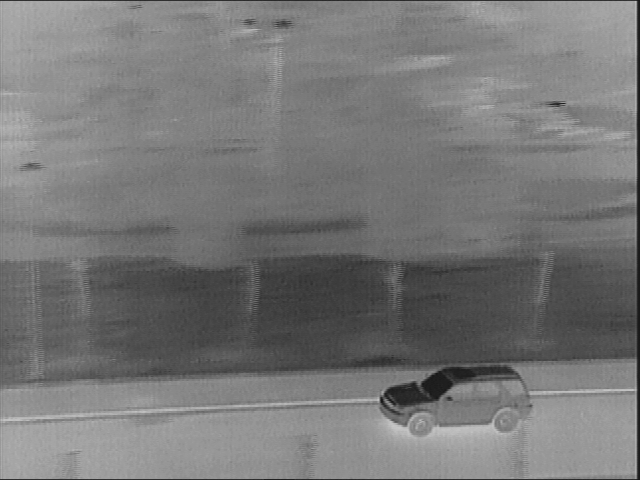

Supplement: S1 File — (ZIP) [file pone.0173613.s001.zip › infrared car and bicycle set/V20760.bmp]

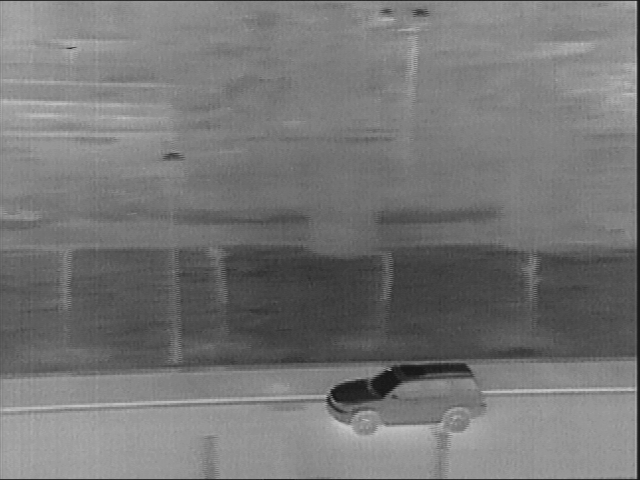

Supplement: S1 File — (ZIP) [file pone.0173613.s001.zip › infrared car and bicycle set/V20761.bmp]

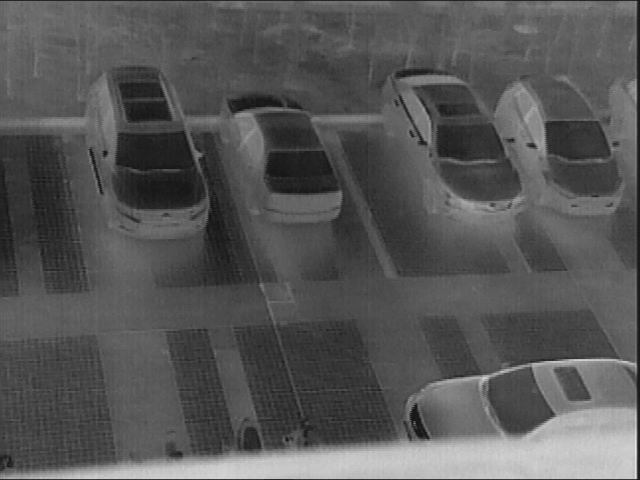

Supplement: S1 File — (ZIP) [file pone.0173613.s001.zip › infrared car and bicycle set/V20834.bmp]

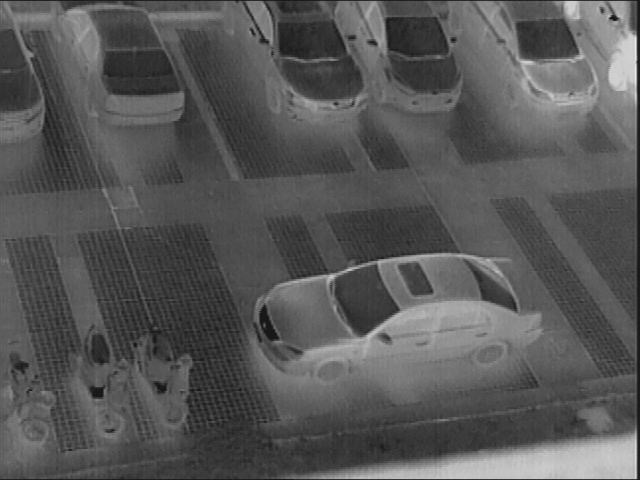

Supplement: S1 File — (ZIP) [file pone.0173613.s001.zip › infrared car and bicycle set/V20840.bmp]

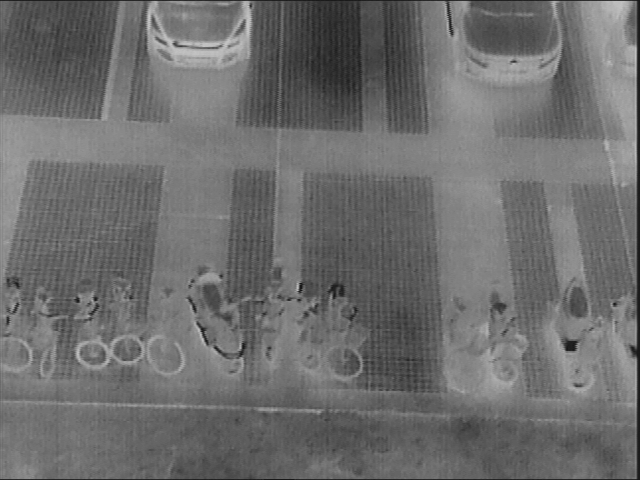

Supplement: S1 File — (ZIP) [file pone.0173613.s001.zip › infrared car and bicycle set/V20859.bmp]

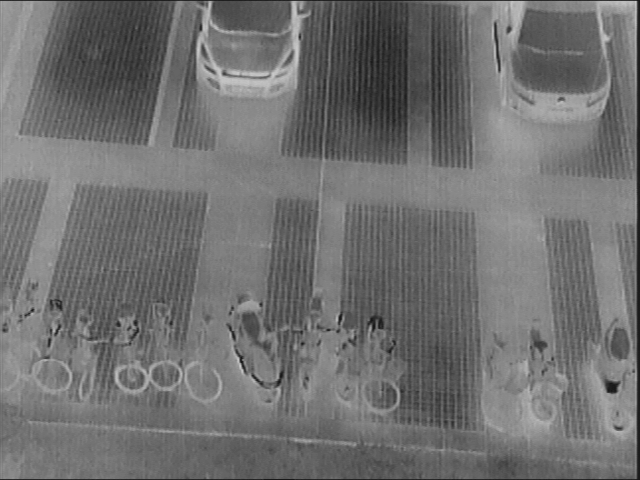

Supplement: S1 File — (ZIP) [file pone.0173613.s001.zip › infrared car and bicycle set/V20861.bmp]

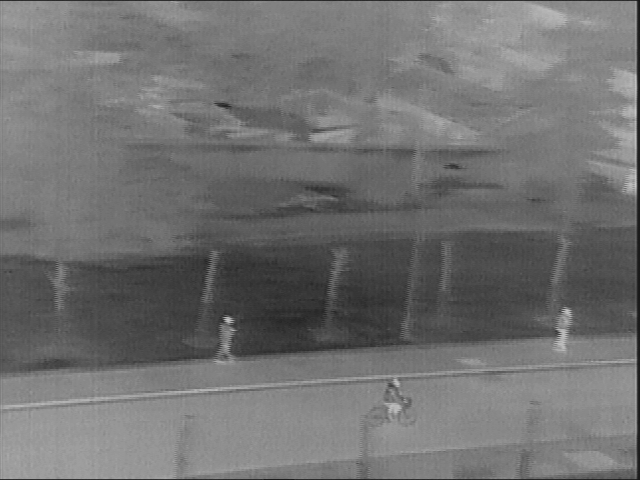

Supplement: S1 File — (ZIP) [file pone.0173613.s001.zip › infrared car and bicycle set/V20885.bmp]

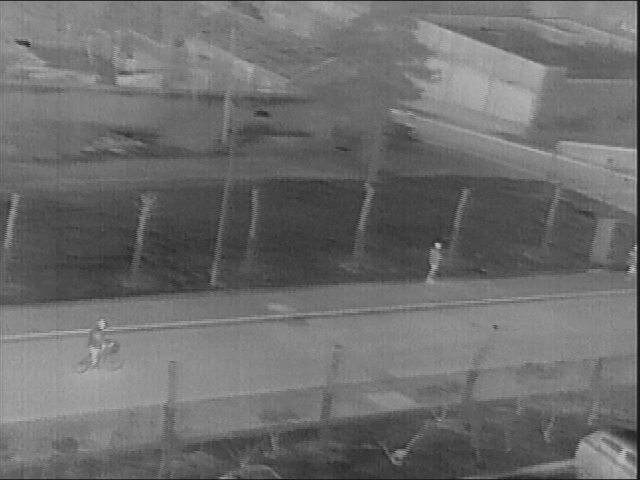

Supplement: S1 File — (ZIP) [file pone.0173613.s001.zip › infrared car and bicycle set/V20908.bmp]

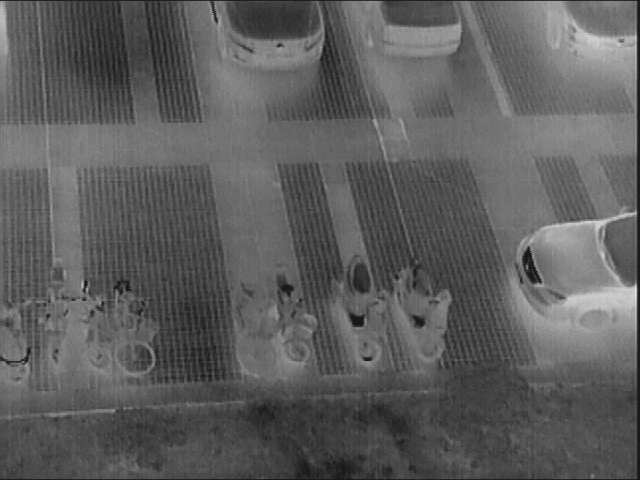

Supplement: S1 File — (ZIP) [file pone.0173613.s001.zip › infrared car and bicycle set/V20919.bmp]

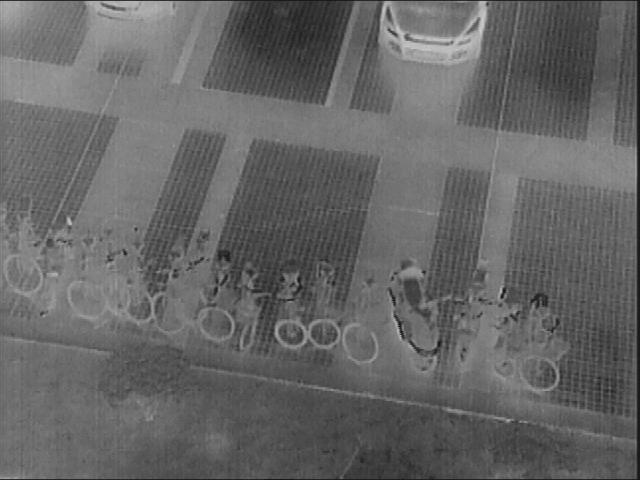

Supplement: S1 File — (ZIP) [file pone.0173613.s001.zip › infrared car and bicycle set/V20928.bmp]

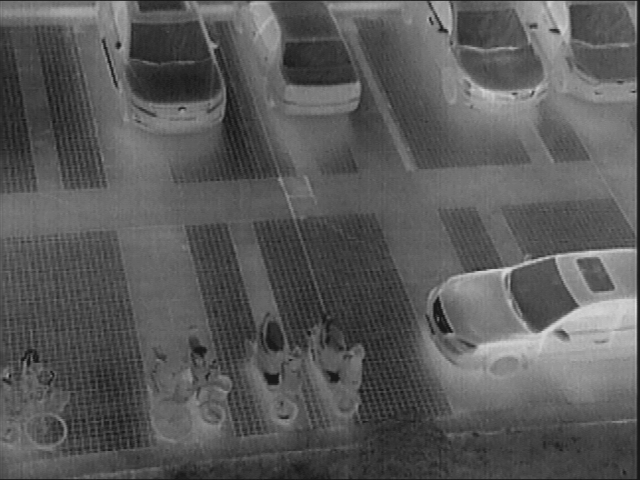

Supplement: S1 File — (ZIP) [file pone.0173613.s001.zip › infrared car and bicycle set/V20934.bmp]

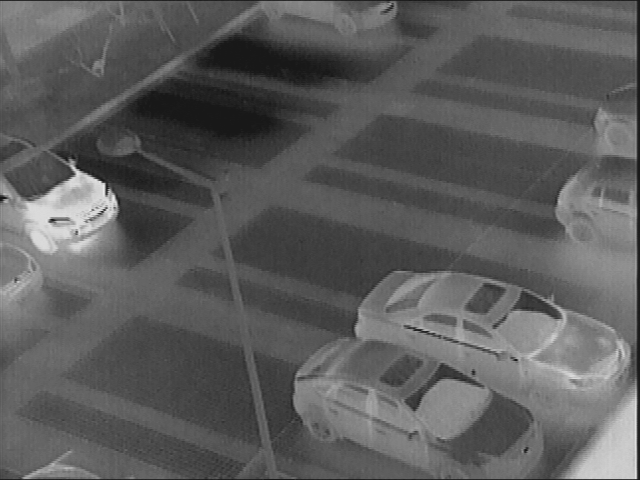

Supplement: S1 File — (ZIP) [file pone.0173613.s001.zip › infrared car and bicycle set/V20948.bmp]

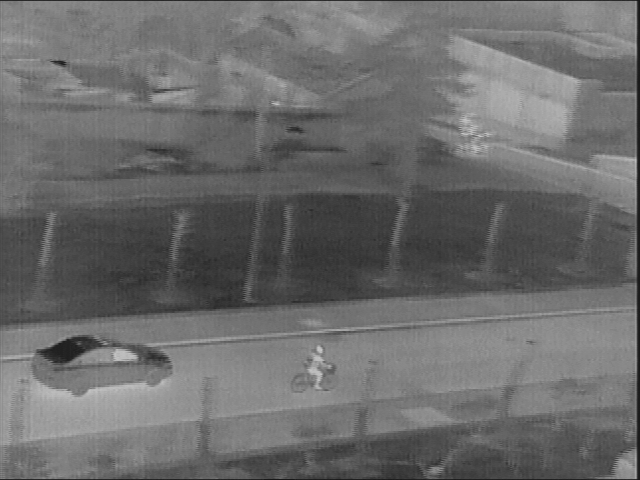

Supplement: S1 File — (ZIP) [file pone.0173613.s001.zip › infrared car and bicycle set/V20983.bmp]

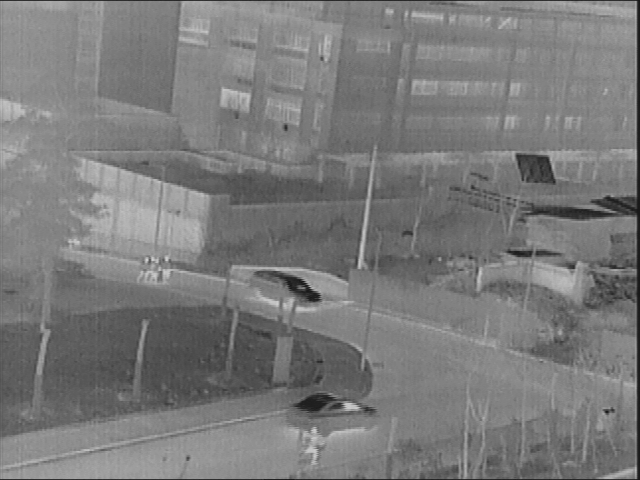

Supplement: S1 File — (ZIP) [file pone.0173613.s001.zip › infrared car and bicycle set/V20989.bmp]

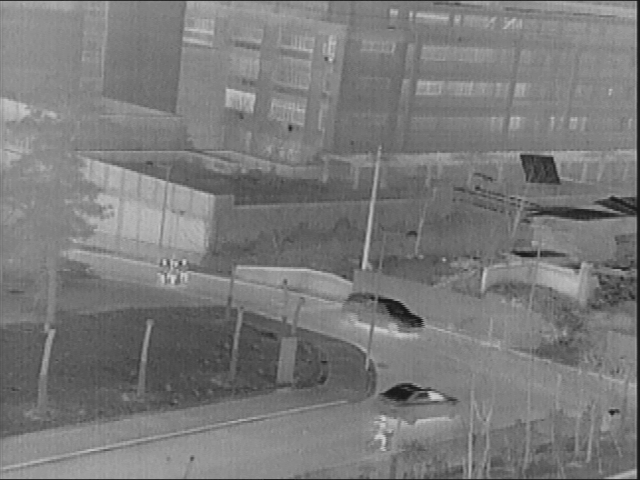

Supplement: S1 File — (ZIP) [file pone.0173613.s001.zip › infrared car and bicycle set/V20991.bmp]

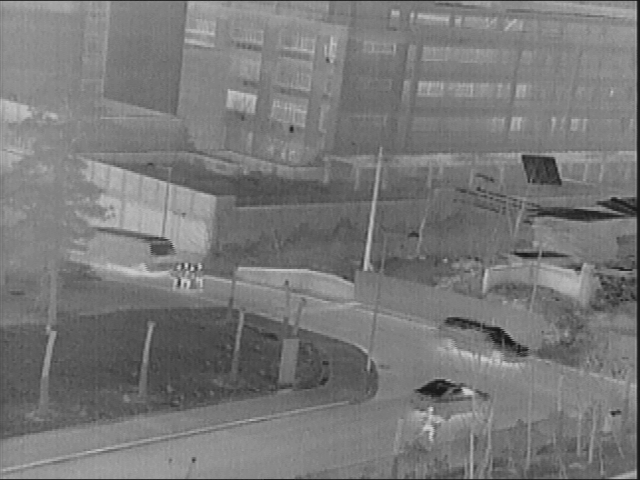

Supplement: S1 File — (ZIP) [file pone.0173613.s001.zip › infrared car and bicycle set/V20993.bmp]

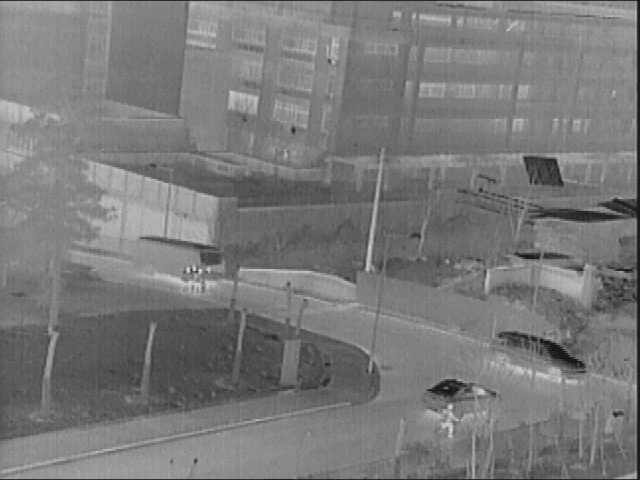

Supplement: S1 File — (ZIP) [file pone.0173613.s001.zip › infrared car and bicycle set/V20994.bmp]

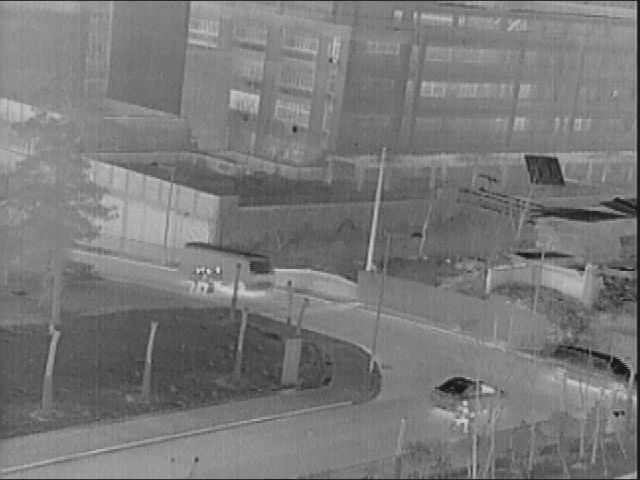

Supplement: S1 File — (ZIP) [file pone.0173613.s001.zip › infrared car and bicycle set/V20995.bmp]

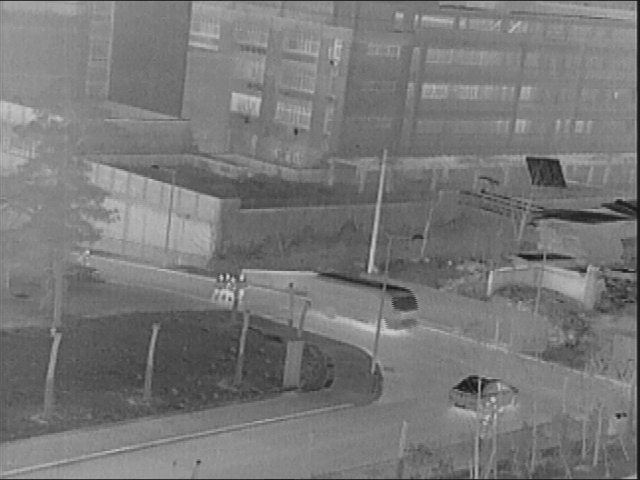

Supplement: S1 File — (ZIP) [file pone.0173613.s001.zip › infrared car and bicycle set/V20998.bmp]

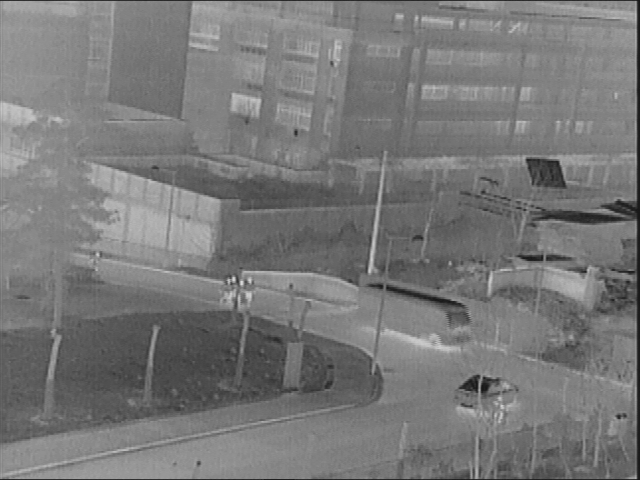

Supplement: S1 File — (ZIP) [file pone.0173613.s001.zip › infrared car and bicycle set/V20999.bmp]

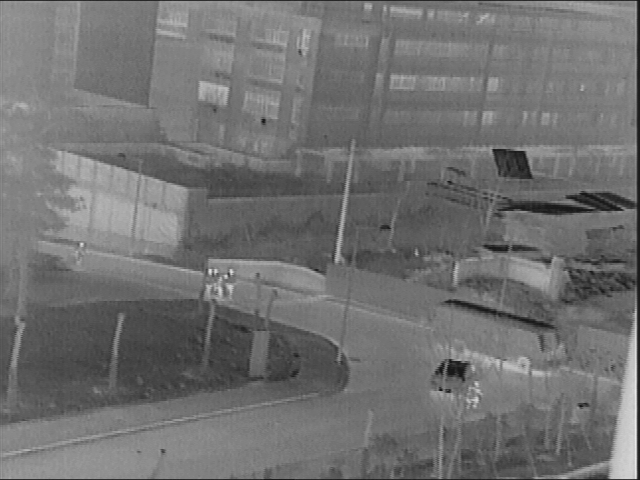

Supplement: S1 File — (ZIP) [file pone.0173613.s001.zip › infrared car and bicycle set/V21001.bmp]

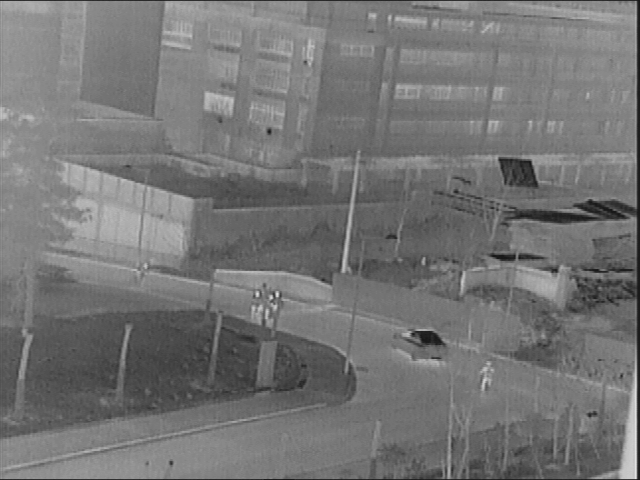

Supplement: S1 File — (ZIP) [file pone.0173613.s001.zip › infrared car and bicycle set/V21007.bmp]

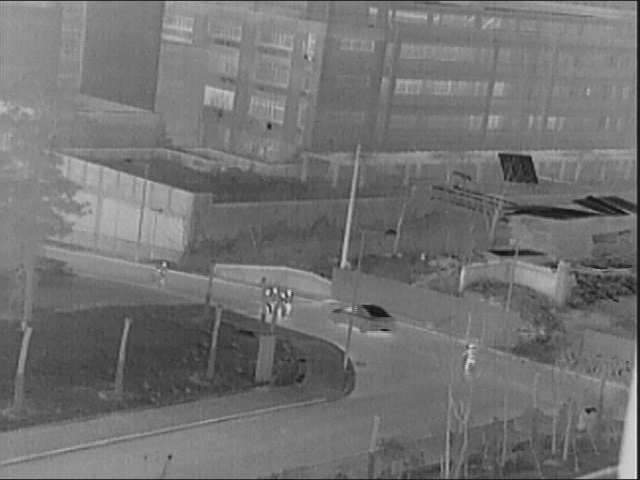

Supplement: S1 File — (ZIP) [file pone.0173613.s001.zip › infrared car and bicycle set/V21009.bmp]

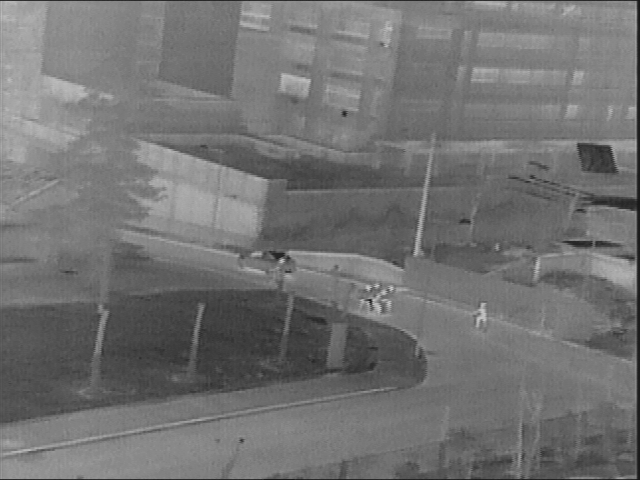

Supplement: S1 File — (ZIP) [file pone.0173613.s001.zip › infrared car and bicycle set/V21013.bmp]

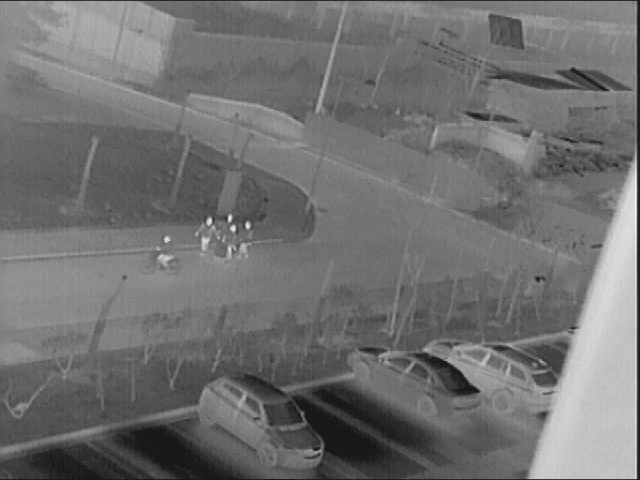

Supplement: S1 File — (ZIP) [file pone.0173613.s001.zip › infrared car and bicycle set/V21046.bmp]

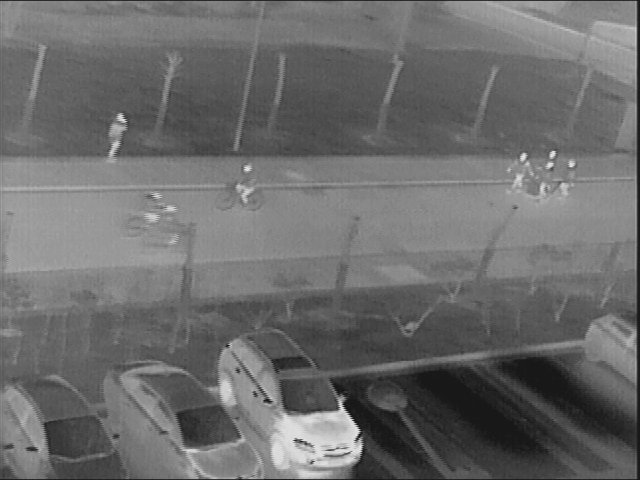

Supplement: S1 File — (ZIP) [file pone.0173613.s001.zip › infrared car and bicycle set/V21052.bmp]

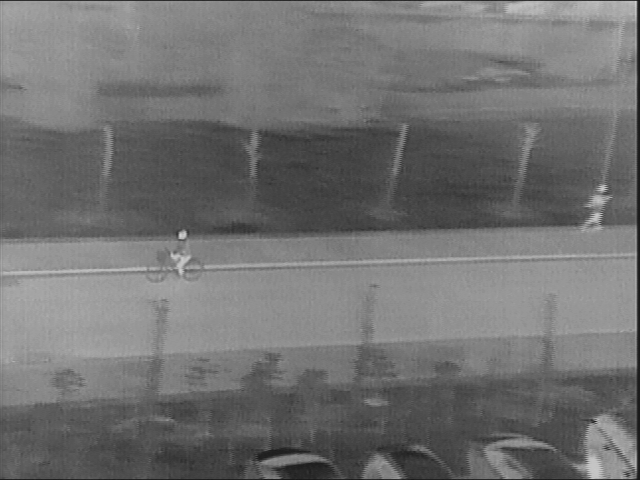

Supplement: S1 File — (ZIP) [file pone.0173613.s001.zip › infrared car and bicycle set/V21058.bmp]

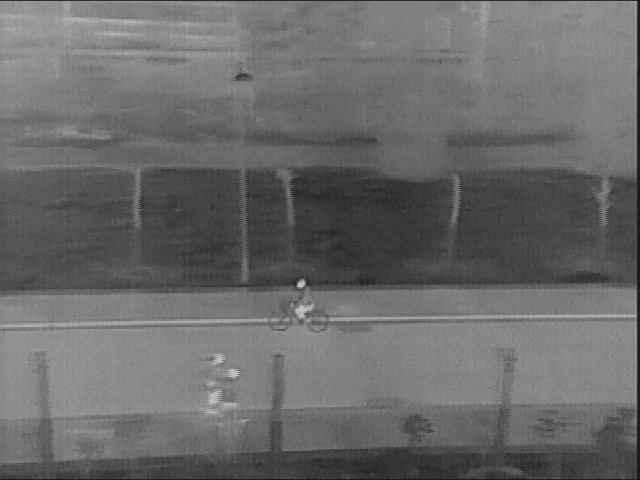

Supplement: S1 File — (ZIP) [file pone.0173613.s001.zip › infrared car and bicycle set/V21061.bmp]

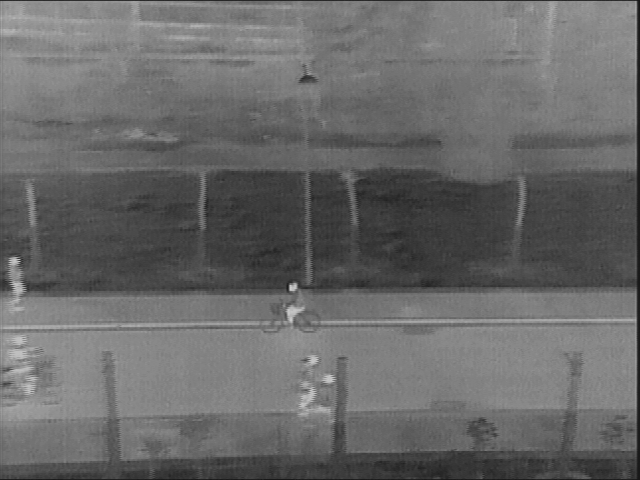

Supplement: S1 File — (ZIP) [file pone.0173613.s001.zip › infrared car and bicycle set/V21062.bmp]

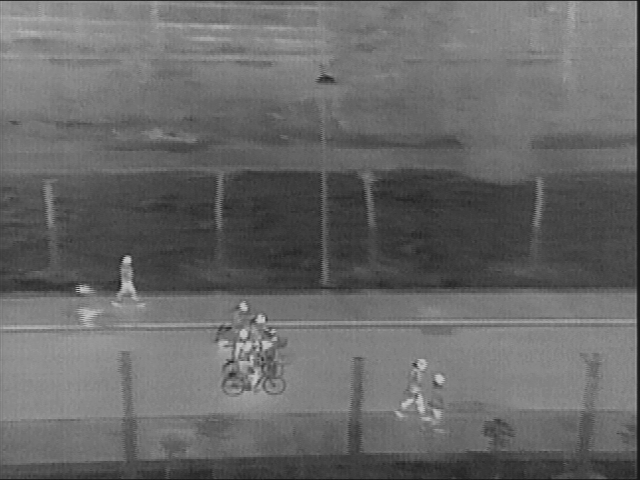

Supplement: S1 File — (ZIP) [file pone.0173613.s001.zip › infrared car and bicycle set/V21065.bmp]

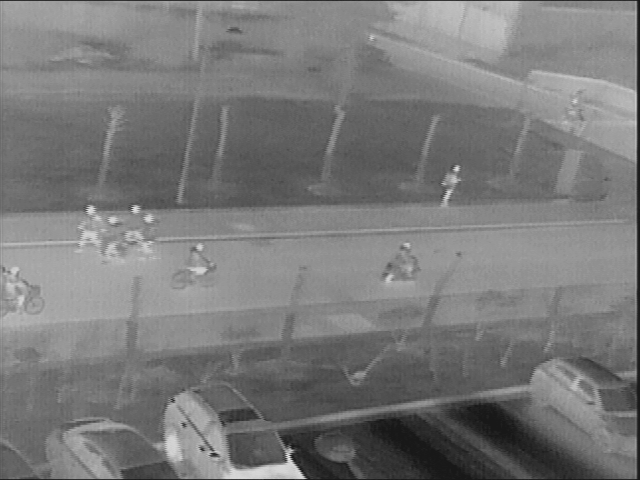

Supplement: S1 File — (ZIP) [file pone.0173613.s001.zip › infrared car and bicycle set/V21074.bmp]

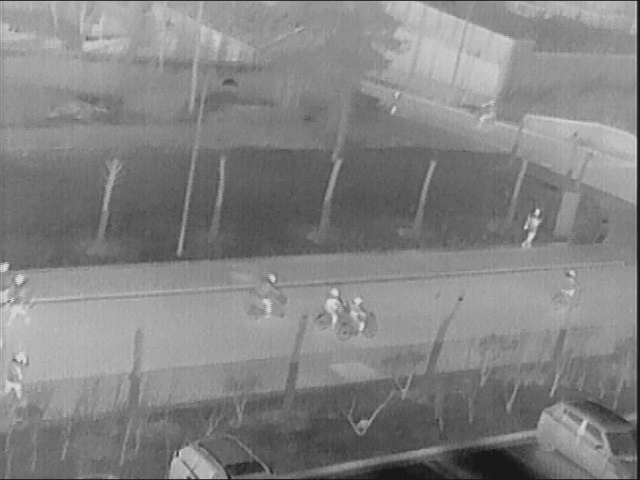

Supplement: S1 File — (ZIP) [file pone.0173613.s001.zip › infrared car and bicycle set/V21080.bmp]

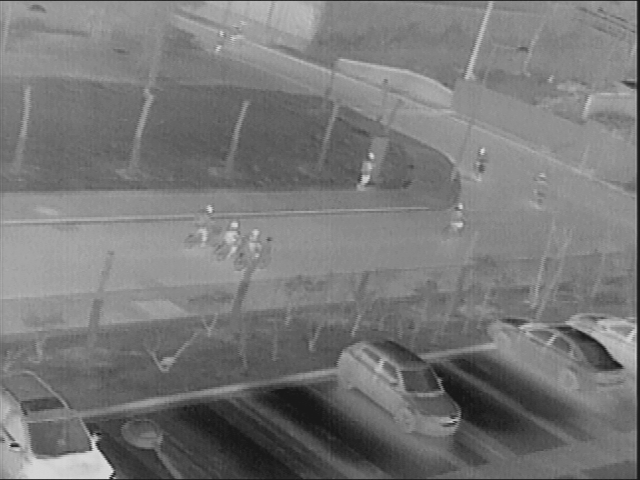

Supplement: S1 File — (ZIP) [file pone.0173613.s001.zip › infrared car and bicycle set/V21082.bmp]

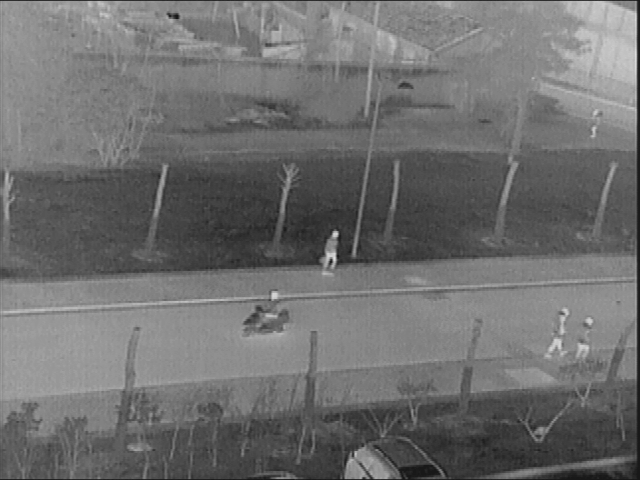

Supplement: S1 File — (ZIP) [file pone.0173613.s001.zip › infrared car and bicycle set/V21098.bmp]

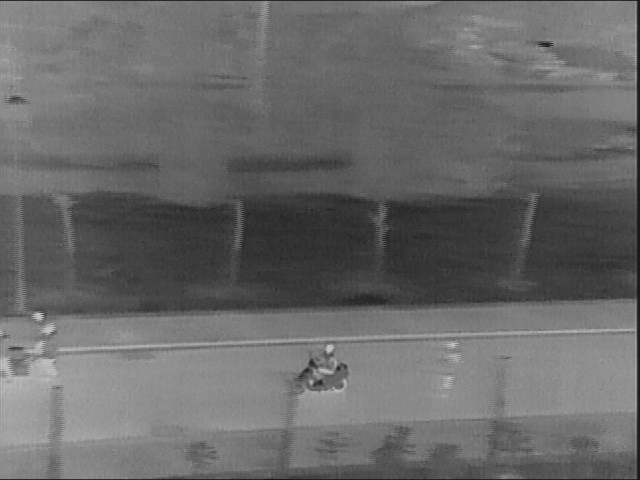

Supplement: S1 File — (ZIP) [file pone.0173613.s001.zip › infrared car and bicycle set/V21101.bmp]

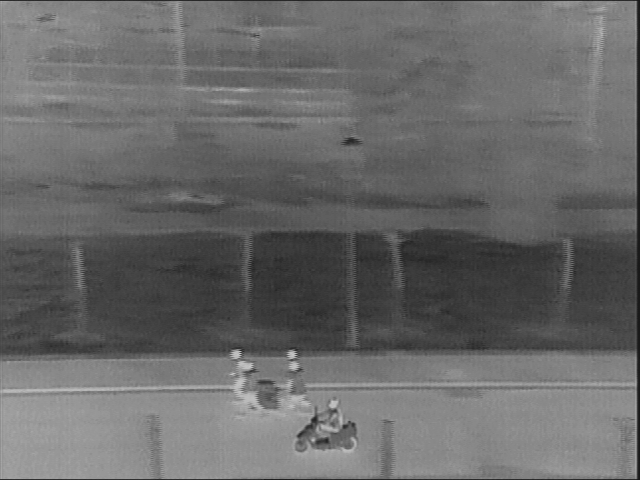

Supplement: S1 File — (ZIP) [file pone.0173613.s001.zip › infrared car and bicycle set/V21104.bmp]

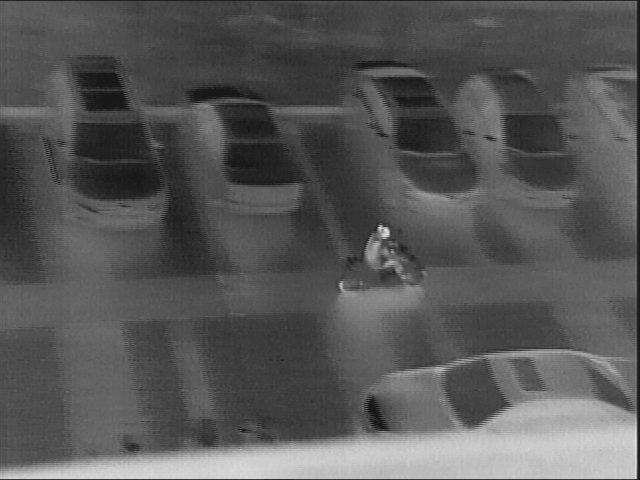

Supplement: S1 File — (ZIP) [file pone.0173613.s001.zip › infrared car and bicycle set/V21131.bmp]

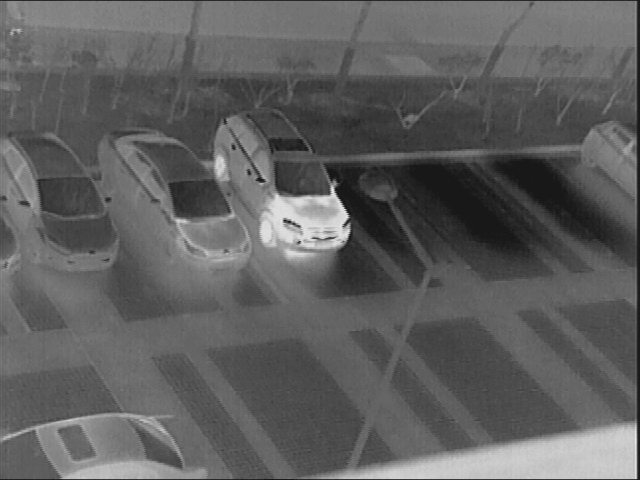

Supplement: S1 File — (ZIP) [file pone.0173613.s001.zip › infrared car and bicycle set/V21137.bmp]

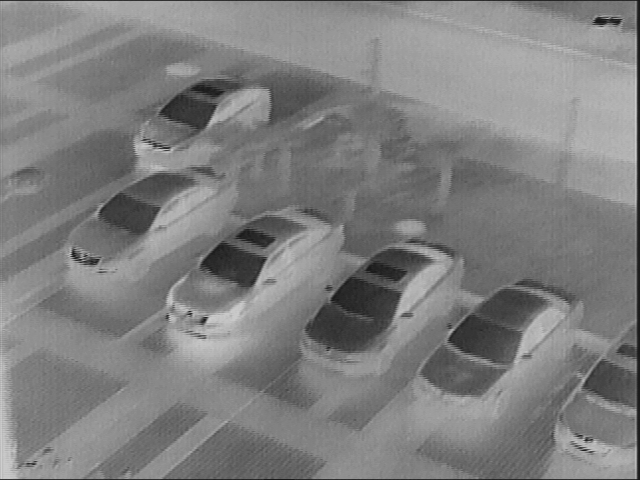

Supplement: S1 File — (ZIP) [file pone.0173613.s001.zip › infrared car and bicycle set/V21256.bmp]

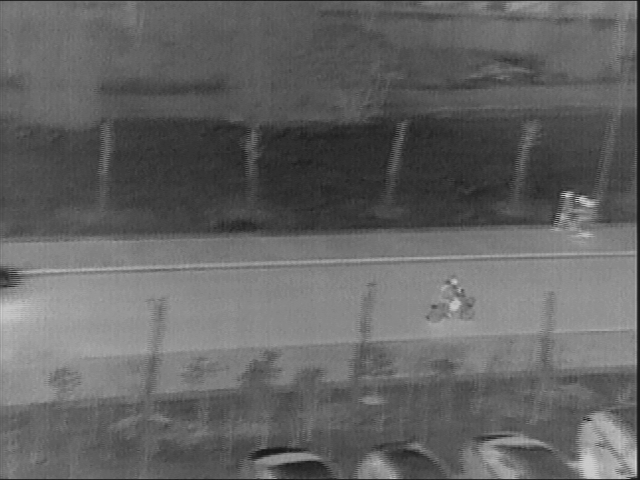

Supplement: S1 File — (ZIP) [file pone.0173613.s001.zip › infrared car and bicycle set/V21263.bmp]

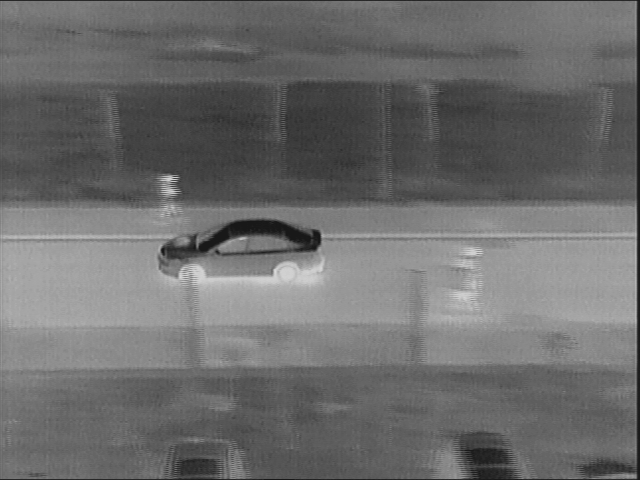

Supplement: S1 File — (ZIP) [file pone.0173613.s001.zip › infrared car and bicycle set/V21264.bmp]

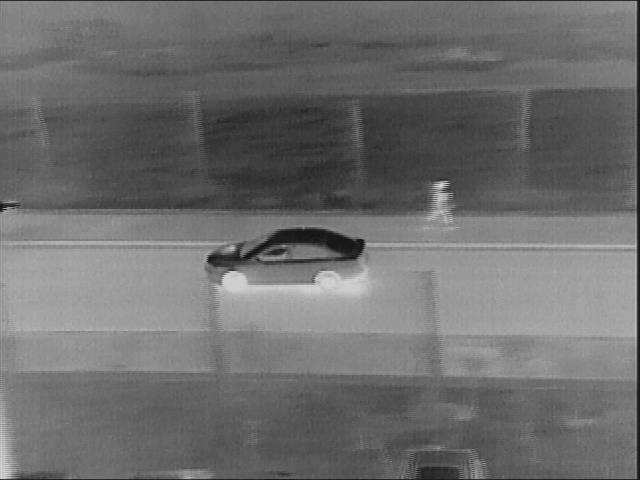

Supplement: S1 File — (ZIP) [file pone.0173613.s001.zip › infrared car and bicycle set/V21265.bmp]

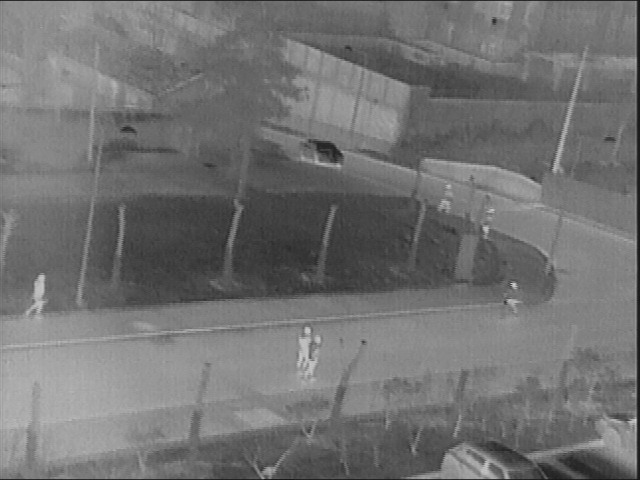

Supplement: S1 File — (ZIP) [file pone.0173613.s001.zip › infrared car and bicycle set/V21297.bmp]

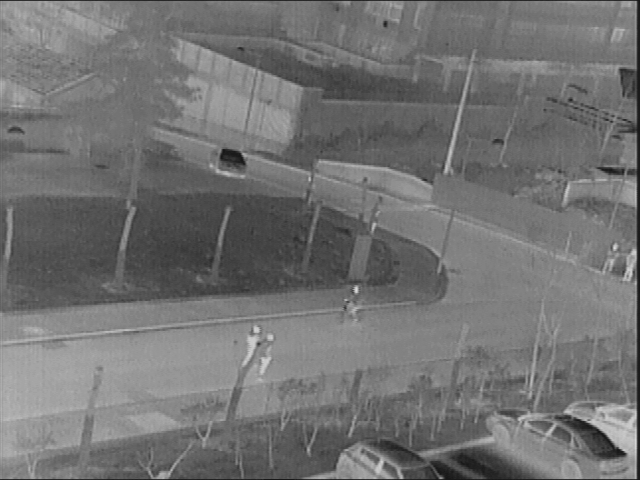

Supplement: S1 File — (ZIP) [file pone.0173613.s001.zip › infrared car and bicycle set/V21300.bmp]

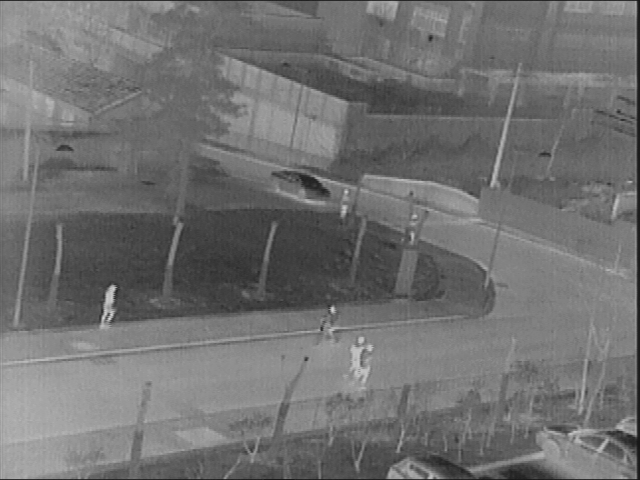

Supplement: S1 File — (ZIP) [file pone.0173613.s001.zip › infrared car and bicycle set/V21303.bmp]

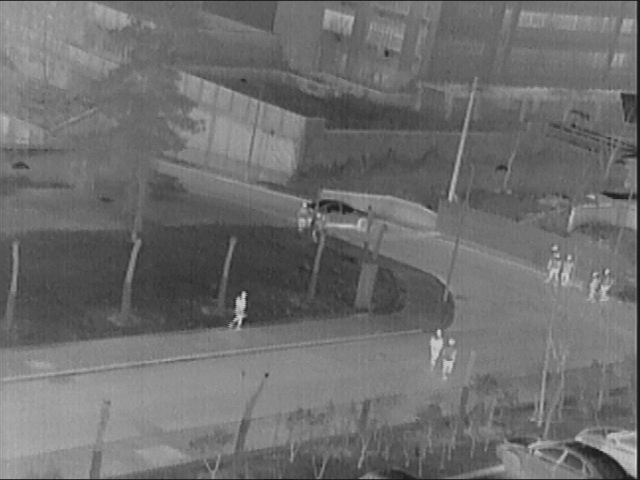

Supplement: S1 File — (ZIP) [file pone.0173613.s001.zip › infrared car and bicycle set/V21312.bmp]

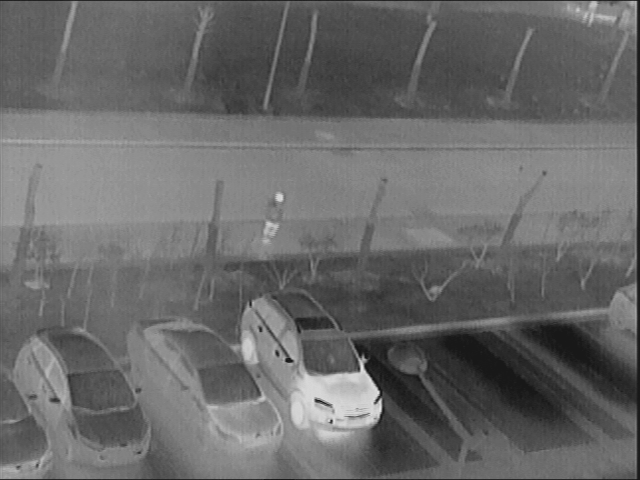

Supplement: S1 File — (ZIP) [file pone.0173613.s001.zip › infrared car and bicycle set/V21344.bmp]

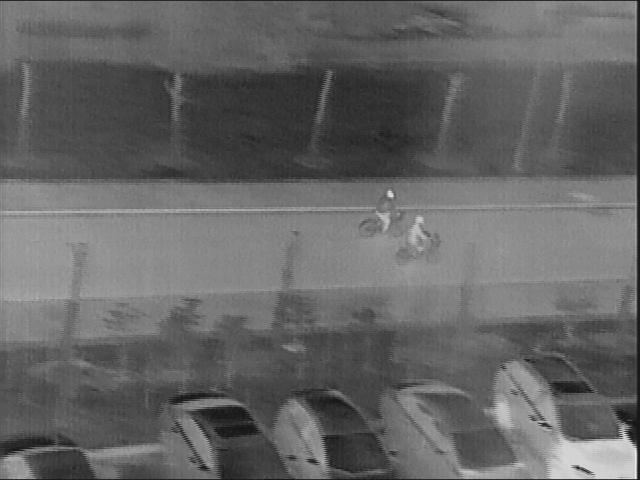

Supplement: S1 File — (ZIP) [file pone.0173613.s001.zip › infrared car and bicycle set/V21349.bmp]

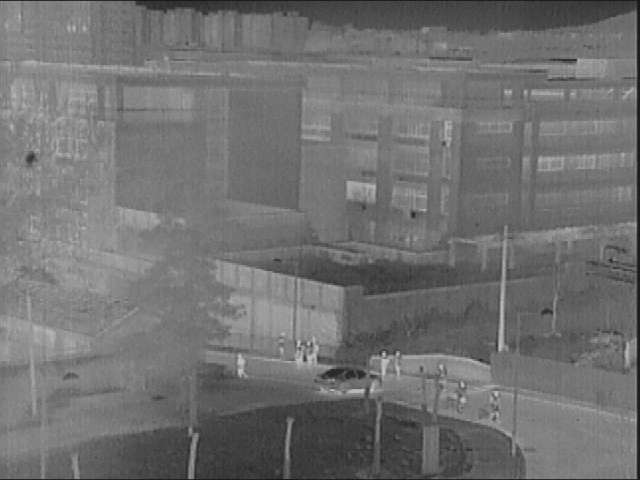

Supplement: S1 File — (ZIP) [file pone.0173613.s001.zip › infrared car and bicycle set/V21358.bmp]

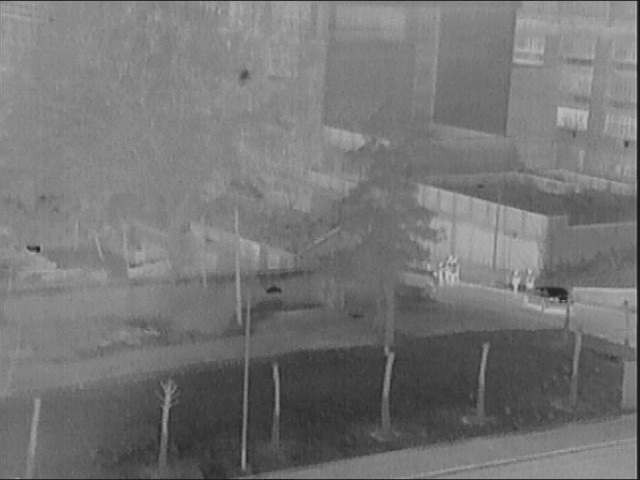

Supplement: S1 File — (ZIP) [file pone.0173613.s001.zip › infrared car and bicycle set/V21367.bmp]

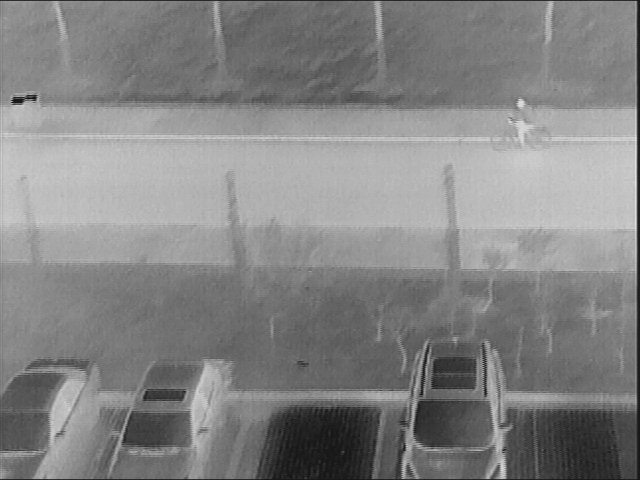

Supplement: S1 File — (ZIP) [file pone.0173613.s001.zip › infrared car and bicycle set/V21411.bmp]

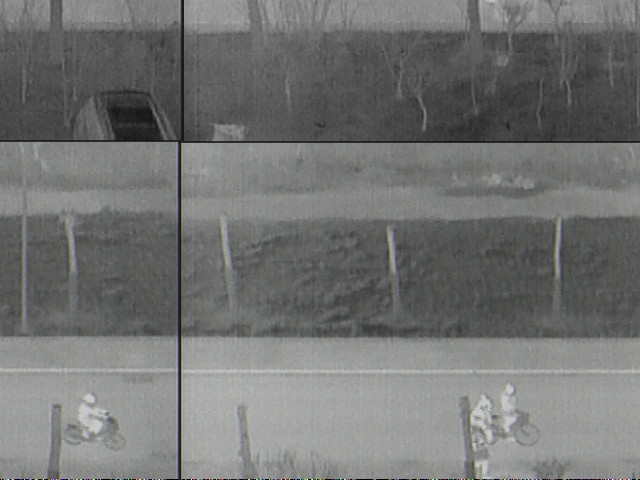

Supplement: S1 File — (ZIP) [file pone.0173613.s001.zip › infrared car and bicycle set/V34438.bmp]

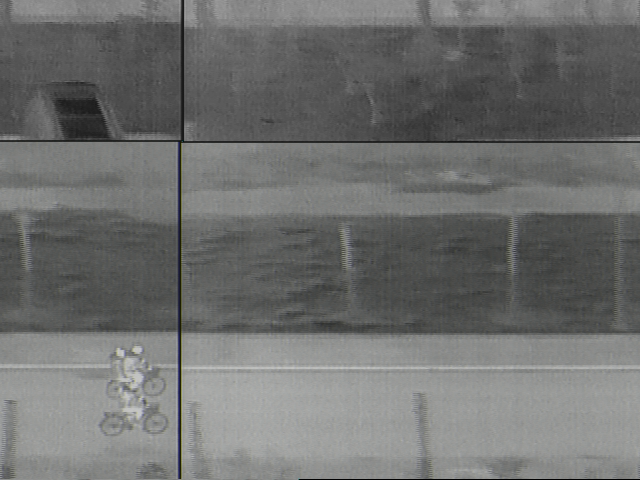

Supplement: S1 File — (ZIP) [file pone.0173613.s001.zip › infrared car and bicycle set/V34461.bmp]
